# Bi-PE: bi-directional priming improves CRISPR/Cas9 prime editing in mammalian cells

**DOI:** 10.1093/nar/gkac506

**Published:** 2022-06-10

**Authors:** Rui Tao, Yanhong Wang, Yaoge Jiao, Yun Hu, Li Li, Lurong Jiang, Lifang Zhou, Junyan Qu, Qiang Chen, Shaohua Yao

**Affiliations:** Laboratory of Biotherapy, National Key Laboratory of Biotherapy, Cancer Center, West China Hospital, Sichuan university, Renmin Nanlu 17, Chengdu 610041, Sichuan, China; Laboratory of Biotherapy, National Key Laboratory of Biotherapy, Cancer Center, West China Hospital, Sichuan university, Renmin Nanlu 17, Chengdu 610041, Sichuan, China; Laboratory of Biotherapy, National Key Laboratory of Biotherapy, Cancer Center, West China Hospital, Sichuan university, Renmin Nanlu 17, Chengdu 610041, Sichuan, China; Laboratory of Biotherapy, National Key Laboratory of Biotherapy, Cancer Center, West China Hospital, Sichuan university, Renmin Nanlu 17, Chengdu 610041, Sichuan, China; Laboratory of Biotherapy, National Key Laboratory of Biotherapy, Cancer Center, West China Hospital, Sichuan university, Renmin Nanlu 17, Chengdu 610041, Sichuan, China; Laboratory of Biotherapy, National Key Laboratory of Biotherapy, Cancer Center, West China Hospital, Sichuan university, Renmin Nanlu 17, Chengdu 610041, Sichuan, China; Laboratory of Biotherapy, National Key Laboratory of Biotherapy, Cancer Center, West China Hospital, Sichuan university, Renmin Nanlu 17, Chengdu 610041, Sichuan, China; Center of Infectious Disease, West China Hospital, Sichuan University, Renmin Nanlu 17, Chengdu 610041, Sichuan, China; Laboratory of Biotherapy, National Key Laboratory of Biotherapy, Cancer Center, West China Hospital, Sichuan university, Renmin Nanlu 17, Chengdu 610041, Sichuan, China; Laboratory of Biotherapy, National Key Laboratory of Biotherapy, Cancer Center, West China Hospital, Sichuan university, Renmin Nanlu 17, Chengdu 610041, Sichuan, China

## Abstract

Prime editors consisting of Cas9-nickase and reverse transcriptase enable targeted precise editing of small DNA pieces, including all 12 kinds of base substitutions, insertions and deletions, while without requiring double-strand breaks or donor templates. Current optimized prime editing strategy (PE3) uses two guide RNAs to guide the performance of prime editor. One guide RNA carrying both spacer and templating sequences (pegRNA) guides prime editor to produce ssDNA break and subsequent extension, and the other one produces a nick in the complementary strand. Here, we demonstrated that positioning the nick sgRNA nearby the templating sequences of the pegRNA facilitated targeted large fragment deletion and that engineering both guide RNAs to be pegRNAs to achieve bi-direction prime editing (Bi-PE) further increase the efficiency by up to 16 times and improved the accuracy of editing products by 60 times. In addition, we showed that Bi-PE strategy also increased the efficiency of simultaneous conversion of multiple bases but not single base conversion over PE3. In conclusion, Bi-PE strategy expanded the editing scope and improved the efficiency and the accuracy of prime editing system, which might have a wide range of potential applications.

## INTRODUCTION

The clustered regularly interspaced short palindromic repeats associated (CRISPR-Cas) system is an immune system that is frequently used by bacteria and archaea to prevent infection of foreign phages and plasmids (1). The CRISPR systems, especially Cas9 systems, are readily reprogrammable for genome editing purpose, providing powerful tools for basic biomedical research and clinical translation (2–5). Recently, the discovery of prime editing (PE) tool enables targeted base conversions or introduction of small-sized genetic change, in a precise and irreversible way, while without causing robust DSBs (6), making it practicable to correct small pieces of genetic lesions in inherited diseases (7).

Although showing great potential in a wide range of applications, current PE system is less efficient, especially when making large insertions and deletions in the genomes, which is one of the biggest roadblocks towards its application. Currently, the optimized version of prime editing system, PE3, consists of four parts: (i) Cas9 endonuclease which nicks DNA; (ii) the prime editing guide RNA (pegRNA) which guides prime editor to the DNA target to produce ssDNA break and provide prime binding sequences (PBS) and RT-template for the nicked DNA; (iii) the reverse transcriptase (RT) which transcribes DNA from pegRNA template, and 4. the nick sgRNA that guide the editor to nick the non-edited strand (6). Concerted actions of these four parts generate two DNA lesions around the target site, a single strand DNA break in the edited strand whose 3′ end was extended by RT and a pure ssDNA break in the non-edited strand. The 3′ end extension of the edited strand was templated by pegRNA that was designed to contain PBS, the edits and a homology arm (HA) that is homologous to the DNA sequence downstream of the site to be edited (6). Fixation of these DNA lesions either by DNA repair or by DNA replication mechanisms results in the integration of the 3′ end extension into the genome (8–11).

Although the detailed fixation process remains unknown, the outcomes of prime editing suggested that the newly reverse transcribed ssDNA carrying edits must replace the original sequences, which resembled the process of ssDNA invasion and flap cleavage during homology directed double strand break (DSB) repair (12,13). In mammalian cells, the invasion is a complicated process and requires multiple factors that function as binding partners to stabilize the end of invading ssDNA or as exchange factor to direct homology searches. As many of those factors, such as RPA, Rad51 and BRCA1, were recruited by DNA breaks (12–17), we hypothesized that positioning the nick sgRNA of the PE system nearby the HA would facilitate its homology search and subsequent invasion, thereby improving large fragment deletion. A systematic examination of the positions of the ssDNA break in the non-edited strand revealed that only the breaks nearby the HA were able to induce detectable targeted deletions of large DNA fragments, ranging from hundreds to thousands of base pairs. Importantly, when engineering the nick sgRNA to a second pegRNA to achieve bi-directional prime editing (Bi-PE), the efficiency of large fragment deletion was further improved. The Bi-PE strategy was also efficient in multiplex base conversions, fragment replacement, and simultaneous insertion of paired LoxP sites in the same allele. Importantly, the editing products of Bi-PE strategy contained much lower undesired indels as compared to PE3. Therefore, Bi-PE strategy expanded the editing scope and improved the efficiency and the accuracy of prime editing system.

## MATERIALS AND METHODS

### Plasmid construction

PE2 plasmid was obtained from addgene (#132775). sgRNA plasmids were constructed through inserting oligos containing desired spacers into Bbs1 digested empty plasmids. pegRNA plasmids were generated by PCR amplification of existing sgRNA using reverse primers containing PBS, edits and HA sequences. Sequences of pegRNAs were listed in Supplementary Table S1. Oligos used to generate sgRNAs were listed in Supplementary Table S2. All plasmids were verified by Sanger sequencing.

### Cell culture and transfection

HEK293T were cultured in Dulbecco's modified Eagle's medium (Thermo Fisher Scientific), supplemented with 10% (v/v) fetal bovine serum (Life Technologies) and 1% penicillin/streptomycin (Boster Biological Technology Co. Ltd.), and maintained at 37°C with 5% CO_2_. Cells were seeded onto 96-well plate (BIOFIL). Twenty-four hours after seeding, cells at a confluence of ∼70–80% were transfected with 0.7 ul of Transeasy™ (Forgene) and 276 ng of PE2 plasmid DNA, 62 ng of pegRNA1 plasmid DNA and 62 ng of pegRNA2 plasmid DNA (for Bi-PE and Cas9 nickase transfections); 276 ng of PE2 plasmid DNA, 93 ng of pegRNA plasmid DNA and 31 ng of sgRNA plasmid DNA (for PE3 transfections); or 276 ng of Cas9 plasmid DNA and 62 ng of sgRNA 1 plasmid DNA and 62 ng of sgRNA2 plasmid DNA (for paired Cas9 nuclease and Cas9 nickase transfections), according to the manufacturer's protocol.

### Detecting the deletion or replacement efficiency via agarose gel electrophoresis

Seventy-two hours post-transfection, genomic DNA was extracted by the addition of 50 μl of freshly made lysis buffer into each well of the 96-well plate. The lysate was incubated at 55°C for 10 min and was heat-inactivated at 95°C for another 10 min. Then the genomic DNA was subjected to PCR analysis using Phanta^®^ Max Super-Fidelity DNA Polymerase (Vazyme). The PCR reaction was performed with 1μl genomic DNA, and 0.2 μM of forward and reverse primers in a final volume of 30 μl. Primers flanking the deletion region were listed in (Supplementary Table S3). The amplicons were separated by agarose gel electrophoresis and gray values of armed bands were analyzed by Adobe Photoshop CC (2019). Editing efficiency was calculated as shown in Equation 1.

(1) $$\begin{eqnarray*} && {\rm{Editing\ efficiency }} \nonumber \\ && = \frac{{{\rm{Greyscale }}\left( {{\rm{edited}}} \right)/{\rm{length }}\left( {{\rm{bps}}} \right)\left( {{\rm{edited}}} \right)}}{{{\rm{Greyscale }}\left( {{\rm{edited}}} \right){\rm{ }}/{\rm{length }}\left( {{\rm{edited}}} \right){\rm{ }} + {\rm{ Greyscale }}\left( {{\rm{non}} - {\rm{edited}}} \right)/{\rm{length }}\left( {{\rm{non}} - {\rm{edited}}} \right)}}100\%\nonumber\\ \end{eqnarray*}$$

### Detecting the deletion or replacement efficiency via capillary electrophoresis

For capillary electrophoresis (Supplementary Figure S5.), the amplicons were subjected to Bio-Fragment Analyzer (Bioptic, Qsep1, C100001) with an S2 Cartridge. The DNA size marker (20–5000-bp) from Bioptic was used as internal control. Data were analyzed by Q-Analyzer software to calculate the peak area. Editing efficiency was calculated as shown in Equation 2.

(2) $$\begin{eqnarray*} && {\rm{Editing\ efficiency }} \nonumber \\ && = \frac{{{\rm{peak\ area }}\left( {{\rm{edited}}} \right)/{\rm{length }}\left( {{\rm{bps}}} \right)\left( {{\rm{edited}}} \right)}}{{{\rm{peak\ area }}\left( {{\rm{edited}}} \right){\rm{ }}/{\rm{length }}\left( {{\rm{edited}}} \right){\rm{ }} + {\rm{ peak\ area }}\left( {{\rm{non}} - {\rm{edited}}} \right)/{\rm{length }}\left( {{\rm{non}} - {\rm{edited}}} \right)}}100\%\nonumber\\ \end{eqnarray*}$$

### Detecting the LoxP insertion efficiency via monoclonal analysis

For analyzing double LoxP insertions, the putative amplicons containing LoxP were purified with GeneJET Gel Extraction Kit (Thermo scientific) and ligated into a blunt-end vector using the pESI-blunt kit (YEASEN). DH5α-competent cells were then transformed with the ligation product. Colonies containing perfect double-LoxP insertions were recognized as accurate editing, and the ones containing double-LoxP insertions but harboring indels were recognized as inaccurate editing.

### Targeted deep sequencing and data analysis

Genomic DNA (gDNA) was extracted 72 h after transfection and subjected to PCR analysis using Phanta^®^ Max Super-Fidelity DNA Polymerase (Vazyme). The PCR reaction included 1 μl of cell lysate, and 0.2 μM of forward and reverse primers in a final reaction volume of 30 μl. Genomic regions of interest were amplified by PCR with primers flanked with different barcodes (Supplementary Table S4). PCR reactions were performed as follows: 95°C for 3 min, then 35 cycles of (95°C for 15 s, 60°C for 15 s, and 72°C for 10 s), followed by a final 72°C extension for 10 min. The PCR products were purified with GeneJET Gel Extraction Kit (Thermo scientific) and quantified with NanoDrop (Thermo Fisher). Samples were sequenced commercially using the Illumina Novaseq6000 platform (150 bp, paired-end, Personal Biotechnology, Shanghai, China). A custom python script provided in Supplementary Note 1 was used to analyze and quantify the efficiency of the desired edits and indels produced by Bi-PE, PE3 and WT-Cas9. Substitution and indel frequencies were quantified as the percentage of total sequencing reads, and the threshold of editing activity was set to above 0.01%.

### Statistical analyses

GraphPad Prism 8 software was used to analyze the data. All values are presented as mean ± s.d. of at least two independent biological replicates. Differences among groups were tested using two-tailed Student's t-tests. A *P* value < 0.05 was considered statistically significant. ***P < 0.001, **P < 0.01, *P < 0.05.

## RESULTS

### Position of the nick affects the efficiency of PE3 in large fragment deletion.

Previous studies have characterized the performance of PE3 in targeted small fragment deletions, while large fragment deletion remains a big challenge. To test the ability of PE3 in targeted deleting relatively large genomic fragment, ∼100–1000 bp, we designed a set of four pegRNAs targeting *HEK3* loci to delete 198-bp, 372-bp, 530-bp and 654-bp fragment, respectively, all of which share a common spacer and prime binding site (PBS). An sgRNA that breaks the un-edited strand at + 90 was chosen as nick sgRNA (Supplementary Figure S1a). It was noteworthy that the spacers of the two sgRNAs and the PBS of the pegRNA had been demonstrated to be functional ((6) and data not shown). However, transfection of these individual set of pegRNA and sgRNA, together with PE2, into HEK293T cells did not result in any detectable desired genomic deletion (Supplementary Figure S1b).

**Figure 1.**
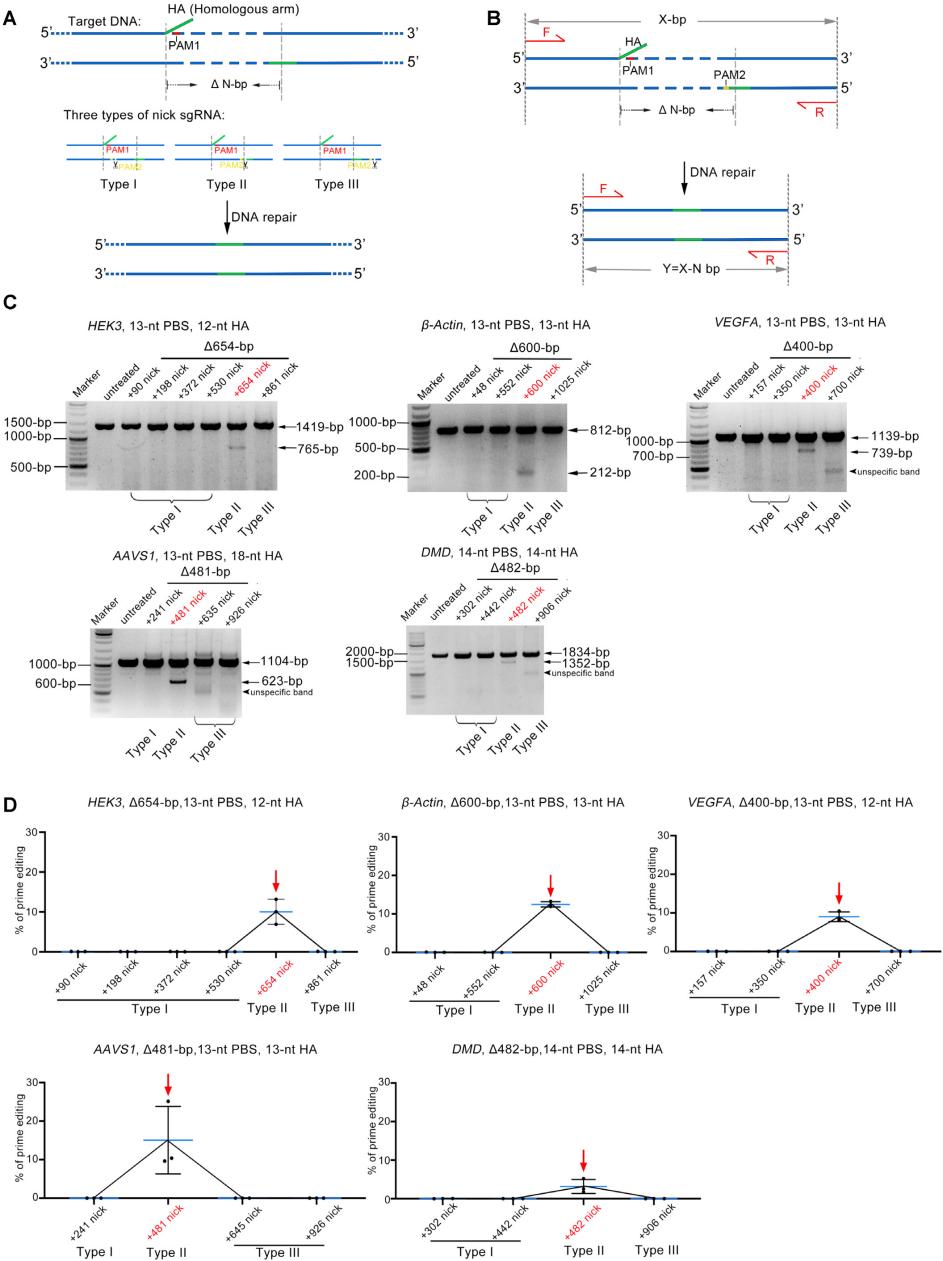
Positioning nick sgRNA nearby the HA region improve PE3 mediated deletion of large DNA fragment. (**A**) Schematic diagram showing the design of five types of nicks sgRNAs (Types I, II and III) and the putative editing process of PE3 mediated deletion of large DNA fragment. Homologous arm (HA, homologous to the DNA sequence downstream of the fragment to be deleted) and its complementary region were shown in green. PAMs of pegRNA and nick sgRNA were shown in red and yellow respectively. (**B**) Schematic diagram showing the detection of targeted deletion by PCR analysis. Paired primers were designed to amplify the targeted deletion and its flanking sequences (fragment without deletion = *X*; fragment with deletion = *Y* (*Y* = *X* – *N*). (**C**) Representative agarose gel electrophoresis detecting the presence of targeted deletion. Note that only type II nicks generated targeted deletions in all three loci studied. (**D**) Adobe Photoshop CC (2019) quantifying the efficiencies of the targeted deletions. Values and error bars reflect mean ± s.d. of *n* = 3 independent biological replicates. Data of each replicate were shown in Supplementary Table S5.

In prime editing system, the newly reverse transcribed ssDNA was thought to invade into the downstream double strand DNA through homologous DNA pairing and ssDNA invasion, thereby inducing DNA repair mechanisms to introduce reverse transcribed ssDNA into the genome (6,18,19). We reasoned that the failure of those PE3s was partially due to inefficient ssDNA invasion and that repositioning the nicks to the region pairing with HA should facilitate the invasion and improve targeted deletion. To test this hypothesis, we designed three types of nicks, which were located inside (Type I), adjacent to the 3′ end of (Type II) or downstream (Type III) of the fragment to be knocked out (Figure 1A, B). The pegRNA was designed to delete a 654-bp fragment from *HEK3* locus and totally six nicks were designed, including 4 Type I, 1 Type II and 1 Type III nick sgRNAs (Figure 1C). Before PE experiments, all the nick sgRNAs were validated to be functional by wild-type Cas9 in HEK293T cells, leading to the formation of indels within target positions of each sgRNAs (Supplementary Figure S2). However, PE experiments revealed that only Type II nicks produced detectable targeted deletion of the 654-bp fragment in HEK293T cells, as evidenced by agarose gel analysis of the amplicons flanking the target deletion (Figure 1B, C and Supplementary Figure S3). This phenomenon was further confirmed by four additional target editing, in which a 600-bp fragment in *β-Actin* locus, a 400-bp fragment in *VEGFA* locus, a 481-bp in *AAVS1* locus and a 482-bp in *DMD* locus could only be deleted by PE3 with Type II nick (Figure 1C, D and Supplementary Figure S3). Moreover, this phenomenon was also demonstrated to be true in additional cell types, including HeLa (epithelial cell) and K562 (myelogenous leukemia) cells (Supplementary Figure S4). Together, these results supported a mode involving ssDNA invasion during the repair of PE induced lesion and suggested that positioning the nick of the non-edited strand to the homologous region achieved large fragment deletion.

### Bi-PE in large fragment deletion

**Figure 2.**
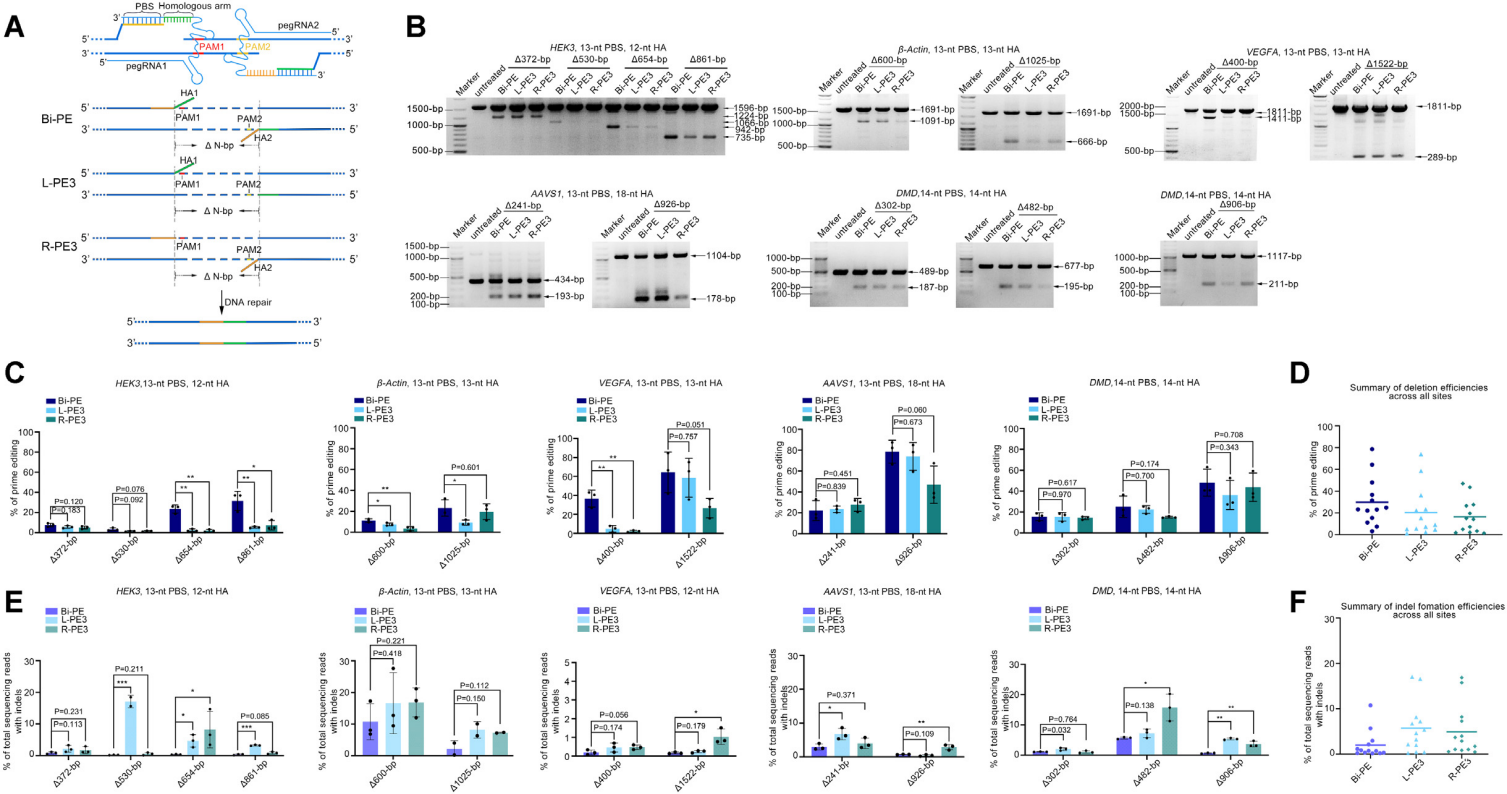
Bi-PE strategy significantly increased the efficiency of large fragment deletion. (**A**) Schematic diagram showing the design and the putative editing process of Bi-PE strategy. In Bi-PE strategy, both sgRNAs were designed to be pegRNAs, each of which contained a HA homologous to the sequences proximally flanking the deleted fragment. PE3 containing up-stream pegRNA and down-stream nick sgRNA was designated L-PE3 and vice versa was R-PE3. HA in up-stream pegRNA and its homologous region were shown in green, and the corresponding ones in downstream pegRNA were shown in yellow. (**B**) Agarose gel analysis of the presence of targeted deletions on indicated loci. (**C**) Quantification of the efficiency of targeted deletions using Adobe Photoshop CC (2019). Values and error bars reflect mean ± s.d. of *n* = 3 independent biological replicates. Data of each replicate were shown in Supplementary Table S5. (**D**) Summary of deletion efficiencies across 5 endogenous sites in HEK293T cells. The mean of all individual values of *n* = 3 independent biological replicates was plotted. (**E**) HTS analysis of the frequencies of undesired Indels. The fragments containing deletions were gel purified and amplified by HTS primers. Then the products were subjected to HTS to analyze undesired Indels. All alleles observed with frequency ≥0.01% were shown. Values and error bars reflect mean ± s.d. of *n* ≥ 2 independent biological replicates. (**F**) Summary of indel frequencies across five endogenous sites in HEK293T cells. The mean of all individual values of *n* ≥ 2 independent biological replicates was plotted.

Inspired by above findings, we hypothesized that engineering the nick sgRNA to pegRNA would allow bi-directional priming, thereby doubling the efficiency of large fragment deletion. Henceforth, the design of bi-directional priming was referred to as Bi-PE. PE3 using upstream sgRNA as pegRNA was referred to as left PE3 (L-PE3) and the one using downstream sgRNA as pegRNA was referred to as right PE3 (R-PE3) (Figure 2A). To compare the efficiency of Bi-PE and PE3, we designed a panel of Bi-PEs and corresponding PE3s targeting *HEK3* loci to delete 372-bp, 530-bp, 654-bp and 861-bp fragment, respectively and determined the presence of targeted deletions by agarose gel analysis of the amplicons flanking the target region (Figure 2B). We observed a significantly improvement of the deletions by Bi-PE as compared to PE3 in two out of four deletions in HEK293T cells (654-bp and 861-bp, Figure 2C). In the deletion of 861-bp, the editing efficiency of Bi-PE achieved ∼31.5%, which was 5 times and 3 times higher than that of L-PE3 and R-PE3 respectively (Bi-PE: L-PE3: R-PE3 = 31.5%: 5.3%: 7.3%). In the deletion of 654-bp, the efficiency of Bi-PE was 9 and 12 times higher than that of L-PE3 and R-PE3 respectively (Bi-PE: L-PE3: R-PE3 = 23.4%: 2.4%: 1.8%). The efficiency of Bi-PE in deleting 530-bp was 1.5 and 1.4 times higher than that of L-PE3 and R-PE3 respectively (Bi-PE: L-PE3: R-PE3 = 3.3%: 1.3%: 1.4%). And the efficiency of Bi-PE in deleting 372-bp was 0.36 and 0.52 times higher than that of L-PE3 and R-PE3 respectively (Bi-PE: L-PE3: R-PE3 = 7.6%: 5.6%: 5.0%) (Figure 2C). To confirm the accuracy of above data determined by agarose gel analysis, we conducted capillary electrophoresis analysis. As shown in Supplementary Figure S5, results of capillary electrophoresis were consistent with those obtained from agarose gel analysis. Overall, Bi-PE exhibited a higher activity than either L-PE3 and R-PE3 (Figure 2D).

Because Bi-PE produced double nicks in the target region, it is possible that these nicks would induce fragment deletion via NHEJ or HDR (the 3′ end of pegRNA serving as donor template) pathways. To test such possibility, we coupled paired pegRNAs or their corresponding sgRNAs to Cas9 nickase. However, neither combination produced detectable targeted deletions (Supplementary Figure S6a), suggesting that these targeted deletions were PE-specific.

To test if the improvement of Bi-PE in deleting large genomic fragment was general, we included additional nine targeted deletions in four different loci, *β-Actin, VEGFA, AAVS1* and *DMD* in HEK293T cells (Figure 2B and C). In *β-Actin* locus, the average efficiency of Bi-PE in deleting 600-bp fragment was ∼11.3%, which was 0.53 times higher than that of L-PE3 and 2.3 times higher than that of R-PE3 (Bi-PE: L-PE3: R-PE3 = 11.3%: 7.4%: 3.4%). In the same locus, Bi-PE had an average efficiency of 23.2% in deleting 1025-bp fragment, which was 1.5 times higher than L-PE3 and 0.39 times higher than R-PE3 (Bi-PE:L-PE3:R-PE3 = 23.2%:9.2%:16.7%). Similar improvement of Bi-PE over PE3 was also observed in *VEGFA* locus, in which the efficiency of Bi-PE in deleting 400-bp fragment was 6 times and 16 times higher than that of L-PE3 and R-PE3 respectively (Bi-PE:L-PE3:R-PE3 = 36.7%:5.3%:2.2%). In the deletion of 1522-bp fragment, Bi-PE had an average editing efficiency of 64.4%, an increase of 0.1 and 1.4 times, respectively, over L-PE3 and R-PE3 (Bi-PE:L-PE3:R-PE3 = 64.4%:58.7%:26.6%). In the rest five targeted deletions, Bi-PE outperformed both L-PE3 and R-PE3 in 3 deletions (926-bp in *AAVS1* locus and 482-bp and 906-bp in *DMD* locus) in terms of deletion efficiency. In the deletion of 302-bp in *DMD* locus, Bi-PE showed similar efficiency to both PE3s. And only in one deletion (241-bp in *AAVS1*), Bi-PE was less efficient than both PE3s (Figure 2C). In summary, Bi-PE showed a higher editing efficiency in large fragment deletion than PE3 in 11 out of 13 deletion events (Figure 2C).

Then we tested if the efficiency of Bi-PE could be further improved by optimizing the HA length. We varied the length of HA from 8-bp to 50-bp. The results showed that HA length did influence the efficiency of deletion, but the effect was case-dependent and not strictly predictive (Supplementary Figure S7). Therefore, we recommend optimizing the length of HA when editing efficiency of Bi-PE is valued.

Next, we tested if Bi-PE also outperformed PE3 in large fragment deletion in other cell types, including K562, HeLa and B16 (mouse melanoma cell) cells. We found that the efficiency of Bi-PE was generally higher than that of PE3 in these cells. Strikingly, PE3 did not produce any detectable deletions in three events (600-bp in *β-Actin* locus in K562 cell, and 254-bp and 336-bp in *Hoxd* locus in B16 cell). By contrast, Bi-PE produced obvious deletions in the same events (Supplementary Figure S8). Together, these results demonstrated a significant improvement of Bi-PE over PE3 in targeted deletion of large DNA fragment (Figure 2B–D).

It has been observed that PE3 produced considerable level of indels in generating point mutations or small fragment insertions/deletions, which was likely due to PE3 producing two nicks in each strand of its target loci (6). An examination of the HTS data revealed that PE3 also produced undesired indels in alleles with aimed large fragment deletions, leading to inaccurate edits (Figure 2E and Supplementary Figure S9). For example, in *HEK3* loci, L-PE3 mediated deletion of 654-bp fragment produced 4.7% undesired indels and R-PE3 produced 8.2% indels. By contrast, Bi-PE mediated same deletion only produced 0.35% indels. In the same loci, when deleting 372-bp fragment, L-PE3 and R-PE3 produced 2.2% and 1.74% indels, respectively, while Bi-PE only produced 0.85% indels (Figure 2E). This phenomenon was further confirmed by additional 11 targeted deletions (Figure 2E). On average, Bi-PE produced more than 2 times lower indels than PE3 (Figure 2F).

Finally, we sought to compare Bi-PE to wildtype Cas9 coupled with paired sgRNAs in targeted deletion since the latter has been shown to be efficient in such editing. We designed 6 targeted deletions and found that although wildtype Cas9 with paired sgRNAs achieved higher deletion efficiency compared to Bi-PE (on average, wildtype Cas9:Bi-PE = 68.3% versus 38.1%) (Supplementary Figure S6b), wildtype Cas9 produced deletions were accompanied by significantly higher levels of un-intended indels compared to Bi-PE (on average, wildtype Cas9:Bi-PE = 17.2% versus 5.3%) (Supplementary Figure S6c).

### Genomic fragment replacement by Bi-PE

**Figure 3.**
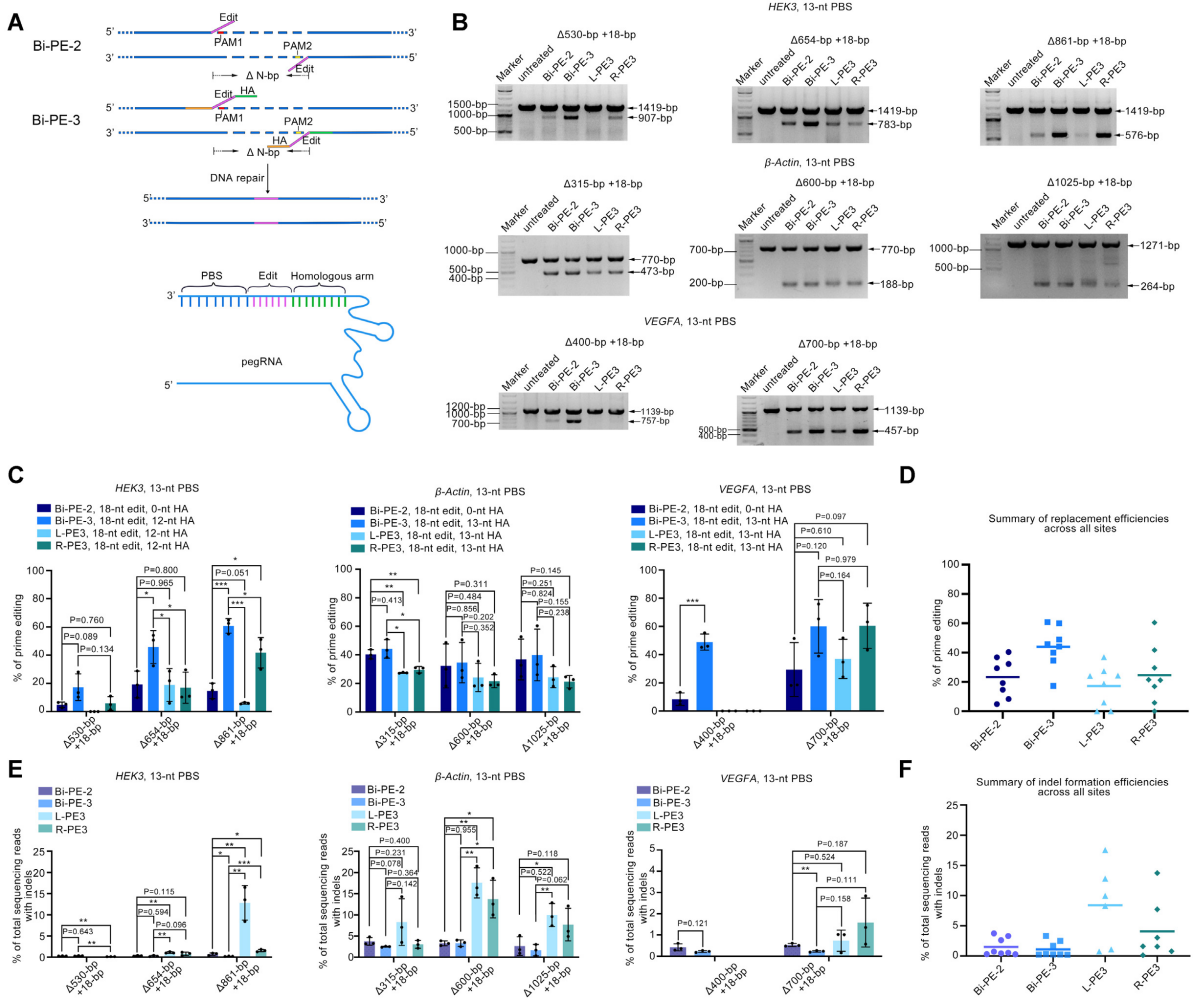
Genomic fragment replacement by Bi-PE. (**A**) Schematic diagram showing the design and the putative editing process of Bi-PE-2 and Bi-PE-3 strategies. Bi-PE-2 generated a pair of 3′ flaps containing only the insertion fragments, and Bi-PE-3 generated 3′ flaps containing both insertions and HAs humongous to the sequences flanking the deletion. (**B**) Gel analysis of the indicated alleles harboring targeted deletions. (**C**) Quantification of the efficiency of targeted deletions and insertions using Adobe Photoshop CC (2019). Values and error bars reflect mean ± s.d. of *n* = 3 independent biological replicates. Data of each replicate were shown in Supplementary Table S5. (**D**) Summary of replacement efficiencies across three endogenous sites in HEK293T cells. The mean of all individual values of *n* = 3 independent biological replicates was plotted. (**E**) HTS analysis of the frequencies of undesired Indels. The fragments containing deletions were gel purified and amplified by HTS primers. Then the products were subjected to HTS to analyze undesired Indels. All alleles observed with frequency ≥0.01% were shown. Values and error bars reflect mean ± s.d. of *n* = 3 independent biological replicates. (**F**) Summary of indel frequencies across three endogenous sites in HEK293T cells. The mean of all individual values of *n* = 3 independent biological replicates was plotted.

Above results suggested that Bi-PE significantly improved the ability of PE system in targeted deleting large DNA fragment as compared to PE3. Next, to further explore its versatility, we examined if Bi-PE system can insert a small fragment while deleting large fragments from the target loci (fragment replacement). As shown in Figure 3A, we designed two Bi-PE strategies, Bi-PE-2 and Bi-PE-3, both of which encoded the same replacement sequences (the edits). The pegRNAs of Bi-PE-3 contain both the edit and the HA that is homologous to the DNA sequence downstream of the deletion, while the ones of Bi-PE-2 contain only the edits (Figure 3A). Bi-PE-2 strategy was designed to test whether HA is necessary for the targeted replacement.

To assess the editing efficiency of the two Bi-PE and PE3 strategies, we tested their performance in 8 replacement events located on three different loci in the HEK293T cells (Figure 3B). On *HEK3* locus, the efficiency of Bi-PE-2 in the deletion of 530-bp fragment while simultaneous insertion of 18-bp fragment (–530 + 18) was lower than that of Bi-PE-3 (4.8% versus 17.3%). While Bi-PE-2 showed significantly lower activity than Bi-PE-3 in the deletion of 654-bp (19.3% versus 45.8%) or 861-bp fragments (14.7% versus 60.8%) (Figure 3C). On other editing, Bi-PE-2 also showed lower activity than Bi-PE-3 (*β-Actin* –315 + 18, Bi-PE-2: Bi-PE-3 = 40.3%: 44.1%; *β-Actin* –600 + 18, Bi-PE-2: Bi-PE-3 = 32.3%: 34.6%; *β-Actin* –1025 + 18, Bi-PE-2:Bi-PE-3 = 36.8%:39.9%; *VEGFA* –400 + 18, Bi-PE-2:Bi-PE-3 = 8.4%: 48.8%; *VEGFA* –700 + 18, Bi-PE-2:Bi-PE-3 = 29.4%:60.0%) (Figure 3C). As a control, Cas9 nickase (H840A) coupled with pegRNAs did not produce detectable deletion and replacement, ruling out the possibility that pegRNAs acted as templates for HDR repair to induce such editing events (Supplementary Figure S10). Collectively, these observations demonstrated Bi-PE-3 strategy was more efficient than Bi-PE-2 in fragment replacement (Figure 3D), suggesting that although HA was not necessary for the replacement, but its presence guaranteed the editing efficiency (Figure 3B–D). Then we focused on Bi-PE-3 to test if its efficiency could be further improved by optimizing the HA length. We varied the length of HA from 8-bp to 50-bp. Similar to the results obtained from fragment deletion, we found that the effect of HA length was case-dependent and not strictly predictive (Supplementary Figure S11).

Next, we compared the efficiency of the two Bi-PE strategies to that of PE3s. We found that Bi-PE-3 outperformed both sides of PE3s in seven out of eight editing events, while Bi-PE-2 only outperformed both PE3s in four out of eight events (Figure 3B and C). And on average, Bi-PE-3 achieved a replacement efficiency of 43.9%, which is the highest one among the four strategies (Bi-PE-2:Bi-PE-3:L-PE3:R-PE3 = 23.3%:43.9%:17.2%: 24.7%) (Figure 3D).

Then we focused on Bi-PE strategy and tested whether the editing efficiency was correlated with the length of replacement sequence. We varied the replacement length from ∼10-bp to ∼100-bp. The results showed the editing efficiency seemed not decrease as the length of replacement increase. On *VEGFA* and *β-Actin* loci, the efficiencies of ∼100-bp replacement were even slightly higher than those of ∼10-bp replacement (Supplementary Figure S12).

In addition to editing efficiency, we also observed that Bi-PE-2 and Bi-PE-3 produced a lower level of indel than PE3 (Supplementary Figure S13). The most prominent example is the deletion of 861-bp fragments at the *HEK3* locus, where Bi-PE-3 produced about 0.19% indels, while Bi-PE-2 produced about 0.72% indels, L-PE3 produced about 12.8% indels, and R-PE3 produced about 1.6% indels (Figure 3E). Overall, Bi-PE-2 and Bi-PE-3 produced less indel than PE3 in most editing events (six out of seven events) (Figure 3F). Together, these results suggested Bi-PE-3 strategy, but Bi-PE-2 outperformed PE3 in both efficiency and accuracy in fragment replacement.

### Multiplex base conversions by Bi-PE

After demonstrating that Bi-PE is more efficient than PE3 in targeted manipulations of large fragments, we next evaluate whether Bi-PE can also outperform PE3 in base conversions. We compared the performance of Bi-PE and PE3 in the edition of single base conversion in HEK293T cells (Supplementary Figure S14a). On *FANCF* locus (+5 G·C to T·A), the average editing efficiency of Bi-PE-2 and Bi-PE-3 was 9.7% and 12.7%, respectively, while the efficiency of L-PE3 and R-PE3 was 20.7% and 2% respectively (Supplementary Figure S14b). On *β-Actin* locus (+6 G·C to T·A), Bi-PE-2 and Bi-PE-3 achieved an average efficiency of 21% and 20.5% respectively. And L-PE3 and R-PE3 achieved an average editing efficiency of 27.7% and 3%, respectively (Supplementary Figure S14b). These data suggested that Bi-PE did not outperform PE3 in base conversion editing.

Next, we compared the performance of Bi-PE and PE3 on simultaneous conversion of two bases. The pegRNAs of each direction (L- and R-pegRNA) were designed to convert bases within each corresponding PAMs (Supplementary Figure S14c). On *FANCF* locus (+5 G·C to T·A, +44 C·G to A·T), Bi-PE-2 and Bi-PE-3 had average editing efficiency of 7.2% and 20.2% respectively. And the average editing efficiencies of L-PE3 and R-PE3 were 13.2% and 4% respectively. On *β-Actin* locus (+6 G·C to T·A, +44 C·G to A·T), the average editing efficiencies of Bi-PE-2 and Bi-PE-3 were 18.8% and 16% respectively, while the ones of L-PE3 and R-PE3 were 13% and 8.8%, respectively (Supplementary Figure S14d). These comparisons collectively suggested that Bi-PE-3 strategy was generally more efficient than PE3 in simultaneous conversion of two bases. Overall, the improvement of Bi-PE-3 over L- or R-PE3 ranged from 0.8 to 4.1 times (Supplementary Figure S14d).

**Figure 4.**
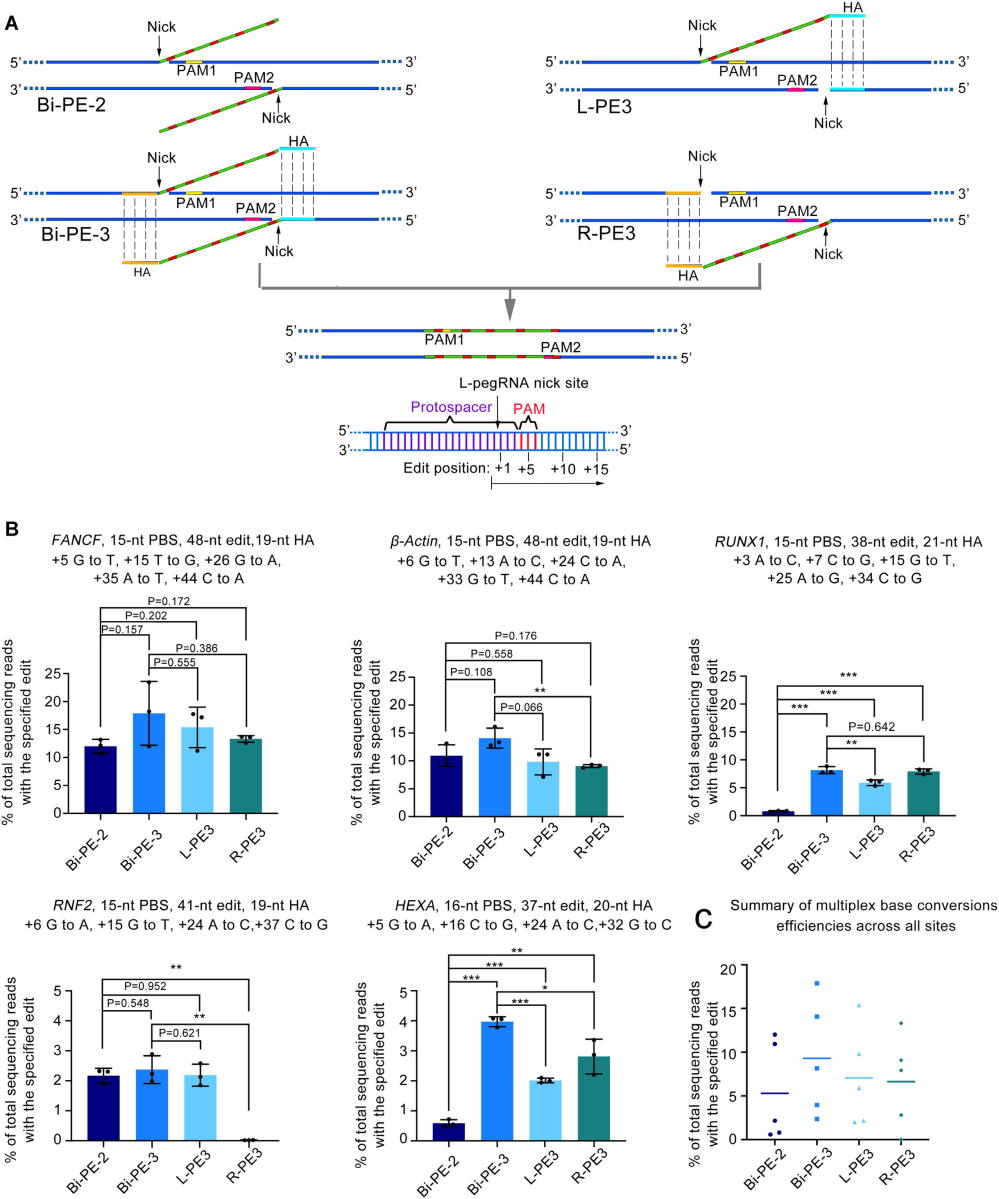
Simultaneous conversion of multiple bases by Bi-PE. (**A**) Schematic diagram showing the design and the putative editing process of Bi-PE-2, Bi-PE-3, L-and R-PE3 strategies. Bi-PE-2 generates a pair of 3′flaps containing only the targeted edits, and Bi-PE-3 generates 3′ flaps containing both edits and HAs humongous to the sequences flanking the editing region. (**B**) HTS quantifying the frequencies of alleles containing simultaneous conversions of multiple bases. All alleles observed with frequency ≥0.01% were shown. Values and error bars reflect mean ± s.d. of *n* = 3 independent biological replicates. Data of each replicate were shown in Supplementary Table S5. (**C**) Summary of multiple bases conversion efficiencies across five endogenous sites in HEK293T cells. The mean of all individual values of *n* = 3 independent biological replicates was plotted.

We then examined if Bi-PE was also efficient in multiplex base conversions. We designed pegRNAs of each direction to simultaneously introduce 5-base pairs conversions on *FANCF, β-Actin* and *RUNX1* loci, and 4-bp conversions on *RNF2* and *HEXA* loci (Figure 4A and B). We observed robust multiplex base conversions across all editing strategies. And similar to the findings in two-base conversions, we found that Bi-PE-3 but not Bi-PE-2 strategy was more efficient than either L- or R-PE3 in multiplex base conversions (Bi-PE-2:Bi-PE-3:L-PE3:R-PE3 = 5.3%:9.3%:7.1%:6.6%) (Figure 4B and C). Noteworthy, in the editing products, there were a small fraction containing imperfect conversions (at least one target bases not being converted). The ratio of imperfect conversion ranged from 0.05% to 13% (Supplementary Figure S15).

### Double fragment insertion by Bi-PE

**Figure 5.**
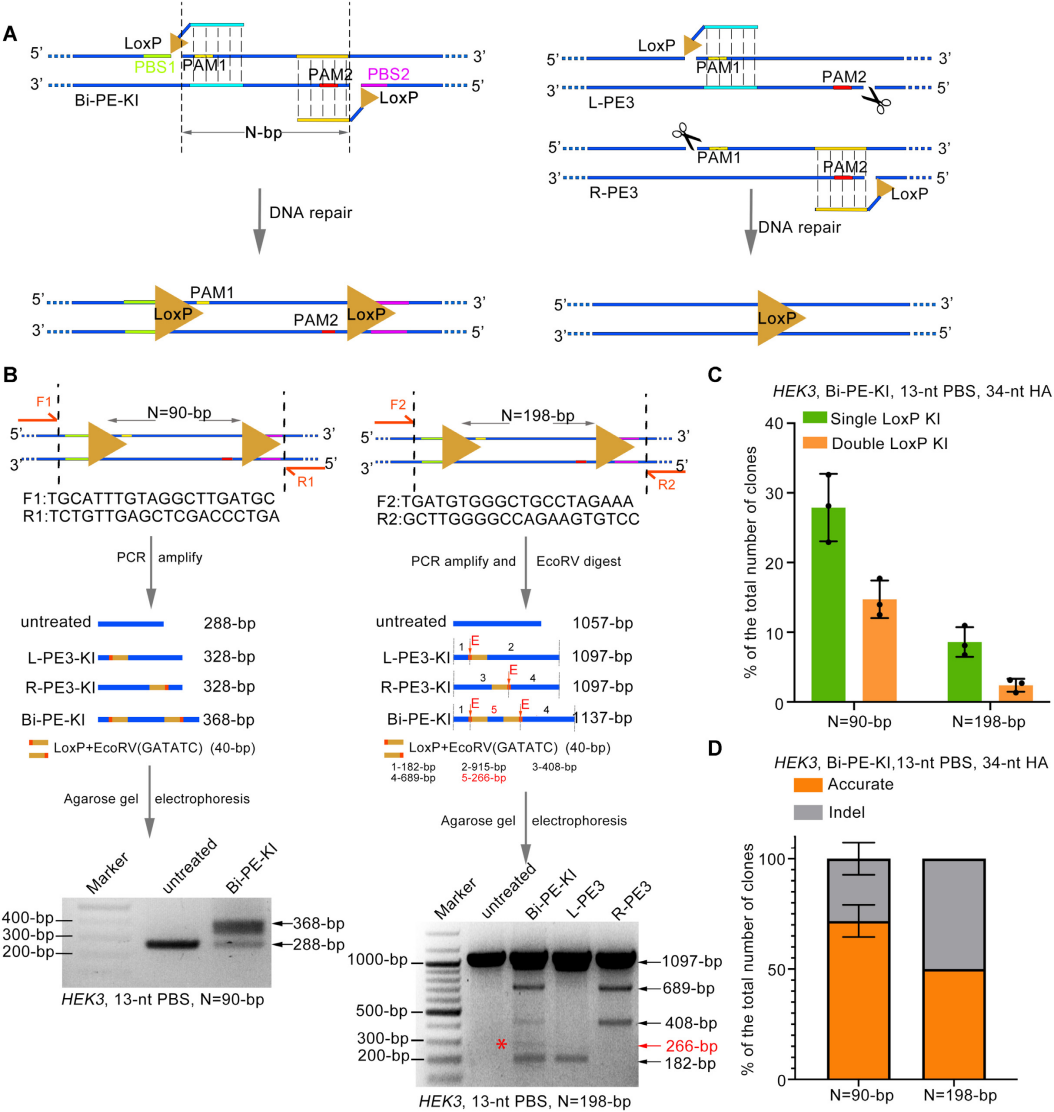
Simultaneous insertion of paired LoxP sites into the same allele by Bi-PE. (**A**) Schematic diagram showing the design and the putative editing process of Bi-PE mediated insertion of paired LoxP sites. (**B**) PCR analysis of the targeted insertions of LoxP sites. Upper and middle panels were schematic diagrams showing the designs of the PCR primers and the putative outcomes of single or double LoxP insertions. Lower panels were agarose gels analysis of the PCR products with or without EcoRV digestion. (**C** and **D**) Quantifying the efficiency and purity of the targeted insertion of double LoxP sites through single colony analysis. PCR products from (**B**) were cloned into pESI-blunt, and the resulting single colonies were analyzed by PCR and Sanger sequencing. Colonies containing perfect double-LoxP insertions were recognized as accurate editing and the ones containing double-LoxP insertions but harboring indels were inaccurate. Detailed information of the indels were shown in Supplementary Figures S12 and S13.

Previous study had demonstrated that PE3 strategy was able to insert single LoxP site into the genome. However, the most widely application of Cre/LoxP system, conditional loss- or gain-of-function experiments, require the insertion of paired LoxP sites. To test if Bi-PE strategy could achieve targeted insertion of paired LoxP sites in the same allele, we design a pair of pegRNAs aiming to insert paired LoxP sites with same orientation and flanking a 90-bp segment into the *HEK3* locus (Figure 5A). We transfected those pegRNAs together with PE2 into HEK293T cells and determined the presence of LoxP insertions by PCR. For the PCR reaction, we designed paired primers flanking outside the PBS to avoid amplifying randomly integrated LoxP. As shown in Figure 5B, we did detect a band corresponding to the size of double LoxP insertions. To better quantify the efficiency of double LoxP insertions, we cloned the PCR products into plasmids and performed single colony analysis (Supplementary Figure S16a). The analysis revealed that the efficiency of double LoxP insertions was about 13.2% (Figure 5C and Supplementary Figure S16b). Sanger sequencing of these colonies harboring LoxP sites confirmed that these events were actually targeted but not random insertion (Supplementary Figure S16c). Among the alleles harboring double LoxP sites, 72% were accurate and the rest alleles were found to contain fragment deletions between the LoxP sites (Figure 5D, Supplementary Figure S16c and Supplementary Figure S16d). Then we tried to lengthen the segment flanked between LoxP sites to 198-bp (Figure 5B). To facilitate the detection of LoxP insertion, we added EcoRV sequences to each LoxP sites, so that the presence of the insertions could be detected by EcoRV digestion. As shown in Figure 5B, we observed alleles containing single or double EcoRV sites, indicating single or double LoxP insertions respectively. However, the level of the insertions was significantly lower than that observed in 90-bp segment. Single colony analysis revealed that the efficiency of double LoxP sites was 2.9%, in which 52% were accurate (Figure 5C, D and Supplementary Figure S17). When we lengthened the segment to 372-bp, we did not detect obvious LoxP insertions (data not shown). Together, these results suggested that Bi-PE strategy was able to achieve double fragment insertion, but its efficiency attenuated with the lengthening of the segment in-between the insertions.

## DISCUSSION

In this work, we showed that positioning the nick sgRNA nearby the HA region of pegRNA enabled targeted deletions of large DNA fragment, which is possibly due to an enhancement of the ssDNA invasion of the HA. Based on this finding, we designed a bi-directional prime editing strategy, Bi-PE, in which, both sgRNAs were engineered to be pegRNAs encoding same edits. We showed that our Bi-PE strategy worked efficiently in the targeted deletions of large DNA fragment, ranging from hundreds to thousands of base pairs. Meanwhile, Bi-PE strategy could efficiently introduce a small fragment of tens to one hundred of base pairs into the deleting sites. Very recently, two independent labs developed similar bi-directional prime editing systems to ours (20,21), both of which observed efficient targeted deletion and replacement. In addition to these types of editing, our work showed that Bi-PE strategy also improved the efficiency of simultaneous conversion of multiple bases within same sites and enabled the insertion of two LoxP sites into the same allele.

The action of PE system introduced a newly reverse transcribed ssDNA to the nick of the edited strand, which was then integrated into the genome possibly through DNA repair or replication related mechanisms. Although there was not any evidence about how the ssDNA was integrated, the process could be intimated from several natural processes, such as homology directed DSB repairs and Okazaki fragments maturation during DNA replication (22–24). A possible mode would be that the newly reverse transcribed ssDNA replaced the original strand through ssDNA searching and invasion, leading to the nick migration and a 5′ flap. The flap could be cleaved by structure-specific endonucleases such as FEN1, DNA2 and Exo1 preferring 5′ flap to 3′ flap, and the resulting 5′ end was then ligated to 3′ end of the newly transcript ssDNA (22,25–28). Another possible mode would be that the 5′ end was resected, possibly through 5′ exonucleases, to generate an ssDNA region that serve as a complementary strand to the newly reverse transcribed ssDNA, following by base pairing of them and the ligation of the ssDNA to the resected 5′ end (29–32). Our observation that positioning the nick sgRNA adjacent to the HA region of pegRNA improved PE3 mediated targeted deletions of large DNA fragment was supportive to the former mode involving homologous searching and ssDNA invasion, since the latter mode was unlikely affected by the positions of the nick sgRNA.

Interestingly, in our conditions, Bi-PE strategy did not improve the efficiency of single base conversion as compared to PE3. We speculate that bi-directional priming generally has two types of effects on the efficiency of PE. (i) Promotes editing efficiency by doubling the chances of priming and enhancing the homology searching process. However, the extend of promotion was editing-type dependent, with single point conversion being smallest. (ii) Attenuates editing efficiency because of the inhibitory effect resulted from pegRNA circularization. A previous study found that the PBS sequence of pegRNA could anneal to the spacer, leading to circulation of pegRNA, which impaired the function of pegRNA even as a nick sgRNA (33). Thus, the dysfunction of pegRNA would reduce the level of nicks as compared to PE3, generally attenuating the editing efficiency. In multisite conversion and fragment deletion, such inhibitory effect can be exceeded by bi-directional priming, resulting in an increased efficiency of Bi-PE compared to PE3. However, in single base conversion, the promoting effect of bi-directional priming is not enough to exceed the inhibitory effect of pegRNA dysfunction. A recent publication showed that bi-directional prime editing increased the efficiency of single base conversion and small fragment deletions or insertions (no more than 2 bp) in plants (34). However, that publication compared the efficiency of bi-directional prime editing to that of PE2 lacking a nick in the un-edited strand. Investigations of PE in plants revealed that PE2 has similar editing efficiency to PE3 (35–40), which was quite distinct from the observations in mammalian cells showing that PE3 outperformed PE2 in the majority of target sites (6). The difference of the editing features of PE systems (PE2, PE3 and Bi-PE) between mammals and plants might reflect differences in the DNA damage response and repair mechanisms between these two kingdoms (30,41–47). Noteworthy, frequencies of fragment deletions were determined by PCR and agarose gel analysis in this study, which potentially overestimated the editing efficiencies because of the amplification bias favoring smaller fragment. Actually, Choi *et al.* have performed linear amplification using Unique Molecular Identifiers (UMIs) to reduce the potential bias and observed that frequency determined by UMIs was slightly lower than that determined by PCR (20).

Importantly, in addition to increasing the efficiency, Bi-PE strategy produced less undesired indels than PE3. The generation of indels during prime editing was likely due to a pair of nicks were simultaneously introduced into each of the double strands, which was possibly recognized as double strand breaks by chance, leading to the activation of NHEJ pathway and the generation of indels (48–50). In fact, harnessing Cas9 nickase to produce paired nicks was frequently used to produce indels during gene disruption studies, aiming to reduce Cas9 dependent off-target effects (51–54). Compared to the reported levels of indel generated by Cas9 nickase, the ones generated by prime editing were much lower, indicating that the extended sequence of the nicks inhibited indel formation. Therefore, theoretically, the chances of indels would be further attenuated when both nicks were extended.

The observation that Bi-PE could produce double fragment insertion demonstrated its versatility in genome manipulating. In addition to insert LoxP sites, Bi-PE could be designed to insert double or multiple cis elements within the regulatory region of the genome. Specially, many enhancers were known to require synergistic actions of double cis elements spaced by fragments of tens of base pairs (55–57). Bi-PE strategy might be helpful for the investigation of such enhancers. The fact that the efficiency of double fragment insertion decreased significantly with the increasing of the length of the segment suggested that optimization was required when designing such insertions. Recently, the Twin-PE strategy reported by Anzalone et al used bi-directional pegRNAs to insert small fragment (21). The overlapped regions of Twin-PE pegRNAs do not cover the nick site or its 5′ flanking region. Such design is similar to our Bi-PE-2 and reduces the RT-template length as compared Bi-PE-3, in which the overlapped regions cover 5′ flanking region of nick site, therefore Twin-PE strategy should be useful in double-LoxP insertion.

## DATA AVAILABILITY

All data supporting the findings of this study are available in the article and its supplementary figures and tables or can be obtained from the corresponding authors upon reasonable request. Deep-sequencing data are available under BioProject ID PRJNA809555.

## Supplementary Material

gkac506_Supplemental_Files
