# Supplementary material for: Bi-PE: bi-directional priming improves CRISPR/Cas9 prime editing in mammalian cells
Source: Nucleic Acids Res. 2022 Jun 10;50(11):6423–34. doi: 10.1093/nar/gkac506 (PMC9226529; doi:10.1093/nar/gkac506)
Supplement: gkac506_Supplemental_Files [file gkac506_supplemental_files.zip › Supplementary file 1.docx]

**Supplementary file 1**

**Contents**

Supplementary Figure 1. Targeted large fragment deletion by PE3 with a distal nick sgRNA.

Supplementary Figure 2. Representative Sanger sequencing results for Figure 1.

Supplementary Figure 3. Representative Sanger sequencing results of PE3 mediated large fragment deletions for Figure 1.

Supplementary Figure 4. Type II nicks improved PE3 mediated large fragment in K562 and HeLa cells.

Supplementary Figure 5. Deletion efficiencies determined by agarose gel analysis and capillary electrophoresis.

Supplementary Figure 6. Fragment deletions using Cas9 nickase (H840A), Bi-PE or Cas9 nuclease (WT-Cas9) in HEK293T cells.

Supplementary Figure 7. The effects of HA length on the efficiencies of large fragment deletions.

**
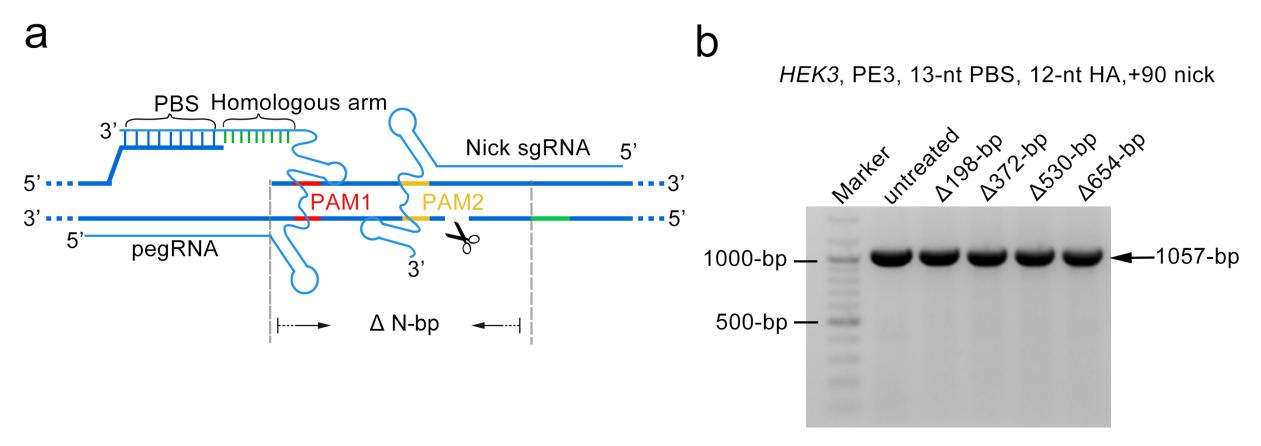
Supplementary Figure 1. Targeted large fragment deletion by PE3 with a distal nick sgRNA.**

**a.** Schematic diagram showing the design of PE3 strategy for targeted large fragment deletions with a nick sgRNA distal to the aimed deletion. **b.** Agarose gel analysis of the targeted deletions of indicated fragments.

**
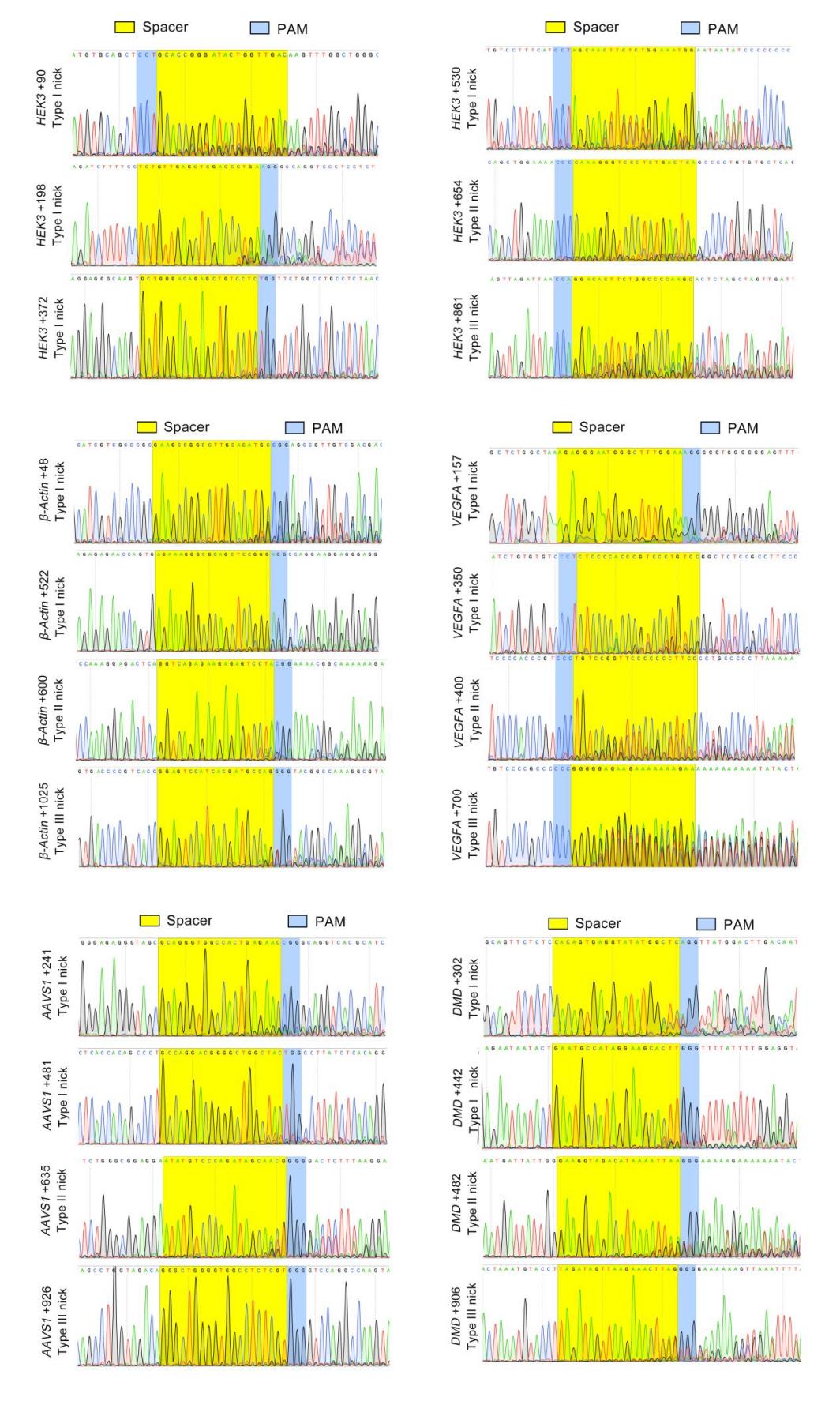
**

**Supplementary Figure 2. Representative Sanger sequencing results for Figure 1.**

The activity of indicated nick sgRNAs in wild-type Cas9 mediated indel formation on *HEK3*, *β-Actin*, *VEGFA, AAVS1* and *DMD* loci.


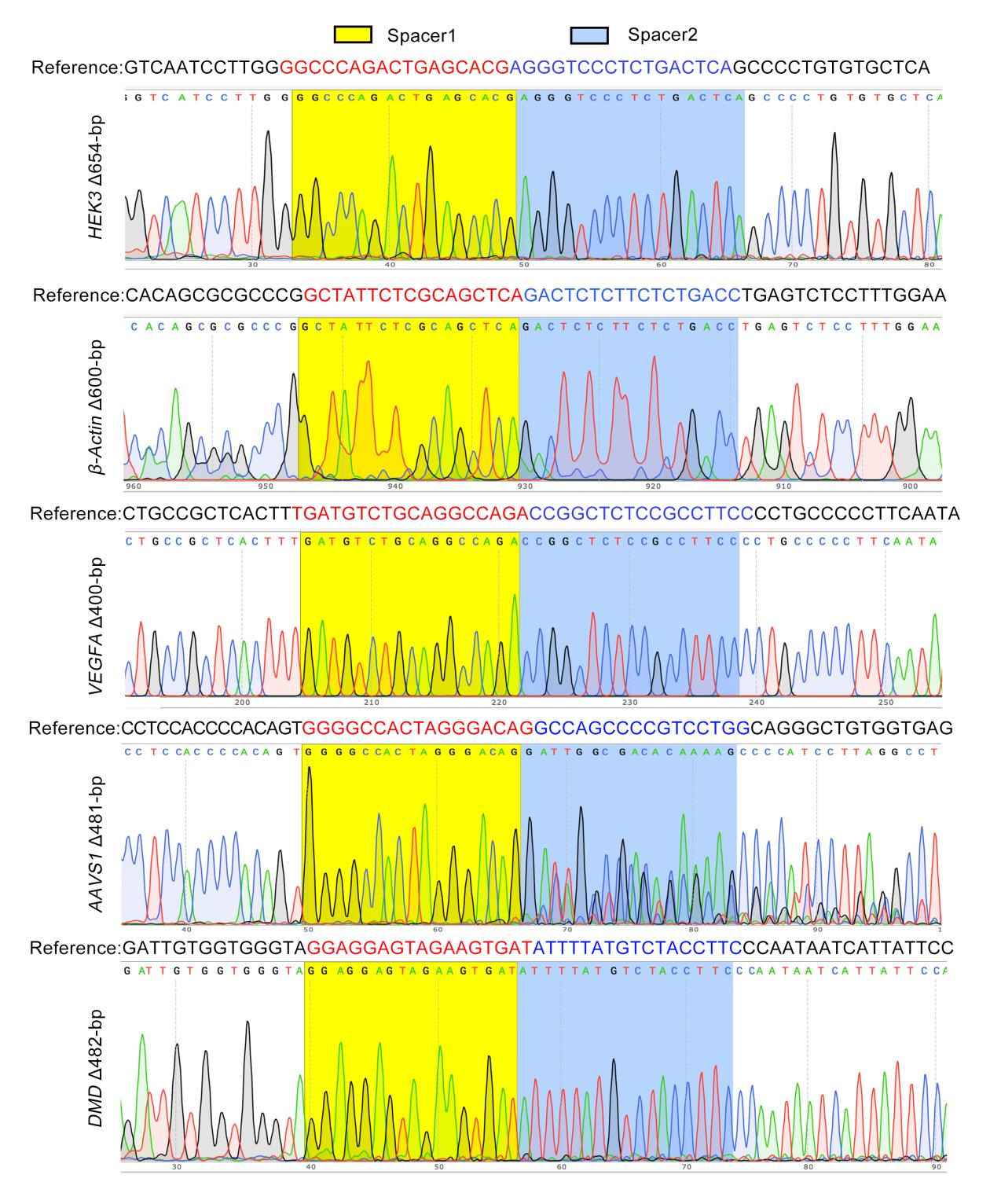


**Supplementary Figure 3. Representative Sanger sequencing results of PE3 mediated large fragment deletions for Figure 1.**

The PCR amplicons containing targeted deletions were gel-purified and subjected directly to Sanger sequencing.


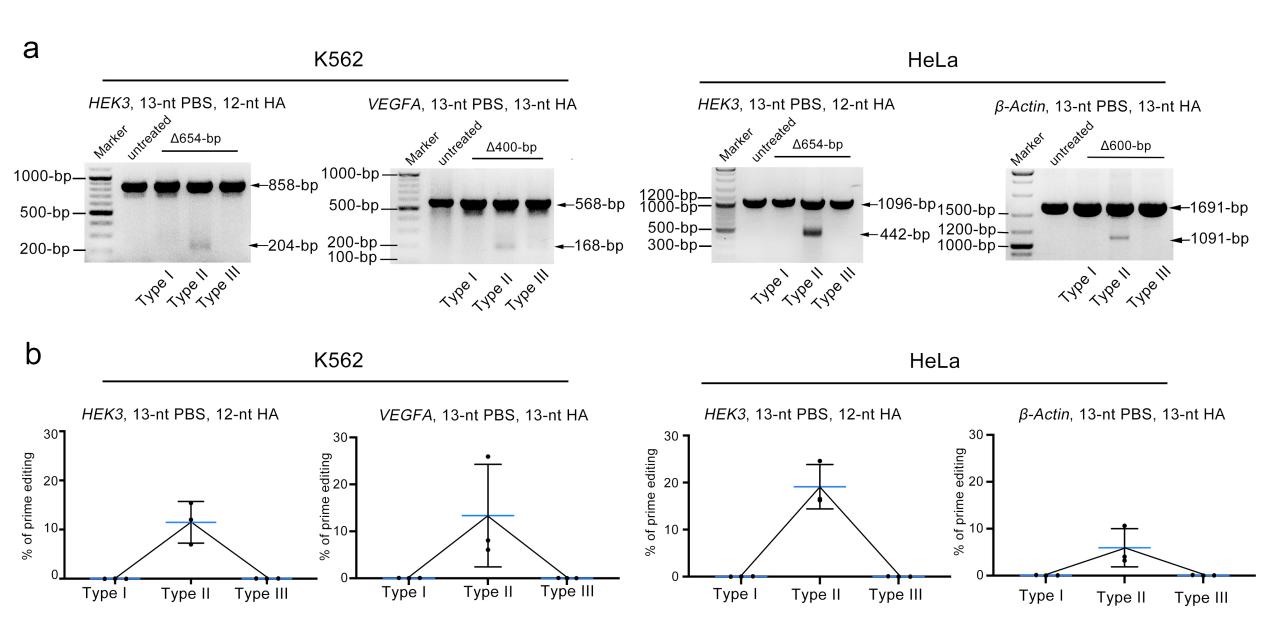


**Supplementary Figure 4. Type II nicks improved PE3 mediated large fragment in K562 and HeLa cells.**

**a.** Representative agarose gel electrophoresis detecting the presence of targeted deletions. Note that only type II nicks generated targeted deletions on *HEK3*, *VEGFA* and *β-Actin* loci in K562 and HeLa cells. **b.** Adobe Photoshop CC (2019) quantifying the efficiencies of the targeted deletions. Values and error bars reflect mean ± s.d. of n=3 independent biological replicates.

**
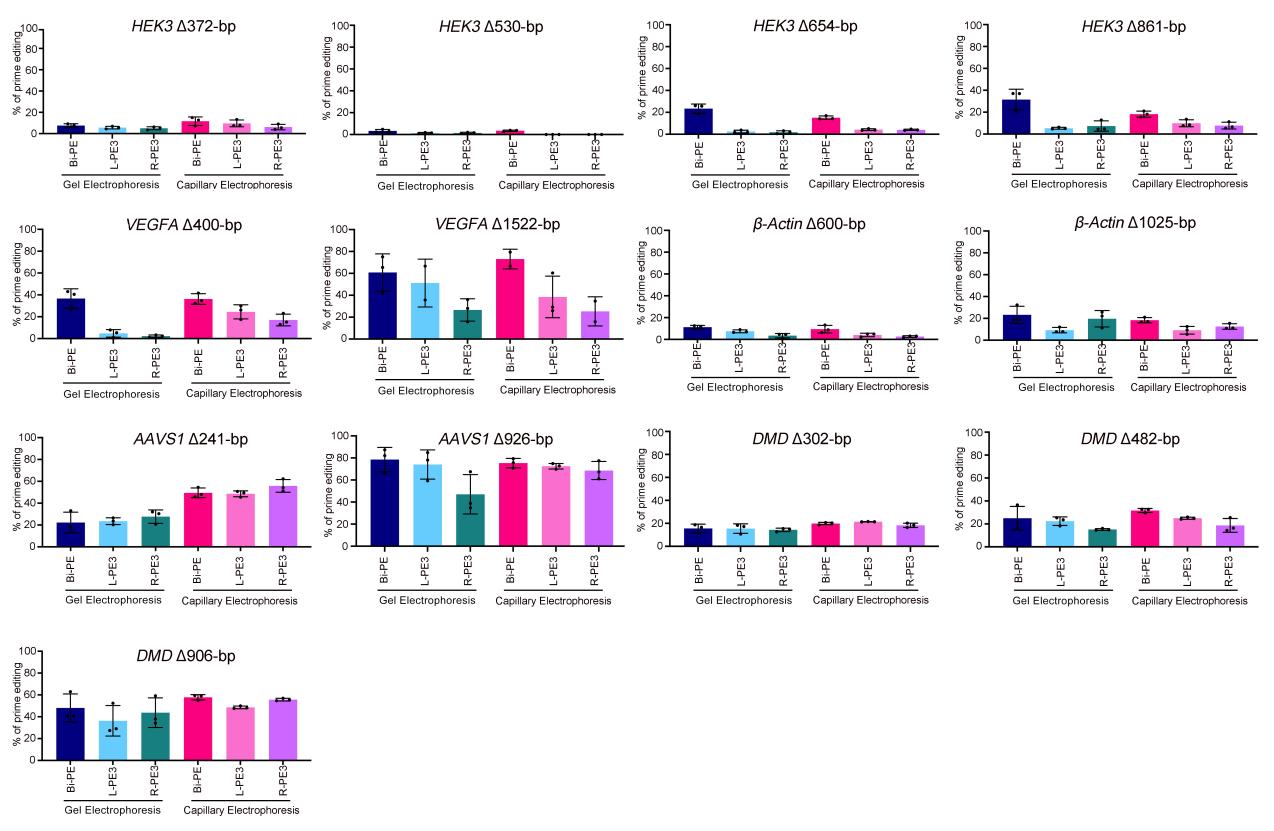
**

**Supplementary Figure 5. Deletion efficiencies determined by agarose gel analysis and capillary electrophoresis.**

The deletion efficiencies were quantified by either the gel electrophoresis or the capillary electrophoresis. Values and error bars reflect mean ± s.d. of n=3 independent biological replicates.

**
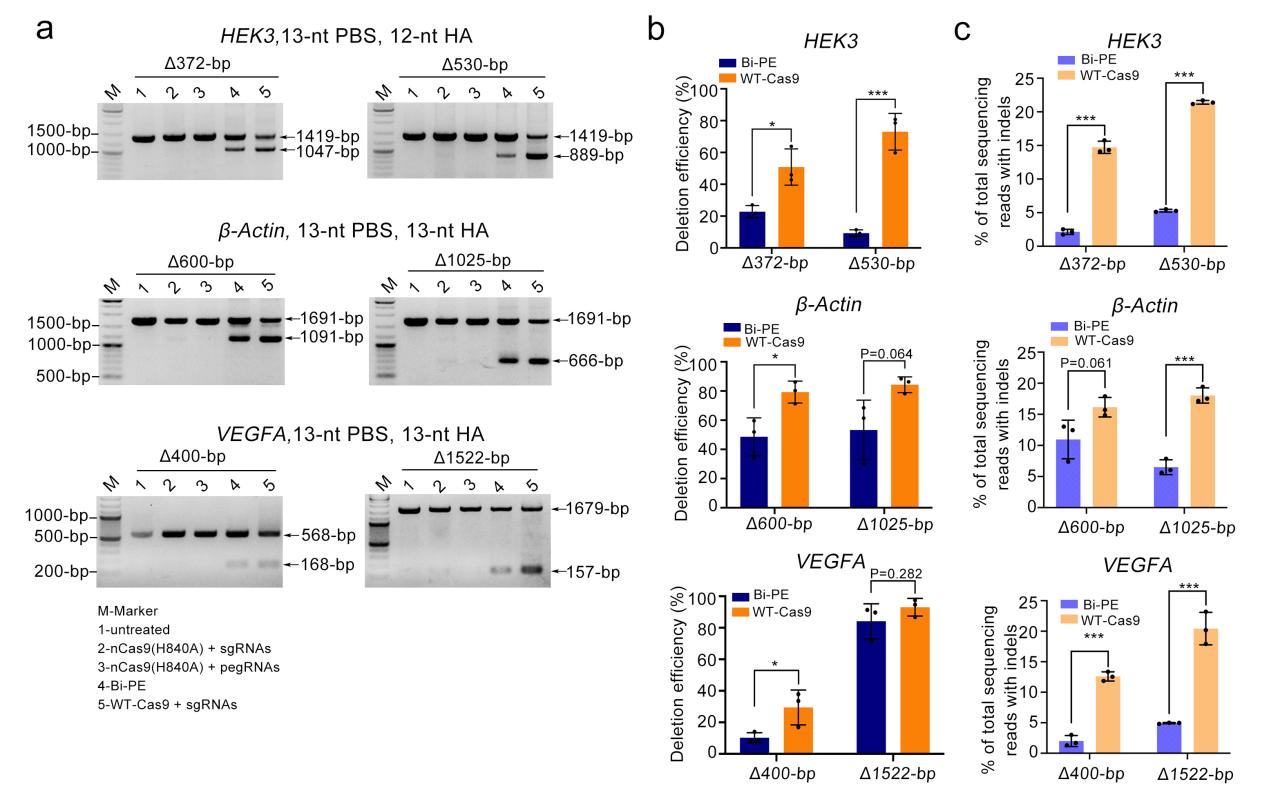
**

**Supplementary Figure 6. Fragment deletions using Cas9 nickase (H840A), Bi-PE or Cas9 nuclease (WT-Cas9) in HEK293T cells.**

**a.** Agarose gel analysis of the presence of targeted deletions on indicated loci. **b.** Quantification of the efficiencies of targeted deletions using Adobe Photoshop CC (2019). **c.** HTS analysis of the frequencies of undesired indels. The fragments containing deletions were gel purified and amplified by HTS primers. Then the products were subjected to HTS to analyze undesired indels. All alleles observed with frequency ≥0.01% are shown. Values and error bars reflect mean ± s.d. of n=3 independent biological replicates.


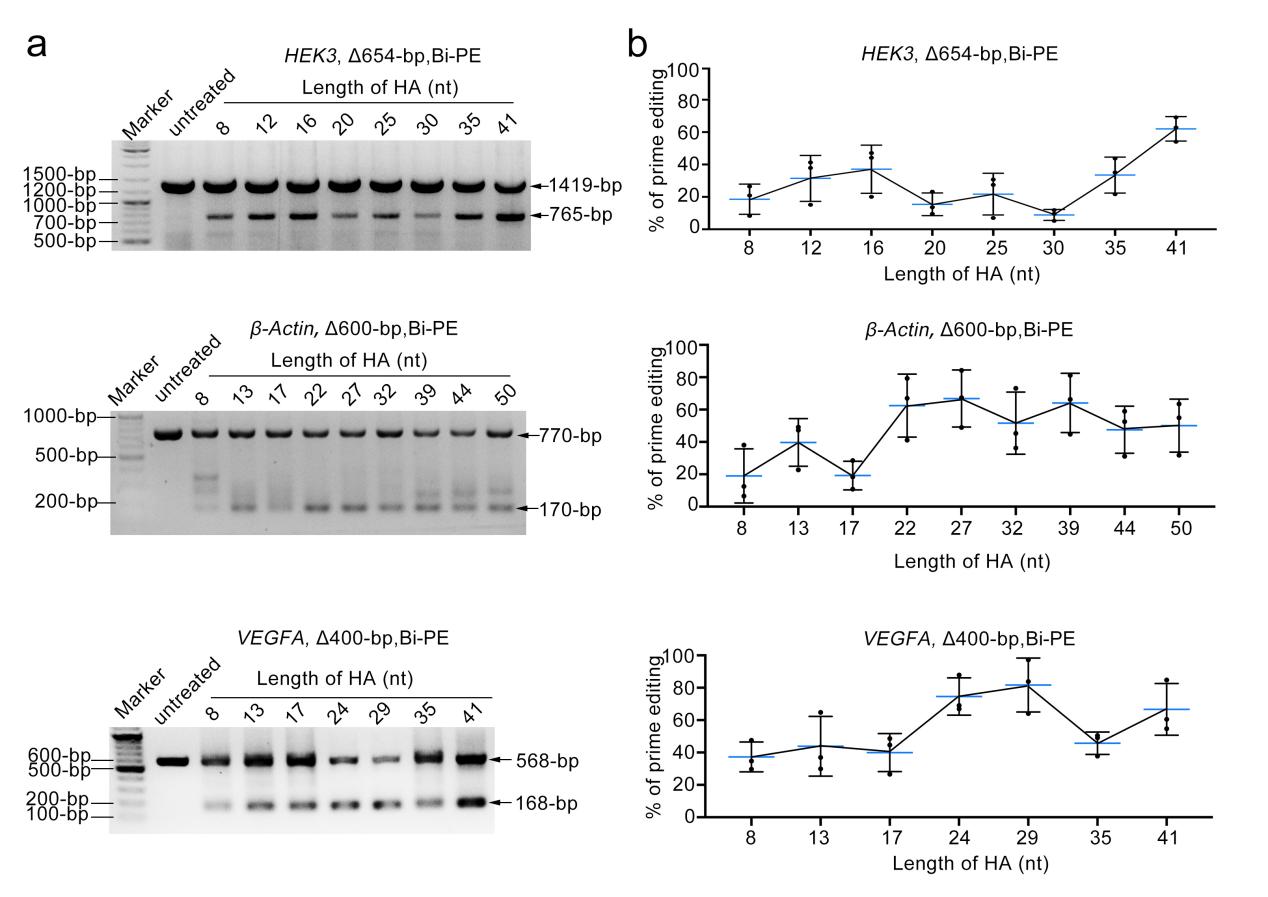


**Supplementary Figure 7. The effects of HA length on the efficiencies of large fragment deletions.**

**a.** Agarose gel analysis of the presence of targeted deletions on indicated loci. **b.** Quantification of the efficiency of targeted deletions using Adobe Photoshop CC (2019). Values and error bars reflect mean ± s.d. of n=3 independent biological replicates.
