# Supplementary material for: Bi-PE: bi-directional priming improves CRISPR/Cas9 prime editing in mammalian cells
Source: Nucleic Acids Res. 2022 Jun 10;50(11):6423–34. doi: 10.1093/nar/gkac506 (PMC9226529; doi:10.1093/nar/gkac506)
Supplement: gkac506_Supplemental_Files [file gkac506_supplemental_files.zip › Supplementary file 2.docx]

**Supplementary file 2**

**Contents**

Supplementary Figure 8. Bi-PE strategy increased the efficiency of fragment deletion in K562, HeLa and B16 cells.

Supplementary Figure 9. Sequence alignments showing the indels in PE3 and Bi-PE mediated 372-bp deletions of *HEK3* locus.

Supplementary Figure 10. Comparison of Bi-PE-3 and dual-sgRNA Cas9 nickase (H840A) in fragment replacement.

Supplementary Figure 11. The effect of HA length on replacement efficiency.

Supplementary Figure 12. The effect of replacement length on replacement efficiency.

Supplementary Figure 13. Sequence alignments showing the indels in fragment replacement of *HEK3* locus.

Supplementary Figure 14. Comparison of Bi-PE and PE3 strategies in single and double base conversions.


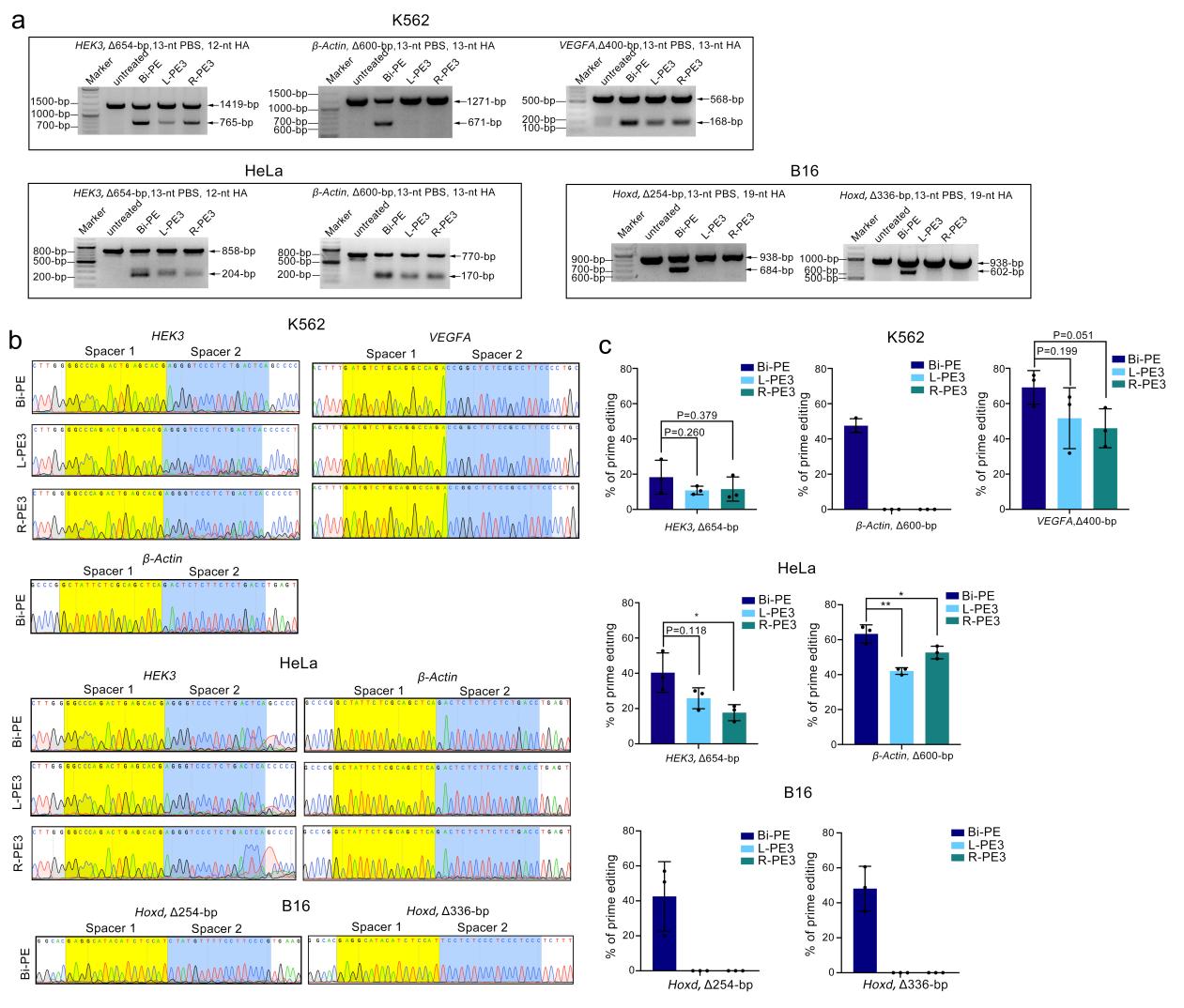


**Supplementary Figure 8. Bi-PE strategy increased the efficiency of fragment deletion in K562, HeLa and B16 cells.**

**a.** Representative agarose gel images showing the presence of targeted deletion in K562, HeLa and B16 cells. **b.** The amplicons containing targeted deletions were gel-purified and subjected directly for Sanger sequencing. Residue spacer sequences were marked with yellow or blue. **c.** Adobe Photoshop CC (2019) quantifying the efficiencies of the targeted deletions. Values and error bars reflect mean ± s.d. of n=3 independent biological replicates.

**
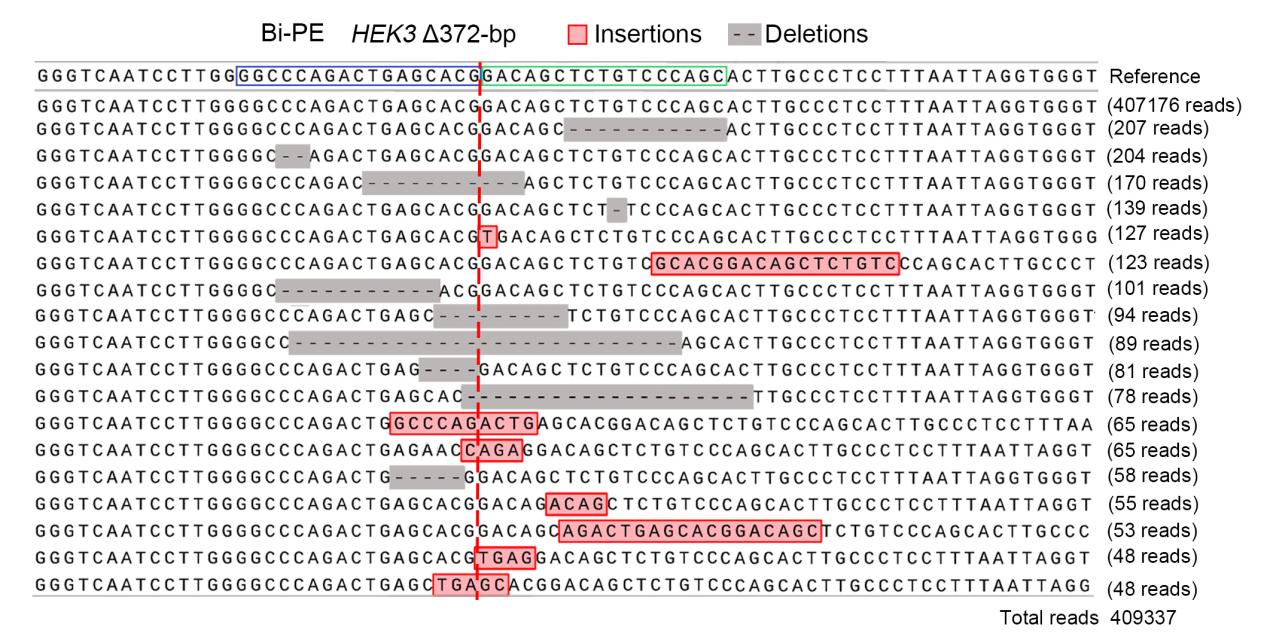
**

**
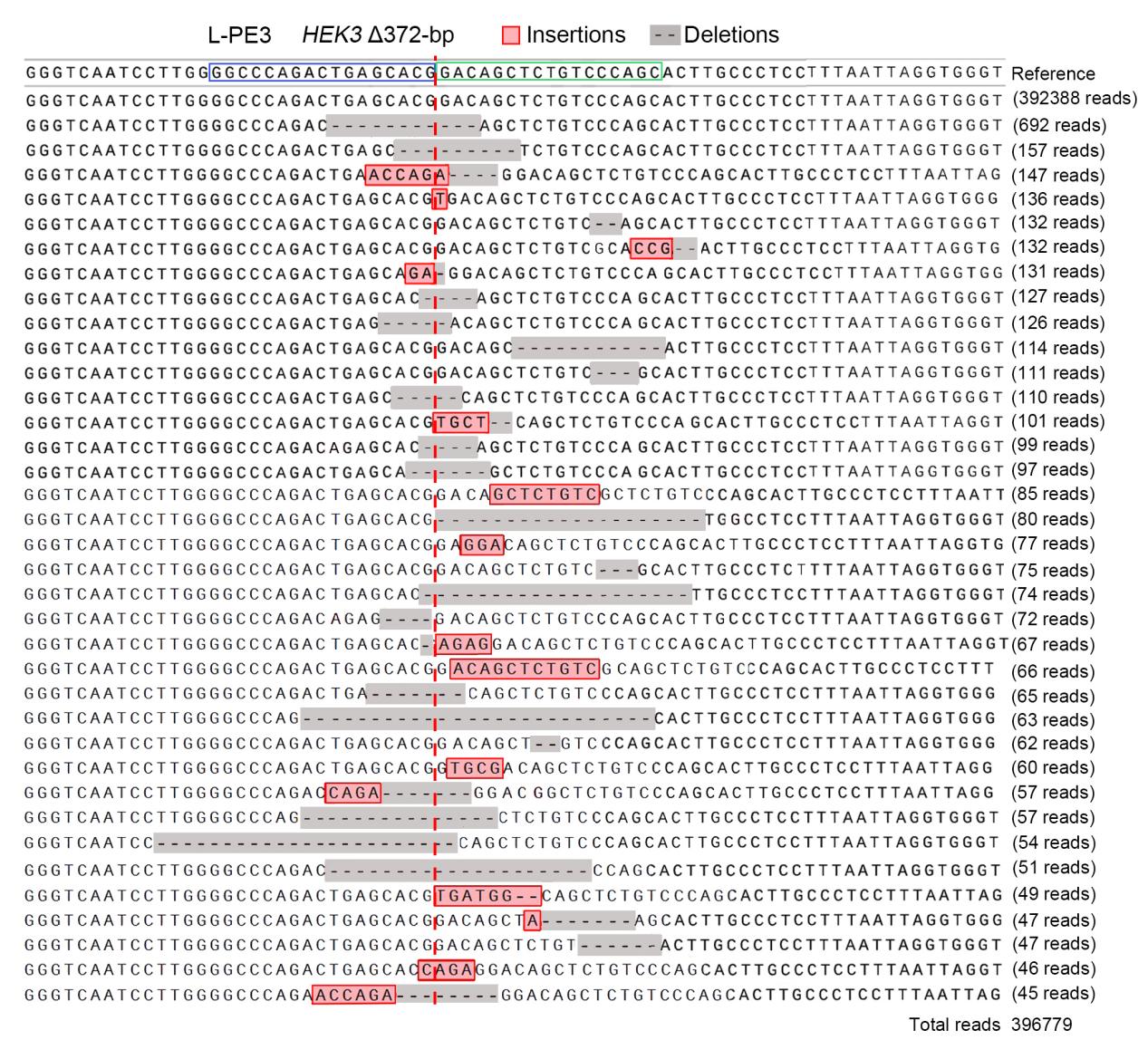
**

**
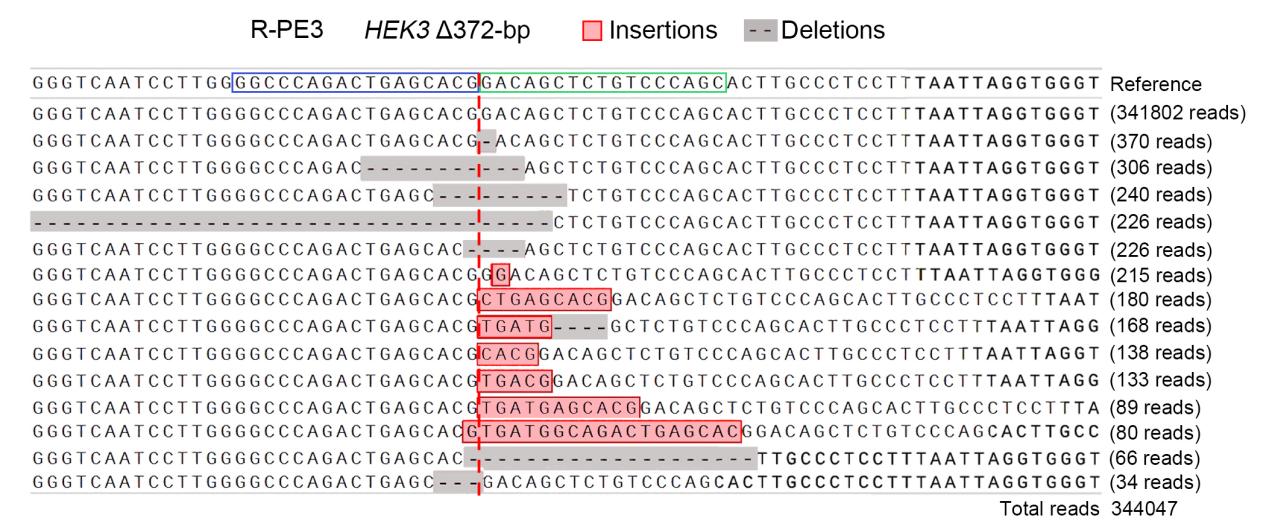
**

**Supplementary Figure 9. Sequence alignments showing the indels in PE3 and Bi-PE mediated 372-bp deletions of *HEK3* locus.**

The PCR amplicons containing each indicated targeted deletions were gel-purified, barcoded and then subjected for HTS. Reads with perfect designed deletions were recognized as accurate editing, and the ones with undesired indels were inaccurate. Sequences of perfect deletions were on top of each alignment, with upstream junctions marked with blue box and downstream junctions marked with green box.


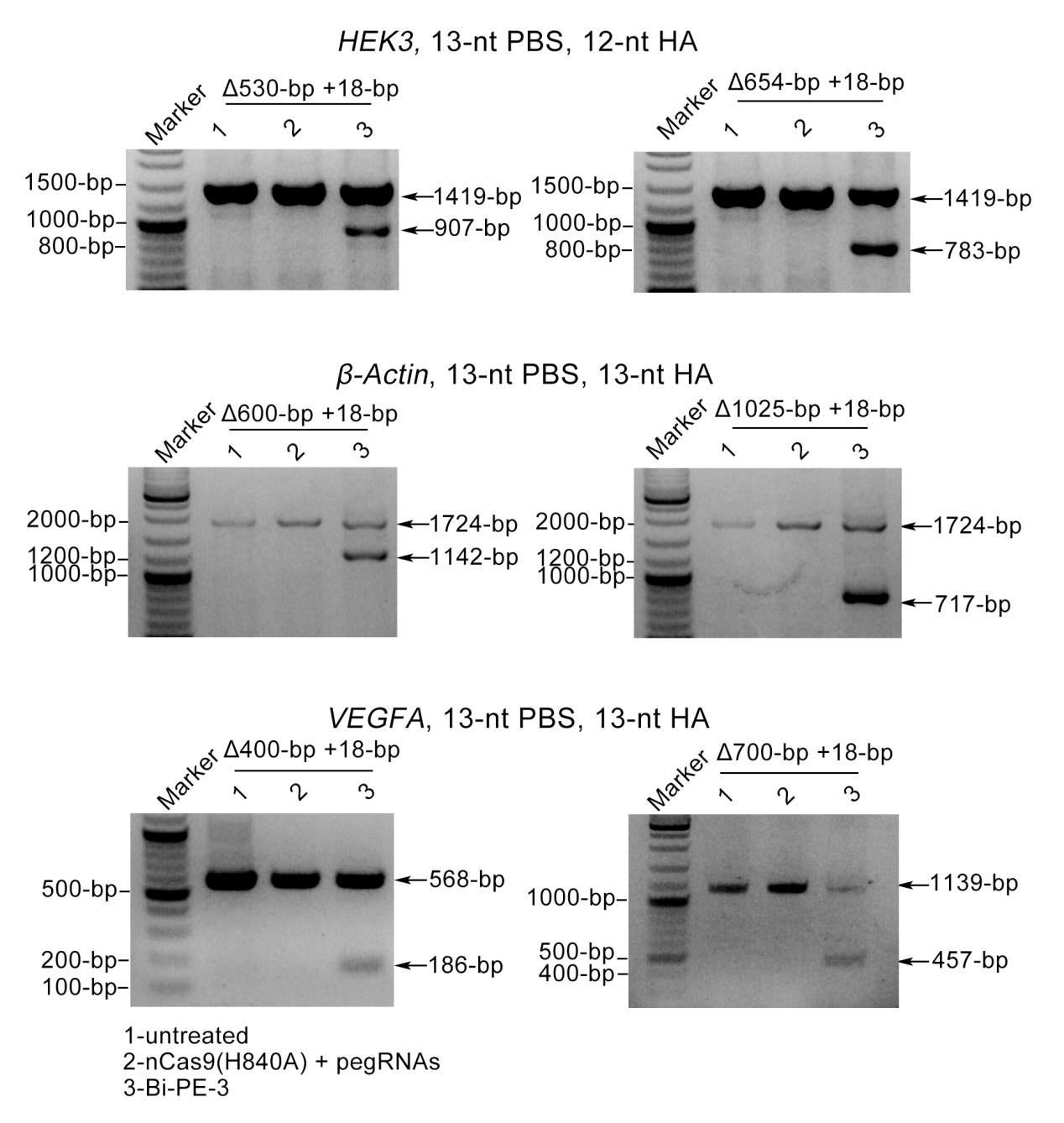


**Supplementary Figure 10. Comparison of Bi-PE-3 and dual-sgRNA Cas9 nickase (H840A) in fragment replacement.**

Agarose gel analysis of the presence of targeted replacement on indicated loci.


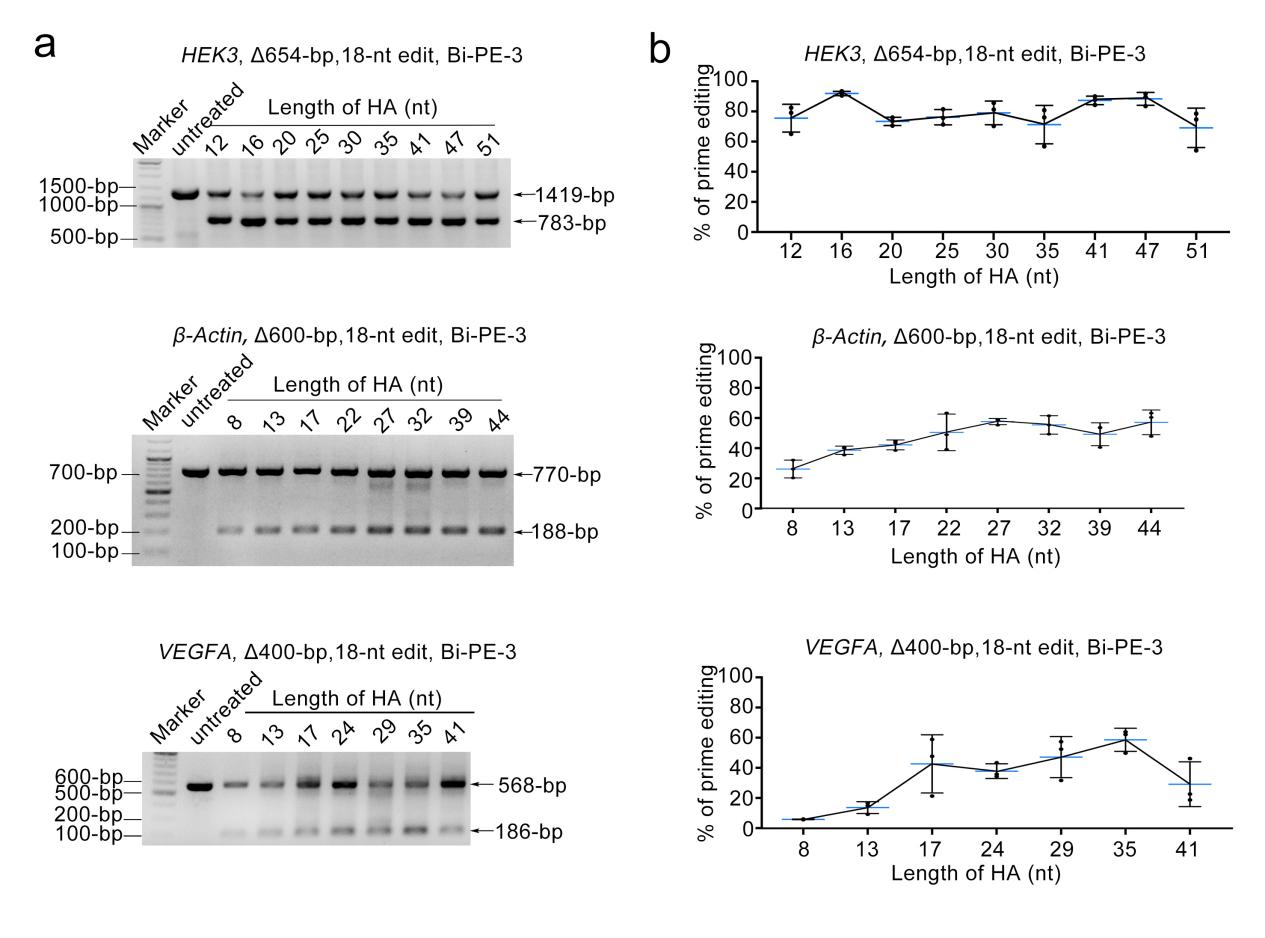


**Supplementary Figure 11. The effect of HA length on replacement efficiency.**

**a.** Agarose gel analysis of the presence of targeted replacement on indicated loci. **b.** Quantification of the efficiency of targeted replacement using Adobe Photoshop CC (2019). Values and error bars reflect mean ± s.d. of n=3 independent biological replicates.

**
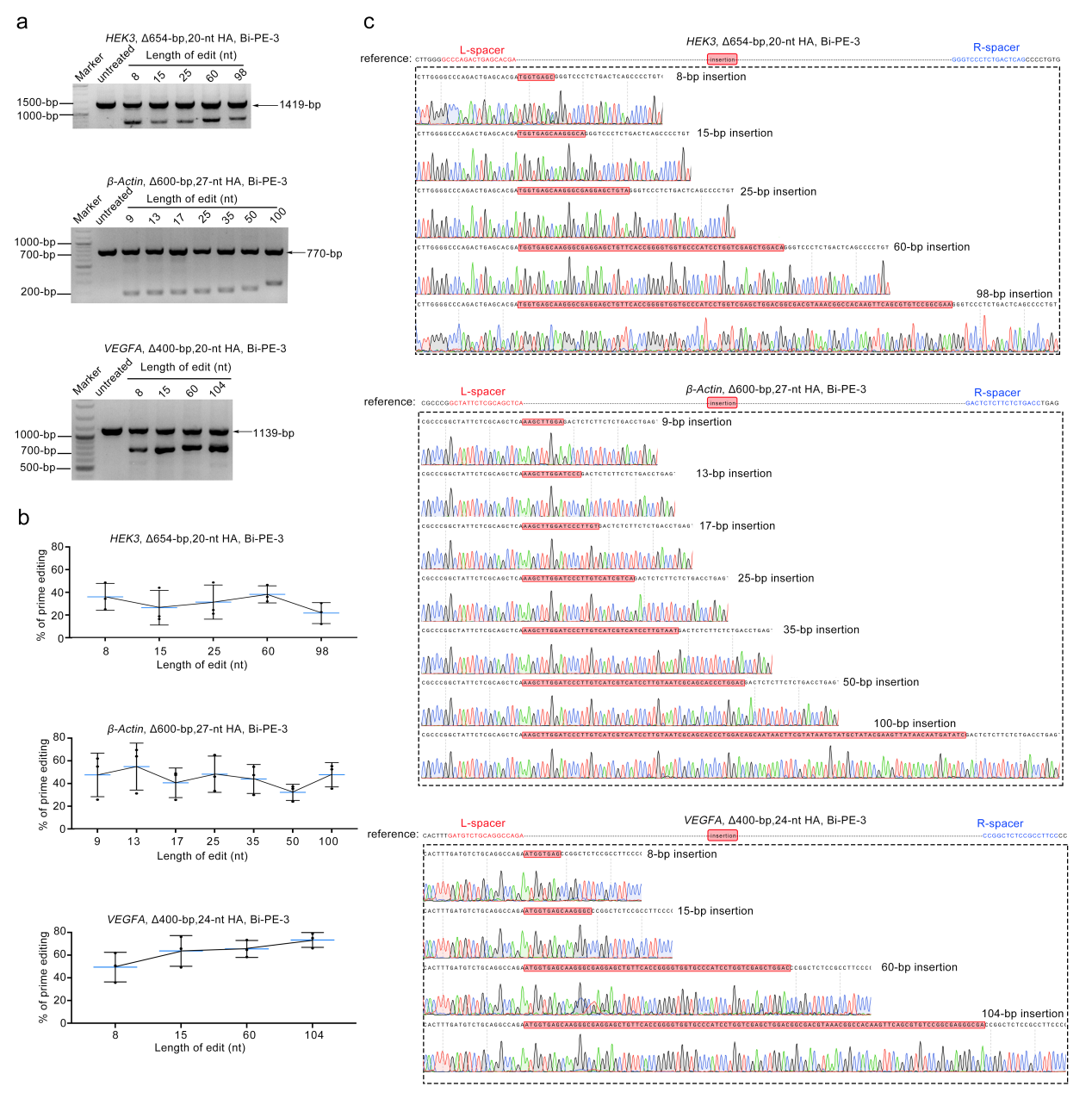
**

**Supplementary Figure 12. The effect of replacement length on replacement efficiency.**

**a.** Agarose gel analysis of the presence of targeted replacement on indicated loci. **b.** Quantification of the efficiency of targeted replacement using Adobe Photoshop CC (2019). Values and error bars reflect mean ± s.d. of n=3 independent biological replicates. **c.** The PCR amplicons containing targeted replacement were gel-purified and subjected directly to Sanger sequencing. Reference Sequences were shown on the top of each alignment, with spacers of L-pegRNA shown in red and spacers of R-pegRNA shown in blue. The replacement sequences were marked with red box.
