# Supplementary material for: Bi-PE: bi-directional priming improves CRISPR/Cas9 prime editing in mammalian cells
Source: Nucleic Acids Res. 2022 Jun 10;50(11):6423–34. doi: 10.1093/nar/gkac506 (PMC9226529; doi:10.1093/nar/gkac506)
Supplement: gkac506_Supplemental_Files [file gkac506_supplemental_files.zip › Supplementary file 3.docx]

**Supplementary file 3**

**Contents**

Supplementary Figure 13. Sequence alignments showing the indels in fragment replacement of *HEK3* locus.

Supplementary Figure 14. Comparison of Bi-PE and PE3 strategies in single and double base conversions.

Supplementary Figure 15. Heterogenicity of Bi-PE or PE3 mediated simultaneous conversion of multiple bases.

Supplementary Figure 16. Single clone analysis of Bi-PE mediated double-LoxP insertion in *HEK3* locus with a 90-bp flanking region.

Supplementary Figure 17. Single clone analysis of Bi-PE mediated double-LoxP insertion in *HEK3* locus with a 198-bp flanking region.

Supplementary Table 1. Sequences of pegRNAs used for in vitro experiments.

Supplementary Table 2. List of the targets tested in this study.

Supplementary Table 3. Summary of primers for amplification of each target sites.

Supplementary Table 4. HTS primers used for mammalian cell genomic DNA amplification.

Supplementary Table 5. Prime editing efficiency of each replicate in Figures 1-5.

Supplementary Note 1. Custom python script for HTS data analysis.

**
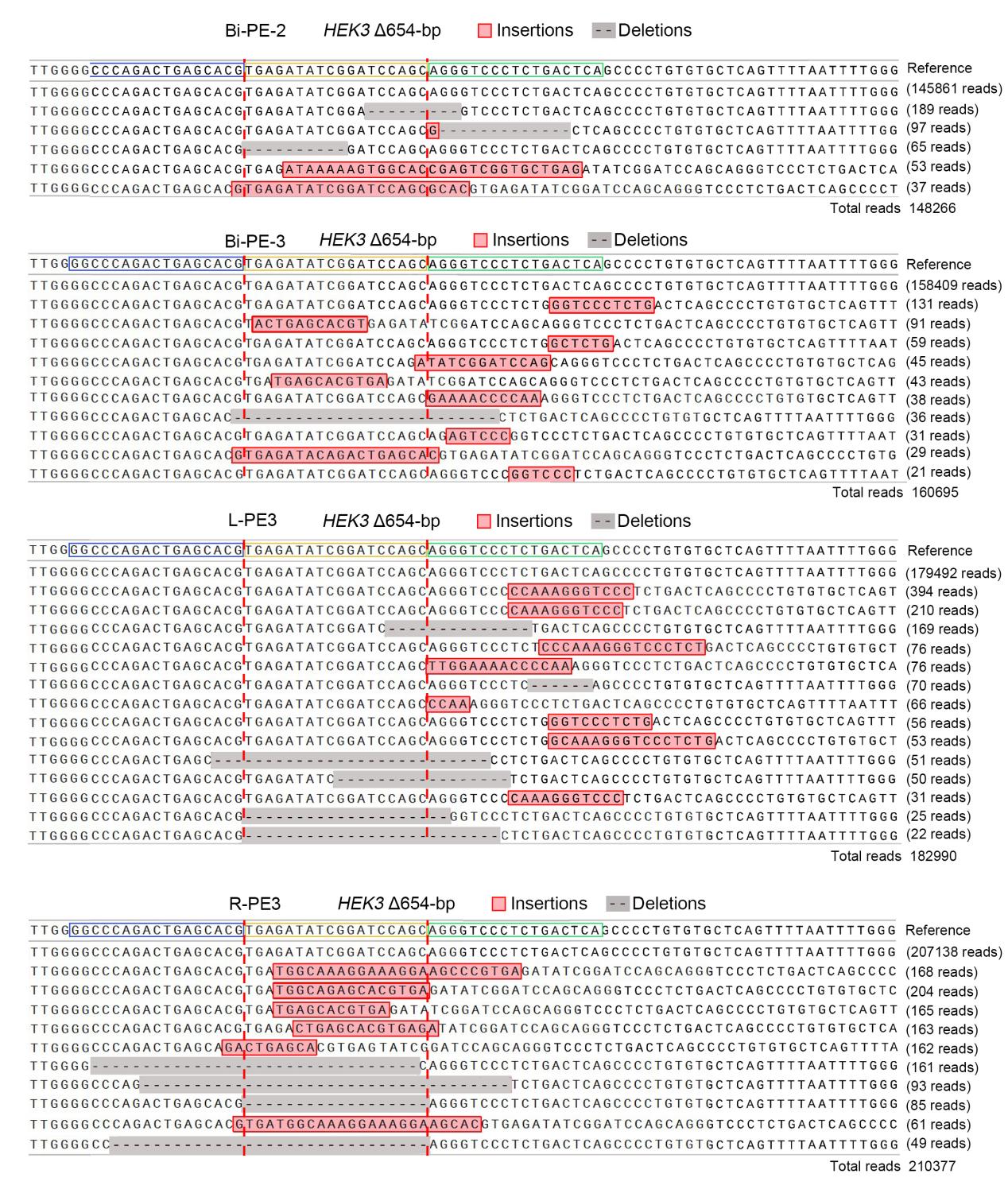
Supplementary Figure 13. Sequence alignments showing the indels in fragment replacement of *HEK3* locus.**

The PCR amplicons containing each indicated simultaneous deletion and insertion were gel-purified, barcoded and then subjected for HTS. Reads with perfect designed editing were recognized as accurate editing, and the ones with undesired indels were inaccurate. Sequences of perfect deletions were on top of each alignment, with upstream junctions marked with blue box, downstream junctions marked with green box and replacement sequences marked with yellow box.


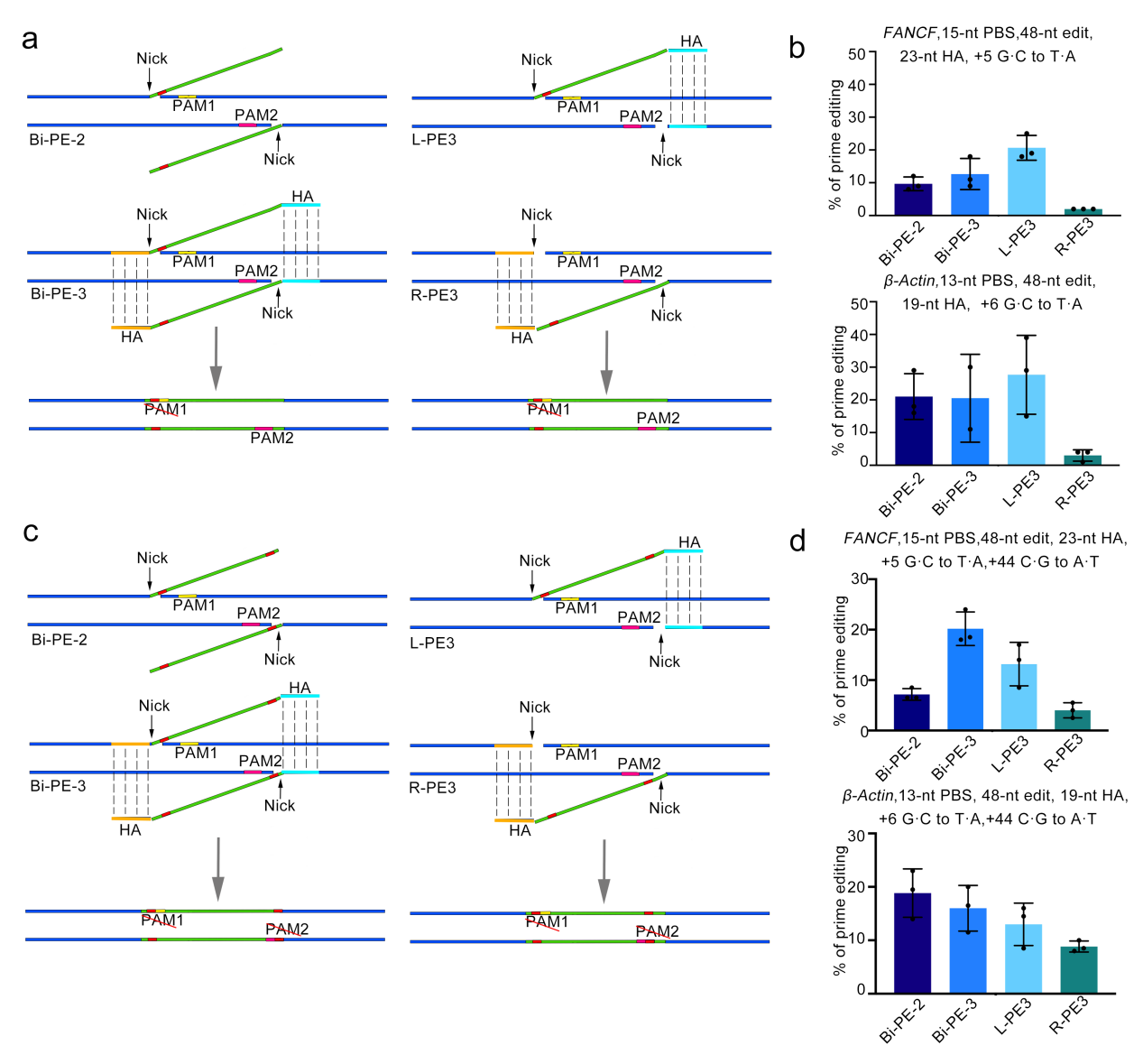


**Supplementary Figure 14. Comparison of Bi-PE and PE3 strategies in single and double base conversions.**

**a.** and **c.** Schematic diagram showing the design of PE3 and Bi-PE mediated single (**a**) and double (**c**) base conversions. **b.** and **d.** Quantification of the efficiency of targeted single base (**b**) and double base (**d**) conversions by Sanger sequencing. Values and error bars reflect mean±s.d. of n=3 independent biological replicates.


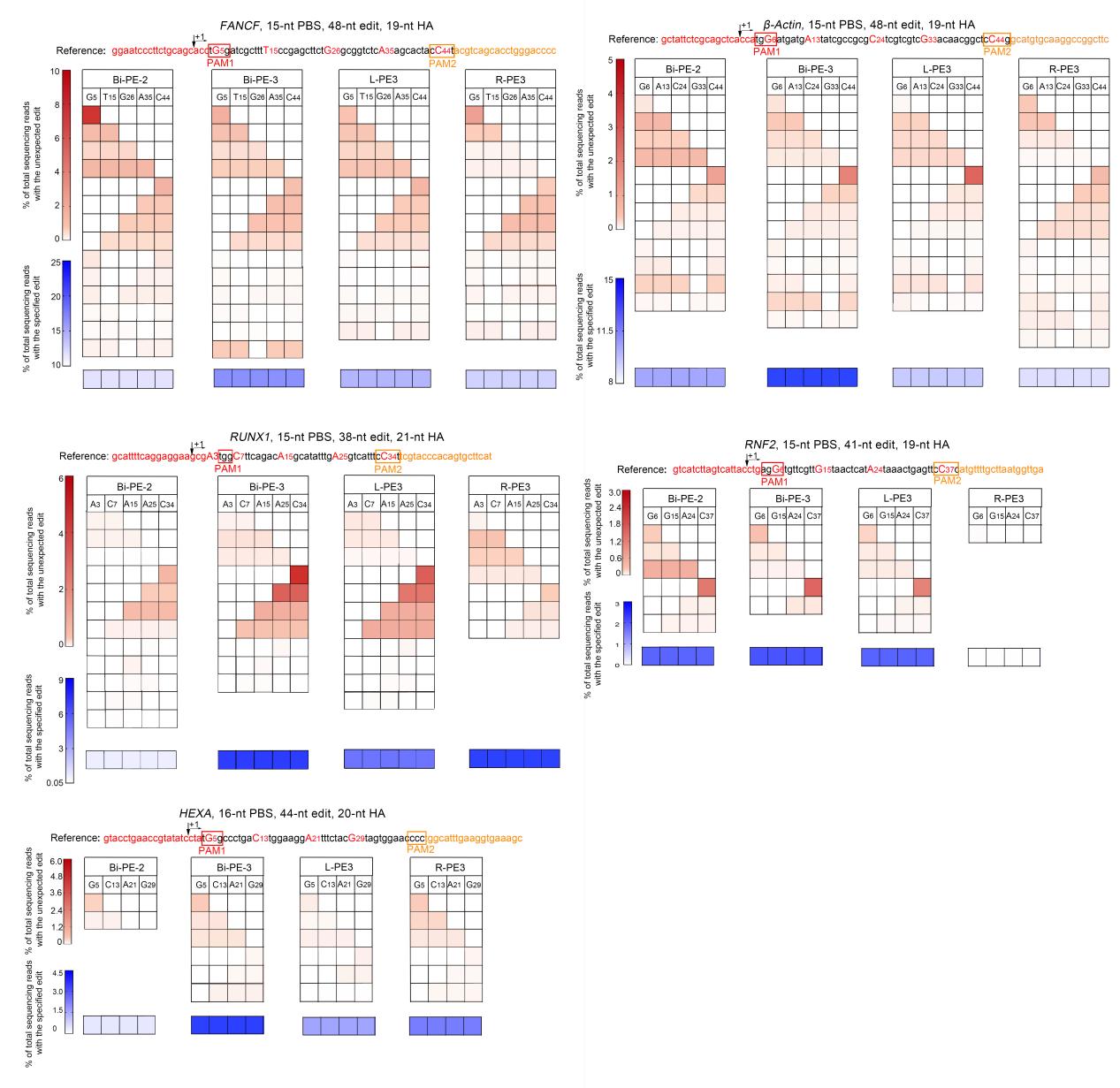


**Supplementary Figure 15. Heterogenicity of Bi-PE or PE3 mediated simultaneous conversion of multiple bases.**

Editing outcomes of multiple base conversion in *FANCF,* *β-Actin, RUNX1, RNF2 and HEXA* loci. Wild-type sequence was used as reference, with upstream PAMs boxed in red and downstream PAMs boxed in yellow. Bases to be converted was shown in red and numbered according to their relative positions to the upstream nick. Efficiencies of perfect editings (all targeted bases were converted) were valued by blue heat maps, and the ones of imperfect editings (at least one target base was not converted) were shown in brown heat maps. All alleles observed with frequency≥0.01% are shown.


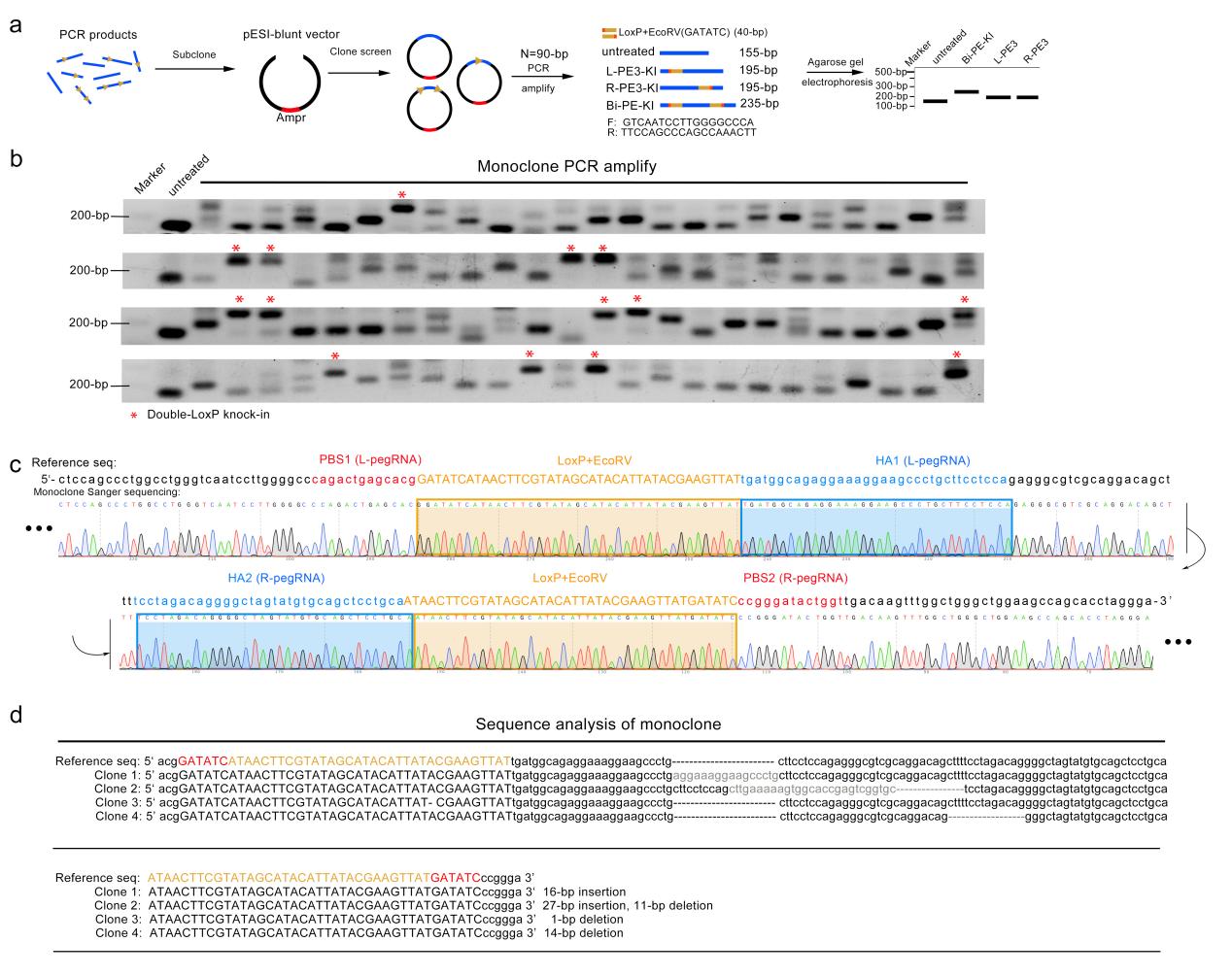


**Supplementary Figure 16. Single clone analysis of Bi-PE mediated double-LoxP insertion in *HEK3* locus with a 90-bp flanking region.**

**a.** Schematic diagram showing verification process of LoxP knock-in efficiency mediated by PE3 and Bi-PE. **b.** Gel electrophoresis diagram showing the LoxP knock-in on targeted loci by monoclonal PCR analysis. The size of the monoclonal PCR amplicons was as follows, negative: 155-bp, single-LoxP knock-in: 195-bp; double-LoxP knock-in: 235-bp. **c.** The double LoxP knock-in monoclone was subjected directly to Sanger sequencing. Reference Sequences were shown on top of the sequencing chromatogram, with PBS and HA of pegRNA were shown in red and blue respectively, HA shown in blue and genome sequences in black. The inserted sequences were marked with yellow box. **d.** Sequence alignments showing the indels in double-LoxP knock-in clones. Sequence of perfect knock-in was on top of alignment, with EcoRV shown in red and LoxP shown in yellow.


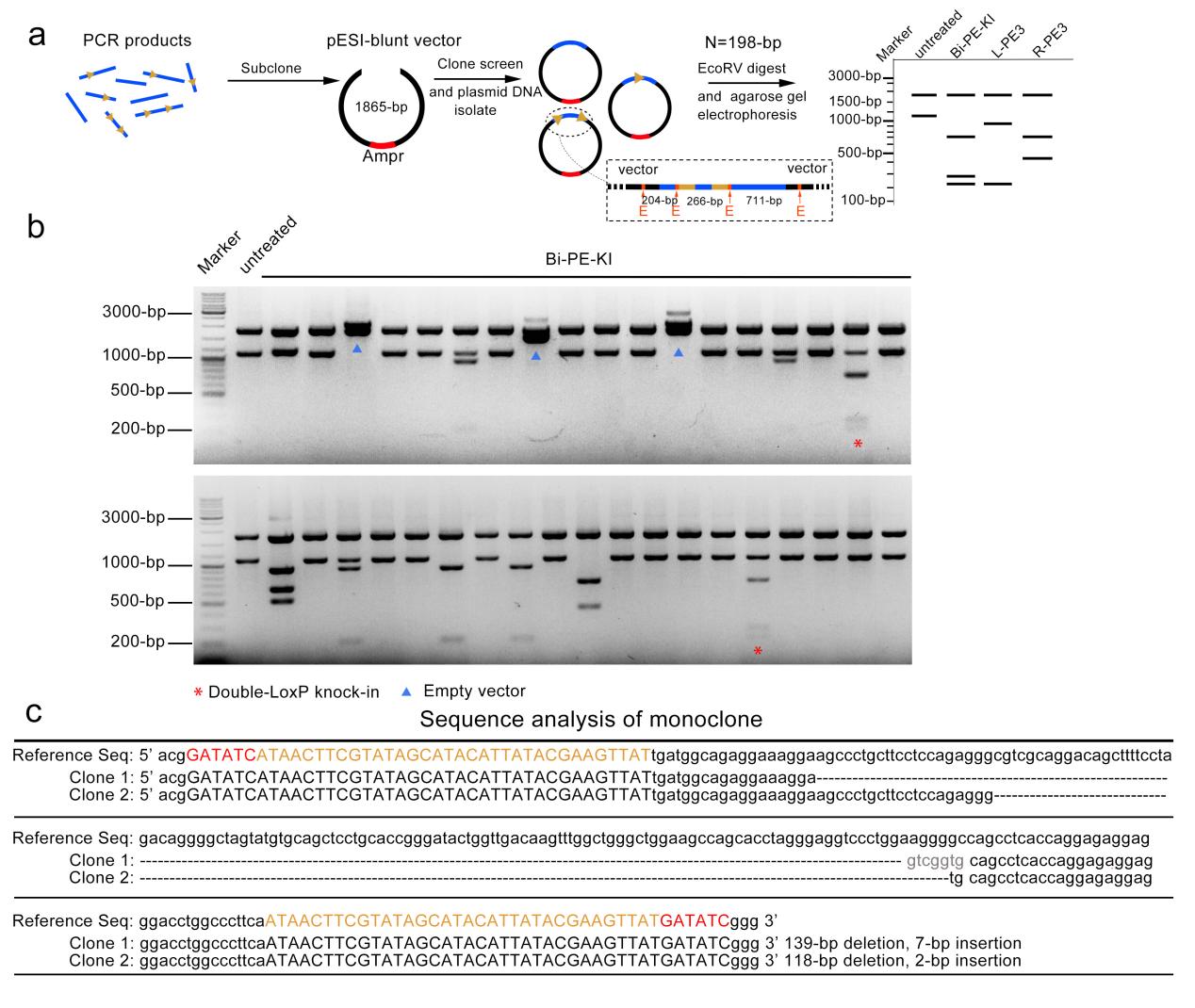


**Supplementary Figure 17. Single clone analysis of Bi-PE mediated double-LoxP insertion in *HEK3* locus with a 198-bp flanking region.**

**a.** Schematic diagram showing verification process of LoxP knock-in efficiency mediated by PE3 and Bi-PE. **b.** Gel electrophoresis diagram showing the LoxP knock-in on the targeted loci, which was analyzed by digesting the monoclonal plasmid with EcoRV. The size of the digestion products was as follows, negative (two bands): 1821-bp and 1105-bp; single-LoxP (three bands): 1821-bp, 715-bp, 430-bp or 1821-bp, 941-bp, 204-bp; double-LoxP (four bands): 1821-bp, 715-bp, 266-bp, 204-bp. **c.** Sequence alignments showing the indels in double-LoxP knock-in clones. Sequence of perfect knock-in was on top of alignment, with EcoRV shown in red and LoxP shown in yellow.

**Supplementary Table 1. Sequences of pegRNAs used for in vitro experiments.**

| pegRNA | Spacer | 3' extension (5' to 3') | PBS length  (nt) | HA  length (nt) | Edit  length (nt) |
| --- | --- | --- | --- | --- | --- |
| *HEK3*-Δ372 peg | GGCCCAGACTGAGCACGTGA | GACAGAGCTGTCCGTGCTCAGTCTG | 13 | 12 | 0 |
| *HEK3*-Δ530 peg | GGCCCAGACTGAGCACGTGA | AGACTGAGCACGAACTTCTCTGGAA | 13 | 12 | 0 |
| *HEK3*-Δ654 peg | GGCCCAGACTGAGCACGTGA | AGACTGAGCACGAGGGTCCCTCTGA | 13 | 12 | 0 |
| *HEK3*-Δ861 peg | GGCCCAGACTGAGCACGTGA | AGACTGAGCACGCACTTCTGGCCCC | 13 | 12 | 0 |
| *HEK3*+372 peg | GCTGGGACAGAGCTGTCCTC | AGACTGAGCACGGACAGCTCTGTCC | 13 | 12 | 0 |
| *HEK3*+530 peg | CCACTTCCAGAGAAGTTGCT | AGACTGAGCACGAACTTCTCTGGAA | 13 | 12 | 0 |
| *HEK3*+654 peg | TGAGTCAGAGGGACCCTTTG | AGACTGAGCACGAGGGTCCCTCTGA | 13 | 12 | 0 |
| *HEK3*+861 peg | GCTTGGGGCCAGAAGTGTCC | AGACTGAGCACGCACTTCTGGCCCC | 13 | 12 | 0 |
| Bi-PE-2-*HEK3* E18 peg | GGCCCAGACTGAGCACGTGA | GCTGGATCCGATATCTCACGTGCTCAGTCTG | 13 | 0 | 18 |
| Bi-PE-2-*HEK3* +530 peg | CCACTTCCAGAGAAGTTGCT | TGAGATATCGGATCCAGCAACTTCTCTGGAA | 13 | 0 | 18 |
| Bi-PE-2-*HEK3* +654 peg | TGAGTCAGAGGGACCCTTTG | TGAGATATCGGATCCAGCAGGGTCCCTCTGA | 13 | 0 | 18 |
| Bi-PE-2-*HEK3* +861 peg | GCTTGGGGCCAGAAGTGTCC | TGAGATATCGGATCCAGCCACTTCTGGCCCC | 13 | 0 | 18 |
| Bi-PE-3-*HEK3*-Δ530 peg | GGCCCAGACTGAGCACGTGA | TCCAGAGAAGTTGCTGGATCCGATATCTCACGTGCTCAGTCTG | 13 | 12 | 18 |
| Bi-PE-3-*HEK3*-Δ654 peg | GGCCCAGACTGAGCACGTGA | CAGAGGGACCCTGCTGGATCCGATATCTCACGTGCTCAGTCTG | 13 | 12 | 18 |
| Bi-PE-3-*HEK3*-Δ861 peg | GGCCCAGACTGAGCACGTGA | GGGCCAGAAGTGGCTGGATCCGATATCTCACGTGCTCAGTCTG | 13 | 12 | 18 |
| Bi-PE-3-*HEK3* +530 peg | CCACTTCCAGAGAAGTTGCT | AGACTGAGCACGTGAGATATCGGATCCAGCAACTTCTCTGGAA | 13 | 12 | 18 |
| Bi-PE-3-*HEK3* +654 peg | TGAGTCAGAGGGACCCTTTG | AGACTGAGCACGTGAGATATCGGATCCAGCAGGGTCCCTCTGA | 13 | 12 | 18 |
| Bi-PE-3-*HEK3* +861 peg | GCTTGGGGCCAGAAGTGTCC | AGACTGAGCACGTGAGATATCGGATCCAGCCACTTCTGGCCCC | 13 | 12 | 18 |
| *VEGFA*-Δ400 peg | GATGTCTGCAGGCCAGATGA | GCGGAGAGCCGGTCTGGCCTGCAGA | 13 | 12 | 0 |
| *VEGFA*-Δ700 peg | GATGTCTGCAGGCCAGATGA | GCCCCCGGCCCGCTCTGGCCTGCAGA | 13 | 13 | 0 |
| *VEGFA*-Δ1522 peg | GATGTCTGCAGGCCAGATGA | GAGCTAGCACTTCTCTGGCCTGCAGA | 13 | 13 | 0 |
| *VEGFA*+400 peg | GGAAGGCGGAGAGCCGGACA | TCTGCAGGCCAGACCGGCTCTCCGCC | 13 | 13 | 0 |
| *VEGFA*+700 peg | CCCCGCCCCCGGCCCGCCCC | TCTGCAGGCCAGAGCGGGCCGGGGGC | 13 | 13 | 0 |
| *VEGFA*+1522 peg | GCCCGAGCTAGCACTTCTCG | TCTGCAGGCCAGAGAAGTGCTAGCTCGGG | 16 | 13 | 0 |
| Bi-PE-2-*VEGFA* E18 peg | GATGTCTGCAGGCCAGATGA | GCTGGATCCGATATCTCATCTGGCCTGCAGA | 13 | 0 | 18 |
| Bi-PE-2-*VEGFA* +400 peg | GGAAGGCGGAGAGCCGGACA | TGAGATATCGGATCCAGCCCGGCTCTCCGCC | 13 | 0 | 18 |
| Bi-PE-2-*VEGFA* +700 peg | CCCCGCCCCCGGCCCGCCCC | TGAGATATCGGATCCAGCGCGGGCCGGGGGC | 13 | 0 | 18 |
| Bi-PE-2-*VEGFA* +1522 peg | GCCCGAGCTAGCACTTCTCG | TGAGATATCGGATCCAGCGAAGTGCTAGCTCGGG | 16 | 0 | 18 |
| Bi-PE-3-*VEGFA*-Δ400 peg | GATGTCTGCAGGCCAGATGA | GGCGGAGAGCCGGGCTGGATCCGATATCTCATCTGGCCTGCAGA | 13 | 13 | 18 |
| Bi-PE-3-*VEGFA*-Δ700 peg | GATGTCTGCAGGCCAGATGA | GCCCCCGGCCCGCGCTGGATCCGATATCTCATCTGGCCTGCAGA | 13 | 13 | 18 |
| Bi-PE-3-*VEGFA*-Δ1522 peg | GATGTCTGCAGGCCAGATGA | GAGCTAGCACTTCGCTGGATCCGATATCTCATCTGGCCTGCAGA | 13 | 13 | 18 |
| Bi-PE-3-*VEGFA* +400 peg | GGAAGGCGGAGAGCCGGACA | TCTGCAGGCCAGATGAGATATCGGATCCAGCCCGGCTCTCCGCC | 13 | 13 | 18 |
| Bi-PE-3-*VEGFA* +700 peg | CCCCGCCCCCGGCCCGCCCC | TCTGCAGGCCAGATGAGATATCGGATCCAGCGCGGGCCGGGGGC | 13 | 13 | 18 |
| Bi-PE-3-*VEGFA* +1522 peg | GCCCGAGCTAGCACTTCTCG | TCTGCAGGCCAGATGAGATATCGGATCCAGCGAAGTGCTAGCTCGGG | 16 | 13 | 18 |
| *β-Actin*-Δ315 peg | GCTATTCTCGCAGCTCACCA | GATGCCTCTCTTGTGAGCTGCGAGAA | 13 | 13 | 0 |
| *β-Actin*-Δ600 peg | GCTATTCTCGCAGCTCACCA | AGAGAAGAGAGTCTGAGCTGCGAGAA | 13 | 13 | 0 |
| *β-Actin*-Δ1025 peg | GCTATTCTCGCAGCTCACCA | TCCATCACGATGCTGAGCTGCGAGAA | 13 | 13 | 0 |
| *β-Actin*+315 peg | TGAGGATGCCTCTCTTGCTC | TCTCGCAGCTCACAAGAGAGGCATCCT | 15 | 12 | 0 |
| *β-Actin*+600 peg | GGTCAGAGAAGAGAGTCCTA | TCTCGCAGCTCAGACTCTCTTCTCTGA | 15 | 12 | 0 |
| *β-Actin*+1025 peg | GGAGTCCATCACGATGCCAG | TCTCGCAGCTCAGCATCGTGATGGA | 13 | 12 | 0 |
| Bi-PE-2-*β-Actin* E18 peg | GCTATTCTCGCAGCTCACCA | GCTGGATCCGATATCTCATGAGCTGCGAGAA | 13 | 0 | 18 |
| Bi-PE-2-*β-Actin* +315 peg | TGAGGATGCCTCTCTTGCTC | GCTGGATCCGATATCTCACAAGAGAGGCATCCT | 15 | 0 | 18 |
| Bi-PE-2-*β-Actin* +600 peg | GGTCAGAGAAGAGAGTCCTA | GCTGGATCCGATATCTCAGACTCTCTTCTCTGA | 15 | 0 | 18 |
| Bi-PE-2-*β-Actin* +1025 peg | GGAGTCCATCACGATGCCAG | GCTGGATCCGATATCTCAGCATCGTGATGGA | 13 | 0 | 18 |
| Bi-PE-3-*β-Actin*-Δ315 peg | GCTATTCTCGCAGCTCACCA | GATGCCTCTCTTGTGAGATATCGGATCCAGCTGAGCTGCGAGAA | 13 | 13 | 18 |
| Bi-PE-3-*β-Actin*-Δ600 peg | GCTATTCTCGCAGCTCACCA | AGAGAAGAGAGTCTGAGATATCGGATCCAGCTGAGCTGCGAGAA | 13 | 13 | 18 |
| Bi-PE-3-*β-Actin*-Δ1025 peg | GCTATTCTCGCAGCTCACCA | TCCATCACGATGCTGAGATATCGGATCCAGCTGAGCTGCGAGAA | 13 | 13 | 18 |
| Bi-PE-3-*β-Actin* +315 peg | TGAGGATGCCTCTCTTGCTC | TCTCGCAGCTCAGCTGGATCCGATATCTCACAAGAGAGGCATCCT | 15 | 12 | 18 |
| Bi-PE-3-*β-Actin* +600 peg | GGTCAGAGAAGAGAGTCCTA | TCTCGCAGCTCAGCTGGATCCGATATCTCAGACTCTCTTCTCTGA | 15 | 12 | 18 |
| Bi-PE-3-*β-Actin* +1025 peg | GGAGTCCATCACGATGCCAG | TCTCGCAGCTCAGCTGGATCCGATATCTCAGCATCGTGATGGA | 13 | 12 | 18 |
| Bi-PE-2-S-*FANCF* | GGAATCCCTTCTGCAGCACC | CGTAGGTAGTGCTTGAGACCGCCAGAAGCTCGGAAAAGCGATCAAGGTGCTGCAGAAGGGATT | 15 | 0 | 48 |
| Bi-PE-2-S-*FANCF*+48 | GGGGTCCCAGGTGCTGACGT | ACCTTGATCGCTTTTCCGAGCTTCTGGCGGTCTCAAGCACTACCTACGTCAGCACCTGGGA | 13 | 0 | 48 |
| Bi-PE-3-S-*FANCF* | GGAATCCCTTCTGCAGCACC | GGTGGCGGGGTCCCAGGTGCTGACGTAGGTAGTGCTTGAGACCGCCAGAAGCTCGGAAAAGCGATCAAGGTGCTGCAGAAGGGATT | 15 | 23 | 48 |
| Bi-PE-3-S-*FANCF*+48 | GGGGTCCCAGGTGCTGACGT | CCTCATGGAATCCCTTCTGCAGCACCTTGATCGCTTTTCCGAGCTTCTGGCGGTCTCAAGCACTACCTACGTCAGCACCTGGGA | 13 | 23 | 48 |
| Bi-PE-2-D-*FANCF* | GGAATCCCTTCTGCAGCACC | CGTATGTAGTGCTTGAGACCGCCAGAAGCTCGGAAAAGCGATCAAGGTGCTGCAGAAGGGATT | 15 | 0 | 48 |
| Bi-PE-2-D-*FANCF*+48 | GGGGTCCCAGGTGCTGACGT | ACCTTGATCGCTTTTCCGAGCTTCTGGCGGTCTCAAGCACTACATACGTCAGCACCTGGGA | 13 | 0 | 48 |
| Bi-PE-3-D-*FANCF* | GGAATCCCTTCTGCAGCACC | GGTGGCGGGGTCCCAGGTGCTGACGTATGTAGTGCTTGAGACCGCCAGAAGCTCGGAAAAGCGATCAAGGTGCTGCAGAAGGGATT | 15 | 23 | 48 |
| Bi-PE-3-D-*FANCF*+48 | GGGGTCCCAGGTGCTGACGT | CCTCATGGAATCCCTTCTGCAGCACCTTGATCGCTTTTCCGAGCTTCTGGCGGTCTCAAGCACTACATACGTCAGCACCTGGGA | 13 | 23 | 48 |
| Bi-PE-2-M-*FANCF* | GGAATCCCTTCTGCAGCACC | CGTATGTAGTGCTAGAGACCGCTAGAAGCTCGGCAAAGCGATCAAGGTGCTGCAGAAGGGATT | 15 | 0 | 48 |
| Bi-PE-2-M-*FANCF*+48 | GGGGTCCCAGGTGCTGACGT | ACCTTGATCGCTTTGCCGAGCTTCTAGCGGTCTCTAGCACTACATACGTCAGCACCTGGGA | 13 | 0 | 48 |
| Bi-PE-3-M-*FANCF* | GGAATCCCTTCTGCAGCACC | GGTGGCGGGGTCCCAGGTGCTGACGTATGTAGTGCTAGAGACCGCTAGAAGCTCGGCAAAGCGATCAAGGTGCTGCAGAAGGGATT | 15 | 23 | 48 |
| Bi-PE-3-M-*FANCF*+48 | GGGGTCCCAGGTGCTGACGT | CCTCATGGAATCCCTTCTGCAGCACCTTGATCGCTTTGCCGAGCTTCTAGCGGTCTCTAGCACTACATACGTCAGCACCTGGGA | 13 | 23 | 48 |
| Bi-PE-2-S-*β-Actin* | GCTATTCTCGCAGCTCACCA | TGCCGGAGCCGTTGTCGACGACGAGCGCGGCGATATCATCATACATGGTGAGCTGCGAGAA | 13 | 0 | 48 |
| Bi-PE-2-S-*β-Actin*+48 | GAAGCCGGCCTTGCACATGC | CCATGTATGATGATATCGCCGCGCTCGTCGTCGACAACGGCTCCGGCATGTGCAAGGCCGG | 13 | 0 | 48 |
| Bi-PE-3-S-*β-Actin* | GCTATTCTCGCAGCTCACCA | GCGAAGCCGGCCTTGCACATGCCGGAGCCGTTGTCGACGACGAGCGCGGCGATATCATCATACATGGTGAGCTGCGAGAA | 13 | 19 | 48 |
| Bi-PE-3-S-*β-Actin*+48 | GAAGCCGGCCTTGCACATGC | CGGCTATTCTCGCAGCTCACCATGTATGATGATATCGCCGCGCTCGTCGTCGACAACGGCTCCGGCATGTGCAAGGCCGG | 13 | 19 | 48 |
| Bi-PE-2-D-*β-Actin* | GCTATTCTCGCAGCTCACCA | TGCCTGAGCCGTTGTCGACGACGAGCGCGGCGATATCATCATACATGGTGAGCTGCGAGAA | 13 | 0 | 48 |
| Bi-PE-2-D-*β-Actin*+48 | GAAGCCGGCCTTGCACATGC | CCATGTATGATGATATCGCCGCGCTCGTCGTCGACAACGGCTCAGGCATGTGCAAGGCCGG | 13 | 0 | 48 |
| Bi-PE-3-D-*β-Actin* | GCTATTCTCGCAGCTCACCA | GCGAAGCCGGCCTTGCACATGCCTGAGCCGTTGTCGACGACGAGCGCGGCGATATCATCATACATGGTGAGCTGCGAGAA | 13 | 19 | 48 |
| Bi-PE-3-D-*β-Actin*+48 | GAAGCCGGCCTTGCACATGC | CGGCTATTCTCGCAGCTCACCATGTATGATGATATCGCCGCGCTCGTCGTCGACAACGGCTCAGGCATGTGCAAGGCCGG | 13 | 19 | 48 |
| Bi-PE-2-M-*β-Actin* | GCTATTCTCGCAGCTCACCA | TGCCTGAGCCGTTGTAGACGACGATCGCGGCGATAGCATCATACATGGTGAGCTGCGAGAA | 13 | 0 | 48 |
| Bi-PE-2-M-*β-Actin*+48 | GAAGCCGGCCTTGCACATGC | CCATGTATGATGCTATCGCCGCGATCGTCGTCTACAACGGCTCAGGCATGTGCAAGGCCGG | 13 | 0 | 48 |
| Bi-PE-3-M-*β-Actin* | GCTATTCTCGCAGCTCACCA | GCGAAGCCGGCCTTGCACATGCCTGAGCCGTTGTAGACGACGATCGCGGCGATAGCATCATACATGGTGAGCTGCGAGAA | 13 | 19 | 48 |
| Bi-PE-3-M-*β-Actin*+48 | GAAGCCGGCCTTGCACATGC | CGGCTATTCTCGCAGCTCACCATGTATGATGCTATCGCCGCGATCGTCGTCTACAACGGCTCAGGCATGTGCAAGGCCGG | 13 | 19 | 48 |
| *HEK3*+1-loxp-KI | GGCCCAGACTGAGCACGTGA | TGGAGGAAGCAGGGCTTCCTTTCCTCTGCCATCAATAACTTCGTATAATGTATGCTATACGAAGTTATGATATCCGTGCTCAGTCTG | 13 | 34 | 40 |
| *HEK3*+90loxp-KI | GTCAACCAGTATCCCGGTGC | TCCTAGACAGGGGCTAGTATGTGCAGCTCCTGCAATAACTTCGTATAGCATACATTATACGAAGTTATGATATCCCGGGATACTGGT | 13 | 34 | 40 |
| *HEK3*+198loxp-KI | TCTGTTGAGCTCGACCCTGA | AGCCTCACCAGGAGAGGAGGGACCTGGCCCTTCAATAACTTCGTATAGCATACATTATACGAAGTTATGATATCGGGTCGAGCTCAAC | 14 | 34 | 40 |
| *HEK3*-Δ654 8-nt HA peg | GGCCCAGACTGAGCACGTGA | GGGACCCTCGTGCTCAGTCTG | 13 | 8 | 0 |
| *HEK3*-Δ654 12-nt HA peg | GGCCCAGACTGAGCACGTGA | CAGAGGGACCCTCGTGCTCAGTCTG | 13 | 12 | 0 |
| *HEK3*-Δ654 16-nt HA peg | GGCCCAGACTGAGCACGTGA | GAGTCAGAGGGACCCTCGTGCTCAGTCTG | 13 | 16 | 0 |
| *HEK3*-Δ654 20-nt HA peg | GGCCCAGACTGAGCACGTGA | GGCTGAGTCAGAGGGACCCTCGTGCTCAGTCTG | 13 | 20 | 0 |
| *HEK3*-Δ654 25-nt HA peg | GGCCCAGACTGAGCACGTGA | ACAGGGGCTGAGTCAGAGGGACCCTCGTGCTCAGTCTG | 13 | 25 | 0 |
| *HEK3*-Δ654 30-nt HA peg | GGCCCAGACTGAGCACGTGA | AGCACACAGGGGCTGAGTCAGAGGGACCCTCGTGCTCAGTCTG | 13 | 30 | 0 |
| *HEK3*-Δ654 35-nt HA peg | GGCCCAGACTGAGCACGTGA | AACTGAGCACACAGGGGCTGAGTCAGAGGGACCCTCGTGCTCAGTCTG | 13 | 35 | 0 |
| *HEK3*-Δ654 41-nt HA peg | GGCCCAGACTGAGCACGTGA | AATTAAAACTGAGCACACAGGGGCTGAGTCAGAGGGACCCTCGTGCTCAGTCTG | 13 | 41 | 0 |
| *HEK3*-Δ654 47-nt HA peg | GGCCCAGACTGAGCACGTGA | TCCCAAAATTAAAACTGAGCACACAGGGGCTGAGTCAGAGGGACCCTCGTGCTCAGTCTG | 13 | 47 | 0 |
| *HEK3*-Δ654 51-nt HA peg | GGCCCAGACTGAGCACGTGA | TCCCTCCCAAAATTAAAACTGAGCACACAGGGGCTGAGTCAGAGGGACCCTCGTGCTCAGTCTG | 13 | 51 | 0 |
| *HEK3*+654 8-nt HA peg | TGAGTCAGAGGGACCCTTTG | TGAGCACGAGGGTCCCTCTGA | 13 | 8 | 0 |
| *HEK3*+654 12-nt HA peg | TGAGTCAGAGGGACCCTTTG | AGACTGAGCACGAGGGTCCCTCTGA | 13 | 12 | 0 |
| *HEK3*+654 16-nt HA peg | TGAGTCAGAGGGACCCTTTG | GCCCAGACTGAGCACGAGGGTCCCTCTGA | 13 | 16 | 0 |
| *HEK3*+654 20-nt HA peg | TGAGTCAGAGGGACCCTTTG | TGGGGCCCAGACTGAGCACGAGGGTCCCTCTGA | 13 | 20 | 0 |
| *HEK3*+654 25-nt HA peg | TGAGTCAGAGGGACCCTTTG | ATCCTTGGGGCCCAGACTGAGCACGAGGGTCCCTCTGA | 13 | 25 | 0 |
| *HEK3*+654 30-nt HA peg | TGAGTCAGAGGGACCCTTTG | GGTCAATCCTTGGGGCCCAGACTGAGCACGAGGGTCCCTCTGA | 13 | 30 | 0 |
| *HEK3*+654 35-nt HA peg | TGAGTCAGAGGGACCCTTTG | GCCTGGGTCAATCCTTGGGGCCCAGACTGAGCACGAGGGTCCCTCTGA | 13 | 35 | 0 |
| *HEK3*+654 41-nt HA peg | TGAGTCAGAGGGACCCTTTG | GCCCTGGCCTGGGTCAATCCTTGGGGCCCAGACTGAGCACGAGGGTCCCTCTGA | 13 | 41 | 0 |
| *HEK3*+654 47-nt HA peg | TGAGTCAGAGGGACCCTTTG | TCTCCAGCCCTGGCCTGGGTCAATCCTTGGGGCCCAGACTGAGCACGAGGGTCCCTCTGA | 13 | 47 | 0 |
| *HEK3*+654 51-nt HA peg | TGAGTCAGAGGGACCCTTTG | TGCTTCTCCAGCCCTGGCCTGGGTCAATCCTTGGGGCCCAGACTGAGCACGAGGGTCCCTCTGA | 13 | 51 | 0 |
| Bi-PE-3-*β-Actin*-Δ600 8-nt HA peg | GCTATTCTCGCAGCTCACCA | AGAGAGTCTGAGCTGCGAGAA | 13 | 8 | 0 |
| Bi-PE-3-*β-Actin*-Δ600 13-nt HA peg | GCTATTCTCGCAGCTCACCA | AGAGAAGAGAGTC TGAGCTGCGAGAA | 13 | 13 | 0 |
| Bi-PE-3-*β-Actin*-Δ600 17-nt HA peg | GCTATTCTCGCAGCTCACCA | GGTCAGAGAAGAGAGTCTGAGCTGCGAGAA | 13 | 17 | 0 |
| Bi-PE-3-*β-Actin*-Δ600 22-nt HA peg | GCTATTCTCGCAGCTCACCA | ACTCAGGTCAGAGAAGAGAGTCTGAGCTGCGAGAA | 13 | 22 | 0 |
| Bi-PE-3-*β-Actin*-Δ600 27-nt HA peg | GCTATTCTCGCAGCTCACCA | AGGAGACTCAGGTCAGAGAAGAGAGTCTGAGCTGCGAGAA | 13 | 27 | 0 |
| Bi-PE-3-*β-Actin*-Δ600 32-nt HA peg | GCTATTCTCGCAGCTCACCA | TCCAAAGGAGACTCAGGTCAGAGAAGAGAGTCTGAGCTGCGAGAA | 13 | 32 | 0 |
| Bi-PE-3-*β-Actin*-Δ600 39-nt HA peg | GCTATTCTCGCAGCTCACCA | GCAGAGTTCCAAAGGAGACTCAGGTCAGAGAAGAGAGTC TGAGCTGCGAGAA | 13 | 39 | 0 |
| Bi-PE-3-*β-Actin*-Δ600 44-nt HA peg | GCTATTCTCGCAGCTCACCA | AACCTGCAGAGTTCCAAAGGAGACTCAGGTCAGAGAAGAGAGTCTGAGCTGCGAGAA | 13 | 44 | 0 |
| Bi-PE-3-*β-Actin*-Δ600 50-nt HA peg | GCTATTCTCGCAGCTCACCA | AAATAGAACCTGCAGAGTTCCAAAGGAGACTCAGGTCAGAGAAGAGAGTCTGAGCTGCGAGAA | 13 | 50 | 0 |
| Bi-PE-3-*β-Actin*+600 8-nt HA peg | GGTCAGAGAAGAGAGTCCTA | GCAGCTCAGACTCTCTTCTCTGA | 15 | 8 | 0 |
| Bi-PE-3-*β-Actin*+600 13-nt HA peg | GGTCAGAGAAGAGAGTCCTA | TTCTCGCAGCTCA GACTCTCTTCTCTGA | 15 | 13 | 0 |
| Bi-PE-3-*β-Actin*+600 17-nt HA peg | GGTCAGAGAAGAGAGTCCTA | GCTATTCTCGCAGCTCAGACTCTCTTCTCTGA | 15 | 17 | 0 |
| Bi-PE-3-*β-Actin*+600 22-nt HA peg | GGTCAGAGAAGAGAGTCCTA | CCCGGCTATTCTCGCAGCTCAGACTCTCTTCTCTGA | 15 | 22 | 0 |
| Bi-PE-3-*β-Actin*+600 27-nt HA peg | GGTCAGAGAAGAGAGTCCTA | AGCGCGCCCGGCTATTCTCGCAGCTCA  GACTCTCTTCTCTGA | 15 | 27 | 0 |
| Bi-PE-3-*β-Actin*+600 32-nt HA peg | GGTCAGAGAAGAGAGTCCTA | CTCACAGCGCGCCCGGCTAT TCTCGCAGCTCA GACTCTCTTCTCTGA | 15 | 32 | 0 |
| Bi-PE-3-*β-Actin*+600 39-nt HA peg | GGTCAGAGAAGAGAGTCCTA | CTCACAGCGCGCCCGGCTAT TCTCGCAGCTCA GACTCTCTTCTCTGA | 15 | 39 | 0 |
| Bi-PE-3-*β-Actin*+600 44-nt HA peg | GGTCAGAGAAGAGAGTCCTA | GGGCGACCTCGGCTCACAGCGCGCCCGGCTATTCTCGCAGCTCAGACTCTCTTCTCTGA | 15 | 44 | 0 |
| Bi-PE-3-*β-Actin*+600 50-nt HA peg | GGTCAGAGAAGAGAGTCCTA | GGGCGGGGGCGACCTCGGCTCACAGCGCGCCCGGCTATTCTCGCAGCTCAACTCTCTTCTCTGA | 15 | 50 | 0 |
| *VEGFA*-Δ400 8-nt HA peg | GATGTCTGCAGGCCAGATGA | AGAGCCGGTCTGGCCTGCAGA | 13 | 8 | 0 |
| *VEGFA*-Δ400 13-nt HA peg | GATGTCTGCAGGCCAGATGA | GGCGGAGAGCCGGTCTGGCCTGCAGA | 13 | 13 | 0 |
| *VEGFA*-Δ400 17-nt HA peg | GATGTCTGCAGGCCAGATGA | GGAAGGCGGAGAGCCGGTCTGGCCTGCAGA | 13 | 17 | 0 |
| *VEGFA*-Δ400 24-nt HA peg | GATGTCTGCAGGCCAGATGA | GGGCAGGGGAAGGCGGAGAGCCGGTCTGGCCTGCAGA | 13 | 24 | 0 |
| *VEGFA*-Δ400 29-nt HA peg | GATGTCTGCAGGCCAGATGA | GAAGGGGGCAGGGGAAGGCGGAGAGCCGGTCTGGCCTGCAGA | 13 | 29 | 0 |
| *VEGFA*-Δ400 35-nt HA peg | GATGTCTGCAGGCCAGATGA | AATATTGAAGGGGGCAGGGGAAGGCGGAGAGCCGGTCTGGCCTGCAGA | 13 | 35 | 0 |
| *VEGFA*-Δ400 41-nt HA peg | GATGTCTGCAGGCCAGATGA | GCTAGGAATATTGAAGGGGGCAGGGGAAGGCGGAGAGCCGGTCTGGCCTGCAGA | 13 | 41 | 0 |
| *VEGFA*+400 8-nt HA peg | GGAAGGCGGAGAGCCGGACA | AGGCCAGACCGGCTCTCCGCC | 13 | 8 | 0 |
| *VEGFA*+400 13-nt HA peg | GGAAGGCGGAGAGCCGGACA | TCTGCAGGCCAGA CCGGCTCTCCGCC | 13 | 13 | 0 |
| *VEGFA*+400 17-nt HA peg | GGAAGGCGGAGAGCCGGACA | GATGTCTGCAGGCCAGA CCGGCTCTCCGCC | 13 | 17 | 0 |
| *VEGFA*+400 24-nt HA peg | GGAAGGCGGAGAGCCGGACA | TCACTTTGATGTCTGCAGGCCAGA CCGGCTCTCCGCC | 13 | 24 | 0 |
| *VEGFA*+400 29-nt HA peg | GGAAGGCGGAGAGCCGGACA | GCCGCTCACTTTGATGTCTGCAGGCCAGA CCGGCTCTCCGCC | 13 | 29 | 0 |
| *VEGFA*+400 35-nt HA peg | GGAAGGCGGAGAGCCGGACA | TTGGCTGCCGCTCACTTTGATGTCTGCAGGCCAGACCGGCTCTCCGCC | 13 | 35 | 0 |
| *VEGFA*+400 41-nt HA peg | GGAAGGCGGAGAGCCGGACA | AGCCTGTTGGCTGCCGCTCACTTTGATGTCTGCAGGCCAGA CCGGCTCTCCGCC | 13 | 41 | 0 |
| Bi-PE-3-  *HEK3*-  Δ654 12-nt HA peg | GGCCCAGACTGAGCACGTGA | CAGAGGGACCCTGCTGGATCCGATATCTCACGTGCTCAGTCTG | 13 | 12 | 18 |
| Bi-PE-3-*HEK3*-Δ654 16-nt HA peg | GGCCCAGACTGAGCACGTGA | GAGTCAGAGGGACCCTGCTGGATCCGATATCTCACGTGCTCAGTCTG | 13 | 16 | 18 |
| Bi-PE-3-*HEK3*-Δ654 20-nt HA peg | GGCCCAGACTGAGCACGTGA | GGCTGAGTCAGAGGGACCCTGCTGGATCCGATATCTCACGTGCTCAGTCTG | 13 | 20 | 18 |
| Bi-PE-3-*HEK3*-Δ654 25-nt HA peg | GGCCCAGACTGAGCACGTGA | ACAGGGGCTGAGTCAGAGGGACCCTGCTGGATCCGATATCTCACGTGCTCAGTCTG | 13 | 25 | 18 |
| Bi-PE-3-*HEK3*-Δ654 30-nt HA peg | GGCCCAGACTGAGCACGTGA | AGCACACAGGGGCTGAGTCAGAGGGACCCTGCTGGATCCGATATCTCACGTGCTCAGTCTG | 13 | 30 | 18 |
| Bi-PE-3-*HEK3*-Δ654 35-nt HA peg | GGCCCAGACTGAGCACGTGA | AACTGAGCACACAGGGGCTGAGTCAGAGGGACCCTGCTGGATCCGATATCTCACGTGCTCAGTCTG | 13 | 35 | 18 |
| Bi-PE-3-*HEK3*-Δ654 41-nt HA peg | GGCCCAGACTGAGCACGTGA | AATTAAAACTGAGCACACAGGGGCTGAGTCAGAGGGACCCTGCTGGATCCGATATCTCACGTGCTCAGTCTG | 13 | 41 | 18 |
| Bi-PE-3-*HEK3*-Δ654 47-nt HA peg | GGCCCAGACTGAGCACGTGA | TCCCAAAATTAAAACTGAGCACACAGGGGCTGAGTCAGAGGGACCCTGCTGGATCCGATATCTCACGTGCTCAGTCTG | 13 | 47 | 18 |
| Bi-PE-3-*HEK3*-Δ654 51-nt HA peg | GGCCCAGACTGAGCACGTGA  GGCCCAGACTGAGCACGTGA | TCCCTCCCAAAATTAAAACTGAGCACACAGGGGCTGAGTCAGAGGGACCCTGCTGGATCCGATATCTCACGTGCTCAGTCTG | 13 | 51 | 18 |
| Bi-PE-3-*HEK3*+654 12-nt HA peg | TGAGTCAGAGGGACCCTTTG | AGACTGAGCACGTGAGATATCGGATCCAGCAGGGTCCCTCTGA | 13 | 12 | 18 |
| Bi-PE-3-*HEK3*+654 16-nt HA peg | TGAGTCAGAGGGACCCTTTG | GCCCAGACTGAGCACGTGAGATATCGGATCCAGCAGGGTCCCTCTGA | 13 | 16 | 18 |
| Bi-PE-3-*HEK3*+654 20-nt HA peg | TGAGTCAGAGGGACCCTTTG | TGGGGCCCAGACTGAGCACGTGAGATATCGGATCCAGCAGGGTCCCTCTGA | 13 | 20 | 18 |
| Bi-PE-3-*HEK3*+654 25-nt HA peg | TGAGTCAGAGGGACCCTTTG | ATCCTTGGGGCCCAGACTGAGCACGTGAGATATCGGATCCAGCAGGGTCCCTCTGA | 13 | 25 | 18 |
| Bi-PE-3-*HEK3*+654 30-nt HA peg | TGAGTCAGAGGGACCCTTTG | GGTCAATCCTTGGGGCCCAGACTGAGCACGTGAGATATCGGATCCAGCAGGGTCCCTCTGA | 13 | 30 | 18 |
| Bi-PE-3-*HEK3*+654 35-nt HA peg | TGAGTCAGAGGGACCCTTTG | GCCTGGGTCAATCCTTGGGGCCCAGACTGAGCACGTGAGATATCGGATCCAGCAGGGTCCCTCTGA | 13 | 35 | 18 |
| Bi-PE-3-*HEK3*+654 41-nt HA peg | TGAGTCAGAGGGACCCTTTG | GCCCTGGCCTGGGTCAATCCTTGGGGCCCAGACTGAGCACGTGAGATATCGGATCCAGCAGGGTCCCTCTGA | 13 | 41 | 18 |
| Bi-PE-3-*HEK3*+654 47-nt HA peg | TGAGTCAGAGGGACCCTTTG | TCTCCAGCCCTGGCCTGGGTCAATCCTTGGGGCCCAGACTGAGCACGTGAGATATCGGATCCAGCAGGGTCCCTCTGA | 13 | 47 | 18 |
| Bi-PE-3-*HEK3*+654 51-nt HA peg | TGAGTCAGAGGGACCCTTTG | TGCTTCTCCAGCCCTGGCCTGGGTCAATCCTTGGGGCCCAGACTGAGCACGTGAGATATCGGATCCAGCAGGGTCCCTCTGA | 13 | 51 | 18 |
| Bi-PE-3-Bi-PE-3-*β-Actin*-Δ600 8-nt HA peg | GCTATTCTCGCAGCTCACCA | AGAGAGTCGCTGGATCCGATATCTCATGAGCTGCGAGAA | 13 | 8 | 18 |
| Bi-PE-3-*β-Actin*-Δ600 13-nt HA peg | GCTATTCTCGCAGCTCACCA | AGAGAAGAGAGTCGCTGGATCCGATATCTCA TGAGCTGCGAGAA | 13 | 13 | 18 |
| Bi-PE-3-*β-Actin*-Δ600 17-nt HA peg | GCTATTCTCGCAGCTCACCA | GGTCAGAGAAGAGAGTCGCTGGATCCGATATCTCA TGAGCTGCGAGAA | 13 | 17 | 18 |
| Bi-PE-3-*β-Actin*-Δ600 22-nt HA peg | GCTATTCTCGCAGCTCACCA | ACTCAGGTCAGAGAAGAGAGTCGCTGGATCCGATATCTCATGAGCTGCGAGAA | 13 | 22 | 18 |
| Bi-PE-3-*β-Actin*-Δ600 27-nt HA peg | GCTATTCTCGCAGCTCACCA | AGGAGACTCAGGTCAGAGAAGAGAGTC GCTGGATCCGATATCTCATGAGCTGCGAGAA | 13 | 27 | 18 |
| Bi-PE-3-*β-Actin*-Δ600 32-nt HA peg | GCTATTCTCGCAGCTCACCA | TCCAAAGGAGACTCAGGTCAGAGAAGAGAGTCGCTGGATCCGATATCTCATGAGCTGCGAGAA | 13 | 32 | 18 |
| Bi-PE-3-*β-Actin*-Δ600 39-nt HA peg | GCTATTCTCGCAGCTCACCA | GCAGAGTTCCAAAGGAGACTCAGGTCAGAGAAGAGAGTCGCTGGATCCGATATCTCA TGAGCTGCGAGAA | 13 | 39 | 13 |
| Bi-PE-3-*β-Actin*-Δ600 44-nt HA peg | GCTATTCTCGCAGCTCACCA | AACCTGCAGAGTTCCAAAGGAGACTCAGGTCAGAGAAGAGAGTCGCTGGATCCGATATCTCA TGAGCTGCGAGAA | 13 | 44 | 13 |
| Bi-PE-3-*β-Actin*+600 8-nt HA peg | GGTCAGAGAAGAGAGTCCTA | GCAGCTCATGAGATATCGGATCCAGC GACTCTCTTCTCTGA | 13 | 8 | 18 |
| Bi-PE-3-*β-Actin*+600 13-nt HA peg | GGTCAGAGAAGAGAGTCCTA | TTCTCGCAGCTCATGAGATATCGGATCCAGC GACTCTCTTCTCTGA | 13 | 13 | 18 |
| Bi-PE-3-*β-Actin*+600 17-nt HA peg | GGTCAGAGAAGAGAGTCCTA | GCTATTCTCGCAGCTCATGAGATATCGGATCCAGC GACTCTCTTCTCTGA | 13 | 17 | 18 |
| Bi-PE-3-*β-Actin*+600 22-nt HA peg | GGTCAGAGAAGAGAGTCCTA | CCCGGCTATTCTCGCAGCTCATGAGATATCGGATCCAGCGACTCTCTTCTCTGA | 13 | 22 | 18 |
| Bi-PE-3-*β-Actin*+600 27-nt HA peg | GGTCAGAGAAGAGAGTCCTA | AGCGCGCCCGGCTATTCTCGCAGCTCA  TGAGATATCGGATCCAGCGACTCTCTTCTCTGA | 13 | 27 | 18 |
| Bi-PE-3-*β-Actin*+600 32-nt HA peg | GGTCAGAGAAGAGAGTCCTA | CTCACAGCGCGCCCGGCTATTCTCGCAGCTCATGAGATATCGGATCCAGCGACTCTCTTCTCTGA | 13 | 32 | 18 |
| Bi-PE-3-*β-Actin*+600 39-nt HA peg | GGTCAGAGAAGAGAGTCCTA | ACCTCGGCTCACAGCGCGCCCGGCTATTCTCGCAGCTCATGAGATATCGGATCCAGC GACTCTCTTCTCTGA | 13 | 39 | 18 |
| Bi-PE-3-*β-Actin*+600 44-nt HA peg | GGTCAGAGAAGAGAGTCCTA | GGGCGACCTCGGCTCACAGCGCGCCCGGCTATTCTCGCAGCTCATGAGATATCGGATCCAGC GACTCTCTTCTCTGA | 13 | 44 | 18 |
| *VEGFA*-Δ400 8-nt HA peg | GATGTCTGCAGGCCAGATGA | AGAGCCGGGCTGGATCCGATATCTCATCTGGCCTGCAGA | 13 | 8 | 18 |
| *VEGFA*-Δ400 13-nt HA peg | GATGTCTGCAGGCCAGATGA | GGCGGAGAGCCGGGCTGGATCCGATATCTCATCTGGCCTGCAGA | 13 | 13 | 18 |
| *VEGFA*-Δ400 17-nt HA peg | GATGTCTGCAGGCCAGATGA | GGAAGGCGGAGAGCCGGGCTGGATCCGATATCTCATCTGGCCTGCAGA | 13 | 17 | 18 |
| *VEGFA*-Δ400 24-nt HA peg | GATGTCTGCAGGCCAGATGA | GGGCAGGGGAAGGCGGAGAGCCGGGCTGGATCCGATATCTCATCTGGCCTGCAGA | 13 | 24 | 18 |
| *VEGFA*-Δ400 29-nt HA peg | GATGTCTGCAGGCCAGATGA | GAAGGGGGCAGGGGAAGGCGGAGAGCCGGGCTGGATCCGATATCTCATCTGGCCTGCAGA | 13 | 29 | 18 |
| *VEGFA*-Δ400 35-nt HA peg | GATGTCTGCAGGCCAGATGA | AATATTGAAGGGGGCAGGGGAAGGCGGAGAGCCGGGCTGGATCCGATATCTCATCTGGCCTGCAGA | 13 | 35 | 18 |
| *VEGFA*-Δ400 41-nt HA peg | GATGTCTGCAGGCCAGATGA | GCTAGGAATATTGAAGGGGGCAGGGGAAGGCGGAGAGCCGGGCTGGATCCGATATCTCATCTGGCCTGCAGA | 13 | 41 | 18 |
| *VEGFA*+400 8-nt HA peg | GGAAGGCGGAGAGCCGGACA | AGGCCAGATGAGATATCGGATCCAGCCCGGCTCTCCGCC | 13 | 8 | 18 |
| *VEGFA*+400 13-nt HA peg | GGAAGGCGGAGAGCCGGACA | TCTGCAGGCCAGATGAGATATCGGATCCAGCCCGGCTCTCCGCC | 13 | 13 | 18 |
| *VEGFA*+400 17-nt HA peg | GGAAGGCGGAGAGCCGGACA | GATGTCTGCAGGCCAGATGAGATATCGGATCCAGCCCGGCTCTCCGCC | 13 | 17 | 18 |
| *VEGFA*+400 24-nt HA peg | GGAAGGCGGAGAGCCGGACA | TCACTTTGATGTCTGCAGGCCAGATGAGATATCGGATCCAGC CCGGCTCTCCGCC | 13 | 24 | 18 |
| *VEGFA*+400 29-nt HA peg | GGAAGGCGGAGAGCCGGACA | GCCGCTCACTTTGATGTCTGCAGGCCAGATGAGATATCGGATCCAGCCCGGCTCTCCGCC | 13 | 29 | 18 |
| *VEGFA*+400 35-nt HA peg | GGAAGGCGGAGAGCCGGACA | TTGGCTGCCGCTCACTTTGATGTCTGCAGGCCAGATGAGATATCGGATCCAGCCCGGCTCTCCGCC | 13 | 35 | 18 |
| *VEGFA*+400 41-nt HA peg | GGAAGGCGGAGAGCCGGACA | AGCCTGTTGGCTGCCGCTCACTTTGATGTCTGCAGGCCAGATGAGATATCGGATCCAGCCCGGCTCTCCGCC | 13 | 41 | 18 |
| *HEK3*-Δ654 8-nt Edit peg | GGCCCAGACTGAGCACGTGA | GGCTGAGTCAGAGGGACCCTCGTGCTCAGTCTG | 13 | 20 | 8 |
| *HEK3*-Δ654 15-nt Edit peg | GGCCCAGACTGAGCACGTGA | GGCTGAGTCAGAGGGACCCTGCCCTTGCTCACCATCGTGCTCAGTCTG | 13 | 20 | 15 |
| *HEK3*-Δ654 25-nt Edit peg | GGCCCAGACTGAGCACGTGA | GGCTGAGTCAGAGGGACCCTACAGCTCCTCGCCCTTGCTCACCATCGTGCTCAGTCTG | 13 | 20 | 25 |
| *HEK3*-Δ654 60-nt Edit peg | GGCCCAGACTGAGCACGTGA | GGCTGAGTCAGAGGGACCCTGTCCAGCTCGACCAGGATGGGCACCACCCCGGTGAACAGCTCCTCGCCCTTGCTCACCATCGTGCTCAGTCTG | 13 | 20 | 60 |
| *HEK3*-Δ654 98-nt Edit peg | GGCCCAGACTGAGCACGTGA | GGCTGAGTCAGAGGGACCCTTCGCCGGACACGCTGAACTTGTGGCCGTTTACGTCGCCGTCCAGCTCGACCAGGATGGGCACCACCCCGGTGAACAGCTCCTCGCCCTTGCTCACCATCGTGCTCAGTCTG | 13 | 20 | 98 |
| *HEK3*+654 8-nt Edit peg | TGAGTCAGAGGGACCCTTTG | TGGGGCCCAGACTGAGCACGATGGTGAG AGGGTCCCTCTGA | 13 | 20 | 8 |
| *HEK3*+654 15-nt Edit peg | TGAGTCAGAGGGACCCTTTG | TGGGGCCCAGACTGAGCACGATGGTGAGCAAGGGCAGGGTCCCTCTGA | 13 | 20 | 15 |
| *HEK3*+654 25-nt Edit peg | TGAGTCAGAGGGACCCTTTG | TGGGGCCCAGACTGAGCACGATGGTGAGCAAGGGCGAGGAGCTGTAGGGTCCCTCTGA | 13 | 20 | 25 |
| *HEK3*+654 60-nt Edit peg | TGAGTCAGAGGGACCCTTTG | TGGGGCCCAGACTGAGCACGATGGTGAGCAAGGGCGAGGAGCTGTTCACCGGGGTGGTGCCCATCCTGGTCGAGCTGGACAGGGTCCCTCTGA | 13 | 20 | 60 |
| *HEK3*+654 98-nt Edit peg | TGAGTCAGAGGGACCCTTTG | TGGGGCCCAGACTGAGCACGATGGTGAGCAAGGGCGAGGAGCTGTTCACCGGGGTGGTGCCCATCCTGGTCGAGCTGGACGGCGACGTAAACGGCCACAAGTTCAGCGTGTCCGGCGAAGGGTCCCTCTGA | 13 | 20 | 98 |
| *β-Actin*-Δ600 9-nt Edit peg | GCTATTCTCGCAGCTCACCA | AGGAGACTCAGGTCAGAGAAGAGAGTCTCCAAGCTTTGAGCTGCGAGAA | 13 | 27 | 9 |
| *β-Actin*-Δ600 13-nt Edit peg | GCTATTCTCGCAGCTCACCA | AGGAGACTCAGGTCAGAGAAGAGAGTC GGGATCCAAGCTTTGAGCTGCGAGAA | 13 | 27 | 13 |
| *β-Actin*-Δ600 17-nt Edit peg | GCTATTCTCGCAGCTCACCA | AGGAGACTCAGGTCAGAGAAGAGAGTC ACAAGGGATCCAAGCTTTGAGCTGCGAGAA | 13 | 27 | 17 |
| *β-Actin*-Δ600 25-nt Edit peg | GCTATTCTCGCAGCTCACCA | AGGAGACTCAGGTCAGAGAAGAGAGTC TGACGATGACAAGGGATCCAAGCTTTGAGCTGCGAGAA | 13 | 27 | 25 |
| *β-Actin*-Δ600 35-nt Edit peg | GCTATTCTCGCAGCTCACCA | AGGAGACTCAGGTCAGAGAAGAGAGTC  ATTACAAGGATGACGATGACAAGGGATCC AAGCTTTGAGCTGCGAGAA | 13 | 27 | 35 |
| *β-Actin*-Δ600 50-nt Edit peg | GCTATTCTCGCAGCTCACCA | AAGGAGACTCAGGTCAGAGAAGAGAGTC  GTCCAGGGTGCTGCGATTACAAGGATGACGATGACAAGGGATCCAAGCTTTGAGCTGCGAGAA | 13 | 27 | 50 |
| *β-Actin*-Δ600 100-nt Edit peg | GCTATTCTCGCAGCTCACCA | AGGAGACTCAGGTCAGAGAAGAGAGTC  GATATCATTGTTATAACTTCGTATAGCATACATTATACGAAGTTATTGCTGTCCAGGGTGCTGCGATTACAAGGATGACGATGACAAGGGATCCAAGCTTTGAGCTGCGAGAA | 13 | 27 | 100 |
| *β-Actin*-Δ600 9-nt Edit peg | GGTCAGAGAAGAGAGTCCTA | AGCGCGCCCGGCTATTCTCGCAGCTCAAAGCTTGGA GACTCTCTTCTCTGA | 15 | 27 | 9 |
| *β-Actin*-Δ600 13-nt Edit peg | GGTCAGAGAAGAGAGTCCTA | AGCGCGCCCGGCTATTCTCGCAGCTCAAAGCTTGGATCCC GACTCTCTTCTCTGA | 15 | 27 | 13 |
| *β-Actin*-Δ600 17-nt Edit peg | GGTCAGAGAAGAGAGTCCTA | AGCGCGCCCGGCTATTCTCGCAGCTCAAAGCTTGGATCCCTTGTGACTCTCTTCTCTGA | 15 | 27 | 17 |
| *β-Actin*-Δ600 25-nt Edit peg | GGTCAGAGAAGAGAGTCCTA | AGCGCGCCCGGCTATTCTCGCAGCTCAAAGCTTGGATCCCTTGTCATCGTCAGACTCTCTTCTCTGA | 15 | 27 | 25 |
| *β-Actin*-Δ600 35-nt Edit peg | GGTCAGAGAAGAGAGTCCTA | AGCGCGCCCGGCTATTCTCGCAGCTCAAAGCTTGGATCCCTTGTCATCGTCATCCTTGTAATGACTCTCTTCTCTGA | 15 | 27 | 35 |
| *β-Actin*-Δ600 50-nt Edit peg | GGTCAGAGAAGAGAGTCCTA | AGCGCGCCCGGCTATTCTCGCAGCTCAAAGCTTGGATCCCTTGTCATCGTCATCCTTGTAATCGCAGCACCCTGGACGACTCTCTTCTCTGA | 15 | 27 | 50 |
| *β-Actin*-Δ600 100-nt Edit peg | GGTCAGAGAAGAGAGTCCTA | AGCGCGCCCGGCTATTCTCGCAGCTCA  AAGCTTGGATCCCTTGTCATCGTCATCCTTGTAATCGCAGCACCCTGGACAGCAATAACTTCGTATAATGTATGCTATACGAAGTTATAACAATGATATC GACTCTCTTCTCTGA | 15 | 27 | 100 |
| *VEGFA*-Δ400 8-nt Edit peg | GATGTCTGCAGGCCAGATGA | GGGCAGGGGAAGGCGGAGAGCCGGCTCACCATTCTGGCCTGCAGA | 13 | 24 | 8 |
| *VEGFA*-Δ400 15-nt Edit peg | GATGTCTGCAGGCCAGATGA | GGGCAGGGGAAGGCGGAGAGCCGGGCCCTTGCTCACCATTCTGGCCTGCAGA | 13 | 24 | 15 |
| *VEGFA*-Δ400 60-nt Edit peg | GATGTCTGCAGGCCAGATGA | GGGCAGGGGAAGGCGGAGAGCCGGGTCCAGCTCGACCAGGATGGGCACCACCCCGGTGAACAGCTCCTCGCCCTTGCTCACCATTCTGGCCTGCAGA | 13 | 24 | 60 |
| *VEGFA*-Δ400 104-nt Edit peg | GATGTCTGCAGGCCAGATGA | GGGCAGGGGAAGGCGGAGAGCCGGTCGCCCTCGCCGGACACGCTGAACTTGTGGCCGTTTACGTCGCCGTCCAGCTCGACCAGGATGGGCACCACCCCGGTGAACAGCTCCTCGCCCTTGCTCACCATTCTGGCCTGCAGA | 13 | 24 | 104 |
| *VEGFA*+400 8-nt Edit peg | GGAAGGCGGAGAGCCGGACA | TCACTTTGATGTCTGCAGGCCAGAATGGTGAG CCGGCTCTCCGCC | 13 | 24 | 8 |
| *VEGFA*+400 15-nt Edit peg | GGAAGGCGGAGAGCCGGACA | TCACTTTGATGTCTGCAGGCCAGAATGGTGAGCAAGGGC CCGGCTCTCCGCC | 13 | 24 | 15 |
| *VEGFA*+400 60-nt Edit peg | GGAAGGCGGAGAGCCGGACA | TCACTTTGATGTCTGCAGGCCAGAATGGTGAGCAAGGGCGAGGAGCTGTTCACCGGGGTGGTGCCCATCCTGGTCGAGCTGGACCCGGCTCTCCGCC | 13 | 24 | 60 |
| *VEGFA*+400 104-NT EDIT peg | GGAAGGCGGAGAGCCGGACA | TCACTTTGATGTCTGCAGGCCAGAATGGTGAGCAAGGGCGAGGAGCTGTTCACCGGGGTGGTGCCCATCCTGGTCGAGCTGGACGGCGACGTAAACGGCCACAAGTTCAGCGTGTCCGGCGAGGGCGACCGGCTCTCCGCC | 13 | 24 | 104 |
| *Hoxd*-Δ254 peg | GAGGCATACATCTCCATGGA | ACGGGAAGGAAAACATAGATGGAGATGTATG | 13 | 18 | 0 |
| *Hoxd*-Δ336 peg | GAGGCATACATCTCCATGGA | AGGGAGGGAGGGAGAGGAATGGAGATGTATG | 13 | 18 | 0 |
| *Hoxd*+254 peg | CGGGAAGGAAAACATAGCAG | ACGAGGCATACATCTCCATCTATGTTTTCCTT | 13 | 19 | 0 |
| *Hoxd*+336 peg | GGGAGGGAGGGAGAGGAAGG | ACGAGGCATACATCTCCAT TCCTCTCCCTCCC | 13 | 19 | 0 |
| Bi-PE-2-RUNX1-M peg | GCATTTTCAGGAGGAAGCGA | CGAACGAAATGACCCAAATATGCAGTCTGAACCCAGCGCTTCCTCCTGAAAAT | 15 | 0 | 38 |
| Bi-PE-3-RUNX1-M peg | GCATTTTCAGGAGGAAGCGA | TCTCATGAAGCACTGTGGGTACGAACGAAATGACCCAAATATGCAGTCTGAACCCAGCGCTTCCTCCTGAAAAT | 15 | 21 | 38 |
| Bi-PE-2-RUNX1+38-M peg | ATGAAGCACTGTGGGTACGA | CGCTGGGTTCAGACTGCATATTTGGGTCATTTCGTTCGTACCCACAGTGCTTC | 15 | 0 | 38 |
| Bi-PE-3-RUNX1+38-M peg | ATGAAGCACTGTGGGTACGA | GGGTGCATTTTCAGGAGGAAGCGCTGGGTTCAGACTGCATATTTGGGTCATTTCGTTCGTACCCACAGTGCTTC | 15 | 21 | 38 |
| Bi-PE-2-*RNF2*-M peg | GTCATCTTAGTCATTACCTG | CATGCGAACTCAGTTTAGATGAGTTAAAACGAACATCTCAG GTAATGACTAAGATG | 15 | 0 | 41 |
| Bi-PE-3-*RNF2*-M peg | GTCATCTTAGTCATTACCTG | ACTCAACCATTAAGCAAAACATGCGAACTCAGTTTAGATGAGTTAAAACGAACATCTCAG GTAATGACTAAGATG | 15 | 19 | 41 |
| Bi-PE-2-*RNF2*+41-M peg | TCAACCATTAAGCAAAACAT | CTGAGATGTTCGTTTTAACTCATCTAAACTGAGTTCGCATG TTTTGCTTAATGGTT | 15 | 0 | 41 |
| Bi-PE-3-*RNF2*+41-M peg | TCAACCATTAAGCAAAACAT | CAGTCATCTTAGTCATTACCTGAGATGTTCGTTTTAACTCATCTAAACTGAGTTCGCATGTTTTGCTTAATGGTT | 15 | 19 | 41 |
| Bi-PE-2-*HEXA*-M peg | TACCTGAACCGTATATCCTA | CCAGGGGTTCCACTAGGTAGAAAGCCTTCCACTCAGGGCTATAGGATATACGGTTCAGGT | 16 | 0 | 37 |
| Bi-PE-3-*HEXA*-M peg | TACCTGAACCGTATATCCTA | TCTGCTTTCACCTTCAAATGCCAGGGGTTCCACTAGGTAGAAAGCCTTCCACTCAGGGCTATAGGATATACGGTTCAGGT | 16 | 20 | 37 |
| Bi-PE-2-*HEXA*+44-M peg | GCTTTCACCTTCAAATGCCA | CTATAGCCCTGAGTGGAAGGCTTTCTACCTAGTGGAACCCCTGGCATTTGAAGGTGAAAG | 16 | 0 | 37 |
| Bi-PE-3-*HEXA*+44-M peg | GCTTTCACCTTCAAATGCCA | TGGTACCTGAACCGTATATCCTATAGCCCTGAGTGGAAGGCTTTCTACCTAGTGGAACCCCTGGCATTTGAAGGTGAAAG | 16 | 20 | 37 |

**Supplementary Table 2. List of the targets tested in this study**

| sgRNA | Target sequence | Oligo-F | Oligo-R | Reference |
| --- | --- | --- | --- | --- |
| *HEK3* | GGCCCAGACTGAGCACGTGA **TGG** | CACCGGCCCAGACTGAGCACGTGA | AAACTCACGTGCTCAGTCTGGGCC | 6 |
| *HEK3* +90 | GTCAACCAGTATCCCGGTGC **AGG** | CACCGTCAACCAGTATCCCGGTGC | AAACGCACCGGGATACTGGTTGAC | 6 |
| *HEK3* +198 | TCTGTTGAGCTCGACCCTGA **AGG** | CACCGTCTGTTGAGCTCGACCCTGA | AAACTCAGGGTCGAGCTCAACAGAC | This study |
| *HEK3*+372 | GCTGGGACAGAGCTGTCCTC **TGG** | CACCGCTGGGACAGAGCTGTCCTC | AAACGAGGACAGCTCTGTCCCAGC | This study |
| *HEK3*+530 | CCACTTCCAGAGAAGTTGCT **TGG** | CACCGCCACTTCCAGAGAAGTTGCT | AAACAGCAACTTCTCTGGAAGTGGC | This study |
| *HEK3*+654 | TGAGTCAGAGGGACCCTTTG **GGG** | CACCGTGAGTCAGAGGGACCCTTTG | AAACCAAAGGGTCCCTCTGACTCAC | This study |
| *HEK3*+861 | GCTTGGGGCCAGAAGTGTCC **TGG** | CACCGCTTGGGGCCAGAAGTGTCC | AAACGGACACTTCTGGCCCCAAGC | This study |
| *β-Actin* | GCTATTCTCGCAGCTCACCA **TGG** | CACCGCTATTCTCGCAGCTCACCA | AAACTGGTGAGCTGCGAGAATAGC | This study |
| *β-Actin*+315 | TGAGGATGCCTCTCTTGCTC **TGG** | CACCGTGAGGATGCCTCTCTTGCTC | AAACGAGCAAGAGAGGCATCCTCAC | This study |
| *β-Actin*+600 | GGTCAGAGAAGAGAGTCCTA **CGG** | CACCGGTCAGAGAAGAGAGTCCTA | AAACTAGGACTCTCTTCTCTGACC | This study |
| *β-Actin*+1025 | GGAGTCCATCACGATGCCAG **TGG** | CACCGGAGTCCATCACGATGCCAG | AAACCTGGCATCGTGATGGACTCC | This study |
| *VEGFA* | GATGTCTGCAGGCCAGATGA **GGG** | CACCGATGTCTGCAGGCCAGATGA | AAACTCATCTGGCCTGCAGACATC | 6 |
| *VEGFA*+400 | GGAAGGCGGAGAGCCGGACA **AGG** | CACCGGAAGGCGGAGAGCCGGACA | AAACTGTCCGGCTCTCCGCCTTCC | This study |
| *VEGFA*+700 | CCCCGCCCCCGGCCCGCCCC **GGG** | CACCGCCCCGCCCCCGGCCCGCCCC | AAACGGGGCGGGCCGGGGGCGGGGC | This study |
| *VEGFA*+1522 | GCCCGAGCTAGCACTTCTCG **CGG** | CACCGCCCGAGCTAGCACTTCTCG | AAACCGAGAAGTGCTAGCTCGGGCC | This study |
| *FANCF* | GGAATCCCTTCTGCAGCACC **TGG** | CACCGGAATCCCTTCTGCAGCACC | AAACGGTGCTGCAGAAGGGATTCC | 6 |
| *FANCF*+48 | GGGGTCCCAGGTGCTGACGT **AGG** | CACCGGGGTCCCAGGTGCTGACGT | AAACACGTCAGCACCTGGGACCCC | 6 |
| *β-Actin*+48 | GAAGCCGGCCTTGCACATGC **TGG** | CACCGAAGCCGGCCTTGCACATGC | AAACGCATGTGCAAGGCCGGCTTC | This study |
| *AAVS1*+241 | GCAGGGTGGCCACTGAGAAC **CGG** | CACCGCAGGGTGGCCACTGAGAAC | AAACGTTCTCAGTGGCCACCCTGC | This study |
| *AAVS1*+481 | GCCAGGACGGGGCTGGCTAC **TGG** | CACCGCCAGGACGGGGCTGGCTAC | AAACGTAGCCAGCCCCGTCCTGGC | This study |
| *AAVS1*+635 | ATATGTCCCAGATAGCACTG **GGG** | CACCGATATGTCCCAGATAGCACTG | AAACCAGTGCTATCTGGGACATATC | This study |
| *AAVS1*+926 | GGGCTGGGGTGGCCTCTCGT **GGG** | CACCGGGCTGGGGTGGCCTCTCGT | AAACACGAGAGGCCACCCCAGCCC | This study |
| RUNX1 | GCATTTTCAGGAGGAAGCGA **TGG** | CACCGCATTTTCAGGAGGAAGCGA | AAACTCGCTTCCTCCTGAAAATGC | 6 |
| RUNX1+38 | ATGAAGCACTGTGGGTACGA **AGG** | CACCGATGAAGCACTGTGGGTACGA | AAACTCGTACCCACAGTGCTTCATC | 6 |
| *RNF2* | GTCATCTTAGTCATTACCTG **AGG** | CACCGTCATCTTAGTCATTACCTG | AAACCAGGTAATGACTAAGATGAC | 6 |
| *RNF2*+41 | TCAACCATTAAGCAAAACAT **GGG** | CACCGTCAACCATTAAGCAAAACAT | AAACATGTTTTGCTTAATGGTTGAC | 6 |
| *HEXA* | TACCTGAACCGTATATCCTA **TGG** | CACCGTACCTGAACCGTATATCCTA | AAACTAGGATATACGGTTCAGGTAC | 6 |
| *HEXA*+44 | GCTTTCACCTTCAAATGCCA **GGG** | CACCGCTTTCACCTTCAAATGCCA | AAACTGGCATTTGAAGGTGAAAGC | 6 |
| *DMD* sgRNA | GGAGGAGTAGAAGTGATGGT **GGG** | CACCGGAGGAGTAGAAGTGATGGT | AAACACCATCACTTCTACTCCTCC | This study |
| *DMD* +302 | CACAGTGAGGTATATGGCTC **AGG** | CACCGCACAGTGAGGTATATGGCTC | AAACGAGCCATATACCTCACTGTGC | This study |
| *DMD* +482 | GAAGGTAGACATAAAATTAG **GGG** | CACCGAAGGTAGACATAAAATTAG | AAACCTAATTTTATGTCTACCTTC | This study |
| *DMD* +906 | TAGATAGTTAAGAAACTATG **GGG** | CACCGTAGATAGTTAAGAAACTATG | AAACCATAGTTTCTTAACTATCTAC | This study |
| *Hoxd* | GAGGCATACATCTCCATGGA**GGG** | CACCGAGGCATACATCTCCATGGA | AAACTCCATGGAGATGTATGCCTC | This study |
| *Hoxd*+254 | CGGGAAGGAAAACATAGCAG **AGG** | CACCGCGGGAAGGAAAACATAGCAG | AAACCTGCTATGTTTTCCTTCCCGC | This study |
| *Hoxd*+336 | GGGAGGGAGGGAGAGGAAGG **GGG** | CACCGGGAGGGAGGGAGAGGAAGG | AAACCCTTCCTCTCCCTCCCTCCC | This study |
| sgRNA | Target sequence | Oligo-F | Oligo-R | Reference |
| *HEK3* | GGCCCAGACTGAGCACGTGA **TGG** | CACCGGCCCAGACTGAGCACGTGA | AAACTCACGTGCTCAGTCTGGGCC | 6 |
| *HEK3* +90 | GTCAACCAGTATCCCGGTGC **AGG** | CACCGTCAACCAGTATCCCGGTGC | AAACGCACCGGGATACTGGTTGAC | 6 |
| *HEK3* +198 | TCTGTTGAGCTCGACCCTGA **AGG** | CACCGTCTGTTGAGCTCGACCCTGA | AAACTCAGGGTCGAGCTCAACAGAC | This study |
| *HEK3*+372 | GCTGGGACAGAGCTGTCCTC **TGG** | CACCGCTGGGACAGAGCTGTCCTC | AAACGAGGACAGCTCTGTCCCAGC | This study |
| *HEK3*+530 | CCACTTCCAGAGAAGTTGCT **TGG** | CACCGCCACTTCCAGAGAAGTTGCT | AAACAGCAACTTCTCTGGAAGTGGC | This study |
| *HEK3*+654 | TGAGTCAGAGGGACCCTTTG **GGG** | CACCGTGAGTCAGAGGGACCCTTTG | AAACCAAAGGGTCCCTCTGACTCAC | This study |
| *HEK3*+861 | GCTTGGGGCCAGAAGTGTCC **TGG** | CACCGCTTGGGGCCAGAAGTGTCC | AAACGGACACTTCTGGCCCCAAGC | This study |
| *β-Actin* | GCTATTCTCGCAGCTCACCA **TGG** | CACCGCTATTCTCGCAGCTCACCA | AAACTGGTGAGCTGCGAGAATAGC | This study |
| *β-Actin*+315 | TGAGGATGCCTCTCTTGCTC **TGG** | CACCGTGAGGATGCCTCTCTTGCTC | AAACGAGCAAGAGAGGCATCCTCAC | This study |
| *β-Actin*+600 | GGTCAGAGAAGAGAGTCCTA **CGG** | CACCGGTCAGAGAAGAGAGTCCTA | AAACTAGGACTCTCTTCTCTGACC | This study |
| *β-Actin*+1025 | GGAGTCCATCACGATGCCAG **TGG** | CACCGGAGTCCATCACGATGCCAG | AAACCTGGCATCGTGATGGACTCC | This study |
| *VEGFA* | GATGTCTGCAGGCCAGATGA **GGG** | CACCGATGTCTGCAGGCCAGATGA | AAACTCATCTGGCCTGCAGACATC | 6 |
| *VEGFA*+400 | GGAAGGCGGAGAGCCGGACA **AGG** | CACCGGAAGGCGGAGAGCCGGACA | AAACTGTCCGGCTCTCCGCCTTCC | This study |
| *VEGFA*+700 | CCCCGCCCCCGGCCCGCCCC **GGG** | CACCGCCCCGCCCCCGGCCCGCCCC | AAACGGGGCGGGCCGGGGGCGGGGC | This study |

**Supplementary Table 3. Summary of primers for amplification of each target sites.**

| Target site | Length (bps) | Forward primer | Reverse primer | Figures |
| --- | --- | --- | --- | --- |
| *HEK3* | 1057 | TGATGTGGGCTGCCTAGAAA | GCTTGGGGCCAGAAGTGTCC | Supplementary Fig. 1b |
| *HEK3* | 1419 | TCACAGTGGCAAATGAGGCT | AAGGGTTTGGGCTTCGGATA | Fig. 1c, Fig. 3b,  Supplementary Fig. 6a,  Supplementary Fig. 7a,  Supplementary Fig. 8a,  Supplementary Fig. 10a,  Supplementary Fig. 11a,  Supplementary Fig. 12a |
| *β-Actin* | 812 | CCGACCAGTGTTTGCCTTTT | TCCTTTGGAACTCTGCAGGT | Fig. 1c |
| *VEGFA* | 1139 | GGAACAAGGGCCTCTGTCTG | CTGACCGGTCCACCTAACC | Fig. 1c, Fig. 3b,  Supplementary Fig. 10a,  Supplementary Fig. 12a |
| *AAVS1* | 1104 | CTTATATTCCCAGGGCCGGT | CCGAAGAGTGAGTTTGCCAA | Fig. 1c, Fig. 2b |
| *DMD* | 1834 | GTTTTCCCATCCTCACCTGC | AGGATGCAGGAAATAGCCGA | Fig. 1c |
| *HEK3* | 1596 | TCACAGTGGCAAATGAGGCT | CTCCTTCAAGTGCCTTTGTG | Fig. 2b |
| *β-Actin* | 1691 | CTGTGTTGGCGTACAGGTCT | GGCTTCCTTTGTCCCCAATC | Fig.2b,  Supplementary Fig. 4a,  Supplementary Fig. 6a |
| *VEGFA* | 1811 | CCAGTCACTGACTAACCCCG | GCCTCACCCGTCCATGAGCC | Fig. 2b |
| *AAVS1* | 434 | CTTATATTCCCAGGGCCGGT | GGGAAGTGTAAGGAAGCTGC | Fig. 2b |
| *DMD* | 489 | CGATTCTTATTTGGTGGTGGAGA | GGGAATTCGCATGTCTGGAG | Fig. 2b |
| *DMD* | 677 | CGATTCTTATTTGGTGGTGGAGA | TTGACGGGCAATGAGAAATTTT | Fig. 2b |
| *DMD* | 1117 | CGATTCTTATTTGGTGGTGGAGA | GACCCAAGATCATCAGACACT | Fig. 2b |
| *β-Actin* | 770 | GGCTTCCTTTGTCCCCAATC | ACCTGCAGAGTTCCAAAGGA | Fig. 3b,  Supplementary Fig. 7a,  Supplementary Fig. 8a,  Supplementary Fig. 11a,  Supplementary Fig. 12a |
| *β-Actin* | 1271 | GAACACGGCTAAGTGTGCTG | GACCCGGCGCTGTTTGAAC | Fig. 3b,  Supplementary Fig. 8a |
| *HEK3* | 858 | TGCATTTGTAGGCTTGATGC | TTCCAGCTCTAGAAGGCCAC | Supplementary Fig. 4a,  Supplementary Fig. 8a |
| *VEGFA* | 568 | AAGCATCCCTGGACACTTCC | AAAGTGAGGTTACGTGCGGA | Supplementary Fig. 4a,  Supplementary Fig. 6a,  Supplementary Fig. 7a,  Supplementary Fig. 8a,  Supplementary Fig. 10a,  Supplementary Fig. 10a |
| *HEK3* | 1096 | GCTTGGCATGAGAAACCTTGG | GCTTGGGGCCAGAAGTGTCC | Supplementary Fig. 4a |
| *Hoxd* | 938 | AAGTGTCCTTCTACCAGGGC | TATTTGTTAGTACTGGGCGG | Supplementary Fig. 8a |
| *HEK3* | 288 | TGCATTTGTAGGCTTGATGC | TCTGTTGAGCTCGACCCTGA | Fig. 5b |
| *VEGFA* | 1679 | AAGCATCCCTGGACACTTCC | CCCCTCTCCTCTTCCTTCTC | Supplementary Fig. 6a |
| *β-Actin* | 1724 | CCGACCAGTGTTTGCCTTTT | CGTACAGGTCTTTGCGGATG | Supplementary Fig. 10a |
| *β-Actin* | 233 | GGCTTCCTTTGTCCCCAATC | ACGATGGAGGGGAAGACGGC | Supplementary Fig. 14 b,d |
| *FANCF* | 732 | CGTGGTTCCGGAAATTCTCG | AGATTTGGGTTCTCTCTATAGCC | Supplementary Fig. 14 b,d |
| *HEK3-KI* | 155 | GTCAATCCTTGGGGCCCA | TTCCAGCCCAGCCAAACTT | Supplementary Fig. 16b |

**Supplementary Table 4. HTS primers used for mammalian cell genomic DNA amplification.**

| Sample name | Primer Name | Sequence |
| --- | --- | --- |
| Bi-PE-*HEK3*-Δ372 bp | *HEK3*-HTS -1-few | acgttctTGCATTTGTAGGCTTGATGC |
|  | *HEK3*-HTS -372-rev | CATAGTGCCCTGCGTGTAAC |
| L-PE3-*HEK3*-Δ372 bp | *HEK3*-HTS -2-few | agaagtcTGCATTTGTAGGCTTGATGC |
|  | *HEK3*-HTS -372-rev | CATAGTGCCCTGCGTGTAAC |
| R-PE3-*HEK3*-Δ372 bp | *HEK3*-HTS -3-few  *HEK3*-HTS -372-rev | tgatcgtTGCATTTGTAGGCTTGATGC  CATAGTGCCCTGCGTGTAAC |
| Bi-PE-*HEK3*-Δ372 bp | *HEK3*-HTS -4-few  *HEK3*-HTS -372-rev | tgacaacTGCATTTGTAGGCTTGATGC CATAGTGCCCTGCGTGTAAC |
| L-PE3-*HEK3*-Δ372 bp | *HEK3*-HTS -5-few | tgagcagTGCATTTGTAGGCTTGATGC |
|  | *HEK3*-HTS -372-rev | CATAGTGCCCTGCGTGTAAC |
| R-PE3-*HEK3*-Δ372 bp | *HEK3*-HTS -6-few | tgtactcTGCATTTGTAGGCTTGATGC |
|  | *HEK3*-HTS -372-rev | CATAGTGCCCTGCGTGTAAC |
| Bi-PE-*HEK3*-Δ654 bp | *HEK3*-HTS -7-few | tgttgacTGCATTTGTAGGCTTGATGC |
|  | *HEK3*-HTS -654-rev | TTCCAGCTCTAGAAGGCCAC |
| L-PE3-*HEK3*-Δ654 bp | *HEK3*-HTS -8-few | tgcagttTGCATTTGTAGGCTTGATGC |
|  | *HEK3*-HTS -654-rev | TTCCAGCTCTAGAAGGCCAC |
| R-PE3-*HEK3*-Δ654 bp | *HEK3*-HTS -9-few | caatgtgTGCATTTGTAGGCTTGATGC |
|  | *HEK3*-HTS -654-rev | TTCCAGCTCTAGAAGGCCAC |
| Bi-PE-*HEK3*-Δ654 bp | *HEK3*-HTS -10-few | caagtagTGCATTTGTAGGCTTGATGC |
|  | *HEK3*-HTS -654-rev | TTCCAGCTCTAGAAGGCCAC |
| L-PE3-*HEK3*-Δ654 bp | *HEK3*-HTS -11-few | catcttgTGCATTTGTAGGCTTGATGC |
|  | *HEK3*-HTS -654-rev | TTCCAGCTCTAGAAGGCCAC |
| Bi-PE-*HEK3*-Δ654 bp | *HEK3*-HTS -12-few | gagttcgTGCATTTGTAGGCTTGATGC |
|  | *HEK3*-HTS -654-rev | TTCCAGCTCTAGAAGGCCAC |
| L-PE3-*HEK3*-Δ654 bp | *HEK3*-HTS -13-few | gagcgatTGCATTTGTAGGCTTGATGC |
|  | *HEK3*-HTS -654-rev | TTCCAGCTCTAGAAGGCCAC |
| R-PE3-*HEK3*-Δ654 bp | *HEK3*-HTS -14-few | gtatgcaTGCATTTGTAGGCTTGATGC |
|  | *HEK3*-HTS -654-rev | TTCCAGCTCTAGAAGGCCAC |
| Bi-PE-2-*HEK3*-Δ654 bp | *HEK3*-HTS -15-few | gtacttgTGCATTTGTAGGCTTGATGC |
|  | *HEK3*-HTS -654-rev | TTCCAGCTCTAGAAGGCCAC |
| Bi-PE-3-*HEK3*-Δ654 bp | *HEK3*-HTS -16-few | gttcaacTGCATTTGTAGGCTTGATGC |
|  | *HEK3*-HTS -654-rev | TTCCAGCTCTAGAAGGCCAC |
| Bi-PE-2-*HEK3*-Δ654 bp | *HEK3*-HTS -17-few | gttcgtaTGCATTTGTAGGCTTGATGC |
|  | *HEK3*-HTS -654-rev | TTCCAGCTCTAGAAGGCCAC |
| Bi-PE-3-*HEK3*-654 bp | *HEK3*-HTS -18-few | gttgccaTGCATTTGTAGGCTTGATGC |
|  | *HEK3*-HTS -654-rev | TTCCAGCTCTAGAAGGCCAC |
| Bi-PE-2-*HEK3*-Δ654 bp | *HEK3*-HTS -19-few | gtggtacTGCATTTGTAGGCTTGATGC |
|  | *HEK3*-HTS -654-rev | TTCCAGCTCTAGAAGGCCAC |
| Bi-PE-3-*HEK3*-Δ654 bp | *HEK3*-HTS -20-few | gcattgtTGCATTTGTAGGCTTGATGC |
|  | *HEK3*-HTS -654-rev | TTCCAGCTCTAGAAGGCCAC |
| L-PE3-E18-*HEK3*-Δ654 | *HEK3*-HTS -7-few | tgttgacTGCATTTGTAGGCTTGATGC |
|  | *HEK3*-HTS -654-rev | TTCCAGCTCTAGAAGGCCAC |
| L-PE3-E18-*HEK3*-Δ654 | *HEK3*-HTS -8-few | tgcagttTGCATTTGTAGGCTTGATGC |
|  | *HEK3*-HTS -654-rev | TTCCAGCTCTAGAAGGCCAC |
| L-PE3-E18-*HEK3*-Δ654 | *HEK3*-HTS -9-few | caatgtgTGCATTTGTAGGCTTGATGC |
|  | *HEK3*-HTS -654-rev | TTCCAGCTCTAGAAGGCCAC |
| R-PE3-E18-*HEK3*-Δ654 | *HEK3*-HTS -10-few | caagtagTGCATTTGTAGGCTTGATGC |
|  | *HEK3*-HTS -654-rev | TTCCAGCTCTAGAAGGCCAC |
| R-PE3-E18-*HEK3*-Δ654 | *HEK3*-HTS -11-few | catcttgTGCATTTGTAGGCTTGATGC |
|  | *HEK3*-HTS -654-rev | TTCCAGCTCTAGAAGGCCAC |
| R-PE3-E18-*HEK3*-Δ654 | *HEK3*-HTS -12-few | gagttcgTGCATTTGTAGGCTTGATGC |
|  | *HEK3*-HTS -654-rev | TTCCAGCTCTAGAAGGCCAC |
| Bi-PE-2-*HEK3*-Δ861bp | *HEK3*-HTS -21-few | gctaagaTGCATTTGTAGGCTTGATGC |
|  | *HEK3*-HTS -861-rev | CACTTAGCTGGCATGGCAAT |
| Bi-PE-2-*HEK3*-Δ861bp | *HEK3*-HTS -22-few | gctcattTGCATTTGTAGGCTTGATGC |
|  | *HEK3*-HTS -861-rev | CACTTAGCTGGCATGGCAAT |
| Bi-PE-2-*HEK3*-Δ861bp | *HEK3*-HTS -23-few | gctgtaaTGCATTTGTAGGCTTGATGC |
|  | *HEK3*-HTS -861-rev | CACTTAGCTGGCATGGCAAT |
| Bi-PE-3-*HEK3*-Δ861bp | *HEK3*-HTS -24-few | gcctctaTGCATTTGTAGGCTTGATGC |
|  | *HEK3*-HTS -861-rev | CACTTAGCTGGCATGGCAAT |
| Bi-PE-3-*HEK3*-Δ861bp | *HEK3*-HTS -25-few | gcgagttTGCATTTGTAGGCTTGATGC |
|  | *HEK3*-HTS -861-rev | CACTTAGCTGGCATGGCAAT |
| L-PE3-E18-*HEK3*-Δ861bp | *HEK3*-HTS -13-few | gagcgatTGCATTTGTAGGCTTGATGC |
|  | *HEK3*-HTS -861-rev | CACTTAGCTGGCATGGCAAT |
| L-PE3-E18-*HEK3*-Δ861bp | *HEK3*-HTS -14-few | gtatgcaTGCATTTGTAGGCTTGATGC |
|  | *HEK3*-HTS -861-rev | CACTTAGCTGGCATGGCAAT |
| L-PE3-E18-*HEK3*-Δ861bp | *HEK3*-HTS -15-few | gtacttgTGCATTTGTAGGCTTGATGC |
|  | *HEK3*-HTS -861-rev | CACTTAGCTGGCATGGCAAT |
| R-PE3-E18-*HEK3*-Δ861bp | *HEK3*-HTS -16-few | gttcaacTGCATTTGTAGGCTTGATGC |
|  | *HEK3*-HTS -861-rev | CACTTAGCTGGCATGGCAAT |
| R-PE3-E18-*HEK3*-Δ861bp | *HEK3*-HTS -17-few | gttcgtaTGCATTTGTAGGCTTGATGC |
|  | *HEK3*-HTS -861-rev | CACTTAGCTGGCATGGCAAT |
| R-PE3-E18-*HEK3*-Δ861bp | *HEK3*-HTS -18-few | gttgccaTGCATTTGTAGGCTTGATGC |
|  | *HEK3*-HTS -861-rev | CACTTAGCTGGCATGGCAAT |
| Bi-PE-3-*HEK3*-Δ530 bp | *HEK3*-HTS -26-few | aactaggTGCATTTGTAGGCTTGATGC |
|  | *HEK3*-HTS -530-rev | TGGCTTTAACCCTCCAGTGT |
| Bi-PE-3-*HEK3*-Δ530 bp | *HEK3*-HTS -27-few | aagatgcTGCATTTGTAGGCTTGATGC |
|  | *HEK3*-HTS -530-rev | TGGCTTTAACCCTCCAGTGT |
| R-PE3-*HEK3*-Δ654 bp | *HEK3*-HTS -28-few | acacagtTGCATTTGTAGGCTTGATGC |
|  | *HEK3*-HTS -654-rev | TTCCAGCTCTAGAAGGCCAC |
| Bi-PE-*HEK3*-Δ861 bp | *HEK3*-HTS -29-few | acagtcaTGCATTTGTAGGCTTGATGC |
|  | *HEK3*-HTS -861-rev | CACTTAGCTGGCATGGCAAT |
| L-PE3-*HEK3*-Δ861 bp | *HEK3*-HTS -30-few | actctgaTGCATTTGTAGGCTTGATGC |
|  | *HEK3*-HTS -861-rev | CACTTAGCTGGCATGGCAAT |
| R-PE3-*HEK3*-Δ861 bp | *HEK3*-HTS -31-few | actgactTGCATTTGTAGGCTTGATGC |
|  | *HEK3*-HTS -861-rev | CACTTAGCTGGCATGGCAAT |
| Bi-PE-*HEK3*-Δ861 bp | *HEK3*-HTS -32-few | agactctTGCATTTGTAGGCTTGATGC |
|  | *HEK3*-HTS -861-rev | CACTTAGCTGGCATGGCAAT |
| L-PE3-*HEK3*-Δ861 bp | *HEK3*-HTS -33-few | agtcacaTGCATTTGTAGGCTTGATGC |
|  | *HEK3*-HTS -861-rev | CACTTAGCTGGCATGGCAAT |
| R-PE3-*HEK3*-Δ861 bp | *HEK3*-HTS -34-few | tcagagaTGCATTTGTAGGCTTGATGC |
|  | *HEK3*-HTS -861-rev | CACTTAGCTGGCATGGCAAT |
| Bi-PE-*HEK3*-Δ530 bp | *HEK3*-HTS -35-few | tgactgaTGCATTTGTAGGCTTGATGC |
|  | *HEK3*-HTS -530-rev | TGGCTTTAACCCTCCAGTGT |
| Bi-PE-*HEK3*-Δ530 bp | *HEK3*-HTS -36-few | tgtcagtTGCATTTGTAGGCTTGATGC |
|  | *HEK3*-HTS -530-rev | TGGCTTTAACCCTCCAGTGT |
| Bi-PE-*HEK3*-Δ530 bp | *HEK3*-HTS -37-few | tgtcagtTGCATTTGTAGGCTTGATGC |
|  | *HEK3*-HTS -530-rev | TGGCTTTAACCCTCCAGTGT |
| Bi-PE-2-*HEK3*-Δ530 bp | *HEK3*-HTS -38-few | tgtgtcaTGCATTTGTAGGCTTGATGC |
|  | *HEK3*-HTS -530-rev | TGGCTTTAACCCTCCAGTGT |
| Bi-PE-2-*HEK3*-Δ530 bp | *HEK3*-HTS -39-few | caactgtTGCATTTGTAGGCTTGATGC |
|  | *HEK3*-HTS -530-rev | TGGCTTTAACCCTCCAGTGT |
| Bi-PE-2-*HEK3*-Δ530 bp | *HEK3*-HTS -40-few | catcagaTGCATTTGTAGGCTTGATGC |
|  | *HEK3*-HTS -530-rev | TGGCTTTAACCCTCCAGTGT |
| Bi-PE-*HEK3*-Δ861 bp | *HEK3*-HTS -1-few | acgttctTGCATTTGTAGGCTTGATGC |
|  | *HEK3*-HTS -861-rev | CATAGTGCCCTGCGTGTAAC |
| L-PE3-*HEK3*-Δ861 bp | *HEK3*-HTS -2-few | agaagtcTGCATTTGTAGGCTTGATGC |
|  | *HEK3*-HTS -861-rev | CATAGTGCCCTGCGTGTAAC |
| R-PE3-*HEK3*-Δ861 bp | *HEK3*-HTS -3-few  *HEK3*-HTS -861-rev | tgatcgtTGCATTTGTAGGCTTGATGC  CATAGTGCCCTGCGTGTAAC |
| Bi-PE-3-*HEK3*-Δ861 bp | *HEK3*-HTS -4-few  *HEK3*-HTS -861-rev | tgacaacCAAGACCTGGCTGAGCTAAC  CATAGTGCCCTGCGTGTAAC |
| Bi-PE-*HEK3*-Δ372 bp | *HEK3*-HTS -5-few | tgagcagTGCATTTGTAGGCTTGATGC |
|  | *HEK3*-HTS -372-rev | CATAGTGCCCTGCGTGTAAC |
| L-PE3-*HEK3*-Δ372 bp | *HEK3*-HTS -6-few | tgtactcTGCATTTGTAGGCTTGATGC |
|  | *HEK3*-HTS -372-rev | CATAGTGCCCTGCGTGTAAC |
| R-PE3-*HEK3*-Δ372 bp | *HEK3*-HTS -7-few | tgttgacTGCATTTGTAGGCTTGATGC |
|  | *HEK3*-HTS -372-rev | CATAGTGCCCTGCGTGTAAC |
| Bi-PE-3-*HEK3*-Δ530 bp | *HEK3*-HTS -8-few | tgcagttTGCATTTGTAGGCTTGATGC |
|  | *HEK3*-HTS -530-rev | TGGCTTTAACCCTCCAGTGT |
| L-PE3-E18-*HEK3*-Δ530 | *HEK3*-HTS -1-few | acgttctTGCATTTGTAGGCTTGATGC |
|  | *HEK3*-HTS -530-rev | TGGCTTTAACCCTCCAGTGT |
| L-PE3-E18-*HEK3*-Δ530 | *HEK3*-HTS -2-few | agaagtcTGCATTTGTAGGCTTGATGC |
|  | *HEK3*-HTS -530-rev | TGGCTTTAACCCTCCAGTGT |
| L-PE3-E18-*HEK3*-Δ530 | *HEK3*-HTS -3-few | tgatcgtTGCATTTGTAGGCTTGATGC |
|  | *HEK3*-HTS -530-rev | TGGCTTTAACCCTCCAGTGT |
| R-PE3-E18-*HEK3*-Δ530 | *HEK3*-HTS -4-few | tgacaacTGCATTTGTAGGCTTGATGC |
|  | *HEK3*-HTS -530-rev | TGGCTTTAACCCTCCAGTGT |
| R-PE3-E18-*HEK3*-Δ530 | *HEK3*-HTS -5-few | tgagcagTGCATTTGTAGGCTTGATGC |
|  | *HEK3*-HTS -530-rev | TGGCTTTAACCCTCCAGTGT |
| R-PE3-E18-*HEK3*-Δ530 | *HEK3*-HTS -6-few | tgtactcTGCATTTGTAGGCTTGATGC |
|  | *HEK3*-HTS -530-rev | TGGCTTTAACCCTCCAGTGT |
| Bi-PE-*β-Actin*-Δ1025 bp | *β-Actin*-HTS -1-few | aagcgtgGGCTTCCTTTGTCCCCAATC |
|  | *β-Actin*-HTS-1025-rev | GAGGTAGTCAGTCAGGTCCC |
| L-PE3-*β-Actin*-Δ1025 bp | *β-Actin*-HTS -2-few | attctcgGGCTTCCTTTGTCCCCAATC |
|  | *β-Actin*-HTS-1025-rev | GAGGTAGTCAGTCAGGTCCC |
| R-PE3-*β-Actin*-Δ1025 bp | *β-Actin*-HTS -3-few | attgctcGGCTTCCTTTGTCCCCAATC |
|  | *β-Actin*-HTS-1025-rev | GAGGTAGTCAGTCAGGTCCC |
| Bi-PE-2-*β-Actin*-Δ1025 bp | *β-Actin*-HTS -4-few | atcaagcGGCTTCCTTTGTCCCCAATC |
|  | *β-Actin*-HTS-1025-rev | GAGGTAGTCAGTCAGGTCCC |
| Bi-PE-3-*β-Actin*-Δ1025 bp | *β-Actin*-HTS -5-few | atccggaGGCTTCCTTTGTCCCCAATC |
|  | *β-Actin*-HTS-1025-rev | GAGGTAGTCAGTCAGGTCCC |
| Bi-PE-2-*β-Actin*-Δ1025 bp | *β-Actin*-HTS -6-few | atcgaagGGCTTCCTTTGTCCCCAATC |
|  | *β-Actin*-HTS-1025-rev | GAGGTAGTCAGTCAGGTCCC |
| Bi-PE-3-*β-Actin*-Δ1025 bp | *β-Actin*-HTS -7-few | atcgtccGGCTTCCTTTGTCCCCAATC |
|  | *β-Actin*-HTS-1025-rev | GAGGTAGTCAGTCAGGTCCC |
| Bi-PE-2-*β-Actin*-Δ1025 bp | *β-Actin*-HTS -8-few | atgcttcGGCTTCCTTTGTCCCCAATC |
|  | *β-Actin*-HTS-1025-rev | GAGGTAGTCAGTCAGGTCCC |
| L-PE3-E18-*β-Actin*-Δ1025 bp | *β-Actin*-HTS -14-few | gaacactGGCTTCCTTTGTCCCCAATC |
|  | *β-Actin*-HTS-1025-rev | GAGGTAGTCAGTCAGGTCCC |
| L-PE3-E18-*β-Actin*-Δ1025 bp | *β-Actin*-HTS -15-few | gatctcaGGCTTCCTTTGTCCCCAATC |
|  | *β-Actin*-HTS-1025-rev | GAGGTAGTCAGTCAGGTCCC |
| L-PE3-E18-*β-Actin*-Δ1025 bp | *β-Actin*-HTS -16-few | gacatctGGCTTCCTTTGTCCCCAATC |
|  | *β-Actin*-HTS-1025-rev | GAGGTAGTCAGTCAGGTCCC |
| R-PE3-E18-*β-Actin*-Δ1025 bp | *β-Actin*-HTS -17-few | gacagtaGGCTTCCTTTGTCCCCAATC |
|  | *β-Actin*-HTS-1025-rev | GAGGTAGTCAGTCAGGTCCC |
| R-PE3-E18-*β-Actin*-Δ1025 bp | *β-Actin*-HTS -18-few | gactacaGGCTTCCTTTGTCCCCAATC |
|  | *β-Actin*-HTS-1025-rev | GAGGTAGTCAGTCAGGTCCC |
| R-PE3-E18-*β-Actin*-Δ1025 bp | *β-Actin*-HTS -19-few | gactcacGGCTTCCTTTGTCCCCAATC |
|  | *β-Actin*-HTS-1025-rev | GAGGTAGTCAGTCAGGTCCC |
| Bi-PE-3-*β-Actin*-Δ1025 bp | *β-Actin*-HTS -9-few | atggcgaGGCTTCCTTTGTCCCCAATC |
|  | *β-Actin*-HTS -1025-rev | GAGGTAGTCAGTCAGGTCCC |
| Bi-PE-2-*β-Actin*-Δ600 bp | *β-Actin*-HTS -10-few | acttagcGGCTTCCTTTGTCCCCAATC |
|  | *β-Actin*-HTS -600-rev | ACCTGCAGAGTTCCAAAGGA |
| Bi-PE-3-*β-Actin*-Δ600 bp | *β-Actin*-HTS -12-few | ctgatgtGGCTTCCTTTGTCCCCAATC |
|  | *β-Actin*-HTS -600-rev | ACCTGCAGAGTTCCAAAGGA |
| Bi-PE-2-*β-Actin*-Δ600 bp | *β-Actin*-HTS -13-few | ctgtagaGGCTTCCTTTGTCCCCAATC |
|  | *β-Actin*-HTS -600-rev | ACCTGCAGAGTTCCAAAGGA |
| Bi-PE-3-*β-Actin*-Δ600 bp | *β-Actin*-HTS -14-few | gaacactGGCTTCCTTTGTCCCCAATC |
|  | *β-Actin*-HTS -600-rev | ACCTGCAGAGTTCCAAAGGA |
| Bi-PE-2-*β-Actin*-Δ600 bp | *β-Actin*-HTS -15-few | gatctcaGGCTTCCTTTGTCCCCAATC |
|  | *β-Actin*-HTS -600-rev | ACCTGCAGAGTTCCAAAGGA |
| Bi-PE-3-*β-Actin*-Δ600 bp | *β-Actin*-HTS -16-few | gacatctGGCTTCCTTTGTCCCCAATC |
|  | *β-Actin*-HTS -600-rev | ACCTGCAGAGTTCCAAAGGA |
| L-PE3-E18-*β-Actin*-Δ600 bp | *β-Actin*-HTS -7-few | atcgtccGGCTTCCTTTGTCCCCAATC |
|  | *β-Actin*-HTS -600-rev | ACCTGCAGAGTTCCAAAGGA |
| L-PE3-E18-*β-Actin*-Δ600 bp | *β-Actin*-HTS -8-few | atgcttcGGCTTCCTTTGTCCCCAATC |
|  | *β-Actin*-HTS -600-rev | ACCTGCAGAGTTCCAAAGGA |
| L-PE3-E18-*β-Actin*-Δ600 bp | *β-Actin*-HTS -9-few | atggcgaGGCTTCCTTTGTCCCCAATC |
|  | *β-Actin*-HTS -600-rev | ACCTGCAGAGTTCCAAAGGA |
| R-PE3-E18-*β-Actin*-Δ600 bp | *β-Actin*-HTS -10-few | acttagcGGCTTCCTTTGTCCCCAATC |
|  | *β-Actin*-HTS -600-rev | ACCTGCAGAGTTCCAAAGGA |
| R-PE3-E18-*β-Actin*-Δ600 bp | *β-Actin*-HTS -12-few | ctgatgttGGCTTCCTTTGTCCCCAATC |
|  | *β-Actin*-HTS -600-rev | ACCTGCAGAGTTCCAAAGGA |
| R-PE3-E18-*β-Actin*-Δ600 bp | *β-Actin*-HTS -13-few | ctgtagaGGCTTCCTTTGTCCCCAATC |
|  | *β-Actin*-HTS -600-rev | ACCTGCAGAGTTCCAAAGGA |
| Bi-PE-2-*β-Actin*-Δ315 bp | *β-Actin*-HTS -17-few | gacagtaGGCTTCCTTTGTCCCCAATC |
|  | *β-Actin*-HTS -315-rev | TTGTAGAAGGTGTGGTGCCA |
| Bi-PE-3-*β-Actin*-Δ315 bp | *β-Actin*-HTS -18-few | gactacaGGCTTCCTTTGTCCCCAATC |
|  | *β-Actin*-HTS -315-rev | TTGTAGAAGGTGTGGTGCCA |
| Bi-PE-2-*β-Actin*-Δ315 bp | *β-Actin*-HTS -19-few | gacagtaGGCTTCCTTTGTCCCCAATC |
|  | *β-Actin*-HTS -315-rev | TTGTAGAAGGTGTGGTGCCA |
| Bi-PE-3-*β-Actin*-Δ315 bp | *β-Actin*-HTS -20-few | gactacaGGCTTCCTTTGTCCCCAATC |
|  | *β-Actin*-HTS -315-rev | TTGTAGAAGGTGTGGTGCCA |
| Bi-PE-2-*β-Actin*-Δ315 bp | *β-Actin*-HTS -21-few | gacagtaGGCTTCCTTTGTCCCCAATC |
|  | *β-Actin*-HTS -315-rev | TTGTAGAAGGTGTGGTGCCA |
| Bi-PE-3-*β-Actin*-Δ315 bp | *β-Actin*-HTS -22-few | gactacaGGCTTCCTTTGTCCCCAATC |
|  | *β-Actin*-HTS -315-rev | TTGTAGAAGGTGTGGTGCCA |
| L-PE3-E18-*β-Actin*-Δ315 bp | *β-Actin*-HTS -1-few | aagcgtgGGCTTCCTTTGTCCCCAATC |
|  | *β-Actin*-HTS -315-rev | TTGTAGAAGGTGTGGTGCCA |
| L-PE3-E18-*β-Actin*-Δ315 bp | *β-Actin*-HTS -2-few | attctcgGGCTTCCTTTGTCCCCAATC |
|  | *β-Actin*-HTS -315-rev | TTGTAGAAGGTGTGGTGCCA |
| L-PE3-E18-*β-Actin*-Δ315 bp | *β-Actin*-HTS -3-few | attgctcGGCTTCCTTTGTCCCCAATC |
|  | *β-Actin*-HTS -315-rev | TTGTAGAAGGTGTGGTGCCA |
| R-PE3-E18-*β-Actin*-Δ315 bp | *β-Actin*-HTS -4-few | atcaagcGGCTTCCTTTGTCCCCAATC |
|  | *β-Actin*-HTS -315-rev | TTGTAGAAGGTGTGGTGCCA |
| R-PE3-E18-*β-Actin*-Δ315 bp | *β-Actin*-HTS -5-few | atccggaGGCTTCCTTTGTCCCCAATC |
|  | *β-Actin*-HTS -315-rev | TTGTAGAAGGTGTGGTGCCA |
| R-PE3-E18-*β-Actin*-Δ315 bp | *β-Actin*-HTS -6-few | atcgaagGGCTTCCTTTGTCCCCAATC |
|  | *β-Actin*-HTS -315-rev | TTGTAGAAGGTGTGGTGCCA |
| Bi-PE-*β-Actin*-Δ600 bp | *β-Actin*-HTS -23-few | gtcacatGGCTTCCTTTGTCCCCAATC |
|  | *β-Actin*-HTS -600-rev | ACCTGCAGAGTTCCAAAGGA |
| L-PE3-*β-Actin*-Δ600 bp | *β-Actin*-HTS -24-few | gtctagtGGCTTCCTTTGTCCCCAATC |
|  | *β-Actin*-HTS -600-rev | ACCTGCAGAGTTCCAAAGGA |
| R-PE3-*β-Actin*-Δ600 bp | *β-Actin*-HTS -25-few | gtcgtgaGGCTTCCTTTGTCCCCAATC |
|  | *β-Actin*-HTS -600-rev | ACCTGCAGAGTTCCAAAGGA |
| R-PE3-*β-Actin*-Δ600 bp | *β-Actin*-HTS -27-few | cgtccatGGCTTCCTTTGTCCCCAATC |
|  | *β-Actin*-HTS -600-rev | ACCTGCAGAGTTCCAAAGGA |
| R-PE3-*β-Actin*-Δ600 bp | *β-Actin*-HTS -28-few | gaactacGGCTTCCTTTGTCCCCAATC |
|  | *β-Actin*-HTS -600-rev | ACCTGCAGAGTTCCAAAGGA |
| Bi-PE-*β-Actin*-Δ1025 bp | *β-Actin*-HTS -29-few | gtctagtGGCTTCCTTTGTCCCCAATC |
|  | *β-Actin*-HTS-1025-rev | GAGGTAGTCAGTCAGGTCCC |
| L-PE3-*β-Actin*-Δ600 bp | *β-Actin*-HTS -1-few | aagcgtgGGCTTCCTTTGTCCCCAATC |
|  | *β-Actin*-HTS -600-rev | ACCTGCAGAGTTCCAAAGGA |
| L-PE3-*β-Actin*-Δ600 bp | *β-Actin*-HTS -2-few | attctcgGGCTTCCTTTGTCCCCAATC |
|  | *β-Actin*-HTS -600-rev | ACCTGCAGAGTTCCAAAGGA |
| Bi-PE-*β-Actin*-Δ600 bp | *β-Actin*-HTS -3-few | attgctcGGCTTCCTTTGTCCCCAATC |
|  | *β-Actin*-HTS -600-rev | ACCTGCAGAGTTCCAAAGGA |
| Bi-PE-*β-Actin*-Δ600 bp | *β-Actin*-HTS -4-few | atcaagcGGCTTCCTTTGTCCCCAATC |
|  | *β-Actin*-HTS -600-rev | ACCTGCAGAGTTCCAAAGGA |
| R-PE3-*β-Actin*-Δ1025 bp | *β-Actin*-HTS -7-few | atcgtccGGCTTCCTTTGTCCCCAATC |
|  | *β-Actin*-HTS -1025-rev | GAGGTAGTCAGTCAGGTCCC |
| L-PE3-*β-Actin*-Δ1025 bp | *β-Actin*-HTS -8-few | atgcttcGGCTTCCTTTGTCCCCAATC |
|  | *β-Actin*-HTS -1025-rev | GAGGTAGTCAGTCAGGTCCC |
| Bi-PE-*VEGFA*-Δ400 bp | *VEGFA*-HTS -1-few | accggttAAGCATCCCTGGACACTTCC |
|  | *VEGFA*-HTS -400-rev | AAAGTGAGGTTACGTGCGGA |
| L-PE3-*VEGFA*-Δ400 bp | *VEGFA*-HTS -2-few | actacgtAAGCATCCCTGGACACTTCC |
|  | *VEGFA*-HTS-400-rev | AAAGTGAGGTTACGTGCGGA |
| R-PE3-*VEGFA*-Δ400 bp | *VEGFA*-HTS -3-few | agacatgAAGCATCCCTGGACACTTCC |
|  | *VEGFA*-HTS-400-rev | AAAGTGAGGTTACGTGCGGA |
| Bi-PE-2-*VEGFA*-Δ400 bp | *VEGFA*-HTS -4-few | catcgacAAGCATCCCTGGACACTTCC |
|  | *VEGFA*-HTS-400-rev | AAAGTGAGGTTACGTGCGGA |
| Bi-PE-3-*VEGFA*-Δ400 bp | *VEGFA*-HTS -5-few | catgatcAAGCATCCCTGGACACTTCC |
|  | *VEGFA*-HTS-400-rev | AAAGTGAGGTTACGTGCGGA |
| Bi-PE-*VEGFA*-Δ400 bp | *VEGFA*-HTS -6-few | catgcaaAAGCATCCCTGGACACTTCC |
|  | *VEGFA*-HTS-400-rev | AAAGTGAGGTTACGTGCGGA |
| L-PE3-*VEGFA*-Δ400 bp | *VEGFA*-HTS -7-few | cagttacAAGCATCCCTGGACACTTCC |
|  | *VEGFA*-HTS-400-rev | AAAGTGAGGTTACGTGCGGA |
| R-PE3-*VEGFA*-Δ400 bp | *VEGFA*-HTS -8-few | ctatggtAAGCATCCCTGGACACTTCC |
|  | *VEGFA*-HTS -400-rev | AAAGTGAGGTTACGTGCGGA |
| Bi-PE-2-*VEGFA*-Δ400 bp | *VEGFA*-HTS -9-few | ctacaagAAGCATCCCTGGACACTTCC |
|  | *VEGFA*-HTS -400-rev | AAAGTGAGGTTACGTGCGGA |
| Bi-PE-3-*VEGFA*-Δ400 bp | *VEGFA*-HTS -10-few | ctacgtcAAGCATCCCTGGACACTTCC |
|  | *VEGFA*-HTS -400-rev | AAAGTGAGGTTACGTGCGGA |
| Bi-PE-*VEGFA*-Δ400 bp | *VEGFA*-HTS -11-few | ctagcacAAGCATCCCTGGACACTTCC |
|  | *VEGFA*-HTS -400-rev | AAAGTGAGGTTACGTGCGGA |
| L-PE3-*VEGFA*-Δ400 bp | *VEGFA*-HTS -12-few | cttagctAAGCATCCCTGGACACTTCC |
|  | *VEGFA*-HTS -400-rev | AAAGTGAGGTTACGTGCGGA |
| R-PE3-*VEGFA*-Δ400 bp | *VEGFA*-HTS -13few | cttggagAAGCATCCCTGGACACTTCC |
|  | *VEGFA*-HTS -400-rev | AAAGTGAGGTTACGTGCGGA |
| Bi-PE-2-*VEGFA*-Δ400 bp | *VEGFA*-HTS -14-few | ctcaacgAAGCATCCCTGGACACTTCC |
|  | *VEGFA*-HTS -400-rev | AAAGTGAGGTTACGTGCGGA |
| Bi-PE-3-*VEGFA*-Δ400 bp | *VEGFA*-HTS -15-few | ctcgtatAAGCATCCCTGGACACTTCC |
|  | *VEGFA*-HTS -400-rev | AAAGTGAGGTTACGTGCGGA |
| L-PE3-E18-*VEGFA*-Δ400 bp | *VEGFA*-HTS -1-few | accggttAAGCATCCCTGGACACTTCC |
|  | *VEGFA*-HTS -400-rev | AAAGTGAGGTTACGTGCGGA |
| L-PE3-E18-*VEGFA*-Δ400 bp | *VEGFA*-HTS -2-few | actacgtAAGCATCCCTGGACACTTCC |
|  | *VEGFA*-HTS -400-rev | AAAGTGAGGTTACGTGCGGA |
| L-PE3-E18-*VEGFA*-Δ400 bp | *VEGFA*-HTS -3-few | agacatgAAGCATCCCTGGACACTTCC |
|  | *VEGFA*-HTS -400-rev | AAAGTGAGGTTACGTGCGGA |
| R-PE3-E18-*VEGFA*-Δ400 bp | *VEGFA*-HTS -4-few | catcgacAAGCATCCCTGGACACTTCC |
|  | *VEGFA*-HTS -400-rev | AAAGTGAGGTTACGTGCGGA |
| R-PE3-E18-*VEGFA*-Δ400 bp | *VEGFA*-HTS -5-few | catgatcAAGCATCCCTGGACACTTCC |
|  | *VEGFA*-HTS -400-rev | AAAGTGAGGTTACGTGCGGA |
| R-PE3-E18-*VEGFA*-Δ400 bp | *VEGFA*-HTS -6-few | catgcaaAAGCATCCCTGGACACTTCC |
|  | *VEGFA*-HTS -400-rev | AAAGTGAGGTTACGTGCGGA |
| Bi-PE-2-*VEGFA*-Δ700 bp | *VEGFA*-HTS -16-few | ctgctaaAAGCATCCCTGGACACTTCC |
|  | *VEGFA*-HTS -16-rev | ACAGAGCGCTGGTGCTAGC |
| Bi-PE-3-*VEGFA*-Δ700 bp | *VEGFA*-HTS -17-few | ccaatgaAAGCATCCCTGGACACTTCC |
|  | *VEGFA*-HTS -700-rev | ACAGAGCGCTGGTGCTAGC |
| Bi-PE-2-*VEGFA*-Δ700 bp | *VEGFA*-HTS -18-few | ccacgatAAGCATCCCTGGACACTTCC |
|  | *VEGFA*-HTS -700-rev | ACAGAGCGCTGGTGCTAGC |
| Bi-PE-3-*VEGFA*-Δ700 bp | *VEGFA*-HTS -19-few | ccgtgaaAAGCATCCCTGGACACTTCC |
|  | *VEGFA*-HTS -400-rev | ACAGAGCGCTGGTGCTAGC |
| Bi-PE-2-*VEGFA*-Δ700 bp | *VEGFA*-HTS -20few | cgattcaAAGCATCCCTGGACACTTCC |
|  | *VEGFA*-HTS -700-rev | ACAGAGCGCTGGTGCTAGC |
| Bi-PE-3-*VEGFA*-Δ700 bp | *VEGFA*-HTS-21-few | cgtagaaAAGCATCCCTGGACACTTCC |
|  | *VEGFA*-HTS -700-rev | ACAGAGCGCTGGTGCTAGC |
| L-PE3-E18-*VEGFA*-Δ700 bp | *VEGFA*-HTS-7-few | cagttacAAGCATCCCTGGACACTTCC |
|  | *VEGFA*-HTS -700-rev | ACAGAGCGCTGGTGCTAGC |
| L-PE3-E18-*VEGFA*-Δ700 bp | *VEGFA*-HTS-8-few | ctatggtAAGCATCCCTGGACACTTCC |
|  | *VEGFA*-HTS -700-rev | ACAGAGCGCTGGTGCTAGC |
| L-PE3-E18-*VEGFA*-Δ700 bp | *VEGFA*-HTS-9-few | ctacaagAAGCATCCCTGGACACTTCC |
|  | *VEGFA*-HTS -700-rev | ACAGAGCGCTGGTGCTAGC |
| R-PE3-E18-*VEGFA*-Δ700 bp | *VEGFA*-HTS-10-few | ctacgtcAAGCATCCCTGGACACTTCC |
|  | *VEGFA*-HTS -700-rev | ACAGAGCGCTGGTGCTAGC |
| L-PE3-E18-*VEGFA*-Δ700 bp | *VEGFA*-HTS-11-few | ctagcacAAGCATCCCTGGACACTTCC |
|  | *VEGFA*-HTS -700-rev | ACAGAGCGCTGGTGCTAGC |
| L-PE3-E18-*VEGFA*-Δ700 bp | *VEGFA*-HTS-12-few | cttagctAAGCATCCCTGGACACTTCC |
|  | *VEGFA*-HTS -700-rev | ACAGAGCGCTGGTGCTAGC |
| Bi-PE-*VEGFA*-Δ1522 bp | *VEGFA*-HTS -22-few | acgttctAAGCATCCCTGGACACTTCC |
|  | *VEGFA*-HTS -1522-rev | TTCCAGCGCCGAGTCGCCA |
| L-PE3-*VEGFA*-Δ1522 bp | *VEGFA*-HTS -23-few | agaagtcAAGCATCCCTGGACACTTCC |
|  | *VEGFA*-HTS-1522-rev | TTCCAGCGCCGAGTCGCCA |
| Bi-PE-2-*VEGFA*-Δ1522 bp | *VEGFA*-HTS -24few | acacagtAAGCATCCCTGGACACTTCC |
|  | *VEGFA*-HTS-1522-rev | TTCCAGCGCCGAGTCGCCA |
| Bi-PE-3-*VEGFA*-Δ1522 bp | *VEGFA*-HTS -25-few | acagtcaAAGCATCCCTGGACACTTCC |
|  | *VEGFA*-HTS -1522-rev | TTCCAGCGCCGAGTCGCCA |
| R-PE3-*VEGFA*-Δ1522 bp | *VEGFA*-HTS -26-few | actctgaAAGCATCCCTGGACACTTCC |
|  | *VEGFA*-HTS -1522-rev | TTCCAGCGCCGAGTCGCCA |
| Bi-PE-*VEGFA*-Δ1522 bp | *VEGFA*-HTS -27-few | actgactAAGCATCCCTGGACACTTCC |
|  | *VEGFA*-HTS -1522-rev | TTCCAGCGCCGAGTCGCCA |
| L-PE3-*VEGFA*-Δ1522 bp | *VEGFA*-HTS -28-few | agactctAAGCATCCCTGGACACTTCC |
|  | *VEGFA*-HTS -1522-rev | TTCCAGCGCCGAGTCGCCA |
| Bi-PE-2-*VEGFA*-Δ1522 bp | *VEGFA*-HTS -29-few | agtcacaAAGCATCCCTGGACACTTCC |
|  | *VEGFA*-HTS -1522-rev | TTCCAGCGCCGAGTCGCCA |
| Bi-PE-3-*VEGFA*-Δ1522 bp | *VEGFA*-HTS -30-few | tcagagaAAGCATCCCTGGACACTTCC |
|  | *VEGFA*-HTS -1522-rev | TTCCAGCGCCGAGTCGCCA |
| R-PE3-*VEGFA*-Δ1522 bp | *VEGFA*-HTS -31-few | tgactgaAAGCATCCCTGGACACTTCC |
|  | *VEGFA*-HTS -1522-rev | TTCCAGCGCCGAGTCGCCA |
| Bi-PE-*VEGFA*-Δ1522 bp | *VEGFA*-HTS -32-few | tgagactAAGCATCCCTGGACACTTCC |
|  | *VEGFA*-HTS -1522-rev | TTCCAGCGCCGAGTCGCCA |
| L-PE3-*VEGFA*-Δ1522 bp | *VEGFA*-HTS -33-few | tgtcagt AAGCATCCCTGGACACTTCC |
|  | *VEGFA*-HTS -1522-rev | TTCCAGCGCCGAGTCGCCA |
| Bi-PE-2-*VEGFA*-Δ1522 bp | *VEGFA*-HTS -34-few | tgtgtcaAAGCATCCCTGGACACTTCC |
|  | *VEGFA*-HTS -1522-rev | TTCCAGCGCCGAGTCGCCA |
| L-PE3-E18-*VEGFA*-Δ1522 bp | *VEGFA*-HTS -13-few | cttggagAAGCATCCCTGGACACTTCC |
|  | *VEGFA*-HTS -1522-rev | TTCCAGCGCCGAGTCGCCA |
| L-PE3-E18-*VEGFA*-Δ1522 bp | *VEGFA*-HTS -14-few | ctcaacgAAGCATCCCTGGACACTTCC |
|  | *VEGFA*-HTS -1522-rev | TTCCAGCGCCGAGTCGCCA |
| L-PE3-E18-*VEGFA*-Δ1522 bp | *VEGFA*-HTS -15-few | ctcgtatAAGCATCCCTGGACACTTCC |
|  | *VEGFA*-HTS -1522-rev | TTCCAGCGCCGAGTCGCCA |
| R-PE3-E18-*VEGFA*-Δ1522 bp | *VEGFA*-HTS -16-few | ctgctaaAAGCATCCCTGGACACTTCC |
|  | *VEGFA*-HTS -1522-rev | TTCCAGCGCCGAGTCGCCA |
| R-PE3-E18-*VEGFA*-Δ1522 bp | *VEGFA*-HTS -17-few | ccaatgaAAGCATCCCTGGACACTTCC |
|  | *VEGFA*-HTS -1522-rev | TTCCAGCGCCGAGTCGCCA |
| R-PE3-E18-*VEGFA*-Δ1522 bp | *VEGFA*-HTS -18-few | ccacgatAAGCATCCCTGGACACTTCC |
|  | *VEGFA*-HTS -1522-rev | TTCCAGCGCCGAGTCGCCA |
| Bi-PE-3-*VEGFA*-Δ1522 bp | *VEGFA*-HTS -35-few | caactgtAAGCATCCCTGGACACTTCC |
|  | *VEGFA*-HTS -1522-rev | TTCCAGCGCCGAGTCGCCA |
| R-PE3-*VEGFA*-Δ1522 bp | *VEGFA*-HTS -36-few | catcagaAAGCATCCCTGGACACTTCC |
|  | *VEGFA*-HTS -1522-rev | TTCCAGCGCCGAGTCGCCA |
| Bi-PE-*VEGFA*-Δ1522 bp | *VEGFA*-HTS -37-few | tgactgaAAGCATCCCTGGACACTTCC |
|  | *VEGFA*-HTS -1522-rev | TTCCAGCGCCGAGTCGCCA |
| Bi-PE-2-*FANCF*-Multipoint mutation | *FANCF*-HTS -1-few | accggttTCCAATCAGTACGCAGAGAG |
|  | *FANCF*-HTS -rev | AGGTAGCGCGCCCACTGCAA |
| Bi-PE-3-*FANCF*-Multipoint mutation | *FANCF*-HTS -2-few | actacgtTCCAATCAGTACGCAGAGAG |
|  | *FANCF*-HTS -rev | AGGTAGCGCGCCCACTGCAA |
| L-PE3-*FANCF*-Multipoint mutation | *FANCF*-HTS -3-few | agacatgTCCAATCAGTACGCAGAGAG |
|  | *FANCF*-HTS -rev | AGGTAGCGCGCCCACTGCAA |
| R-PE3-*FANCF*-Multipoint mutation | *FANCF*-HTS -4-few | catcgacTCCAATCAGTACGCAGAGAG |
|  | *FANCF*-HTS -rev | AGGTAGCGCGCCCACTGCAA |
| Untreated-*FANCF*-Multipoint mutation | *FANCF*-HTS -5-few | catgatcTCCAATCAGTACGCAGAGAG |
|  | *FANCF*-HTS -rev | AGGTAGCGCGCCCACTGCAA |
| Bi-PE-2-*FANCF*-Multipoint mutation | *FANCF*-HTS -6-few | catgcaaTCCAATCAGTACGCAGAGAG |
|  | *FANCF*-HTS -rev | AGGTAGCGCGCCCACTGCAA |
| Bi-PE-3-*FANCF*-Multipoint mutation | *FANCF*-HTS -7-few | cagttacTCCAATCAGTACGCAGAGAG |
|  | *FANCF*-HTS -rev | AGGTAGCGCGCCCACTGCAA |
| L-PE3-*FANCF*-Multipoint mutation | *FANCF*-HTS -8-few | ctatggtTCCAATCAGTACGCAGAGAG |
|  | *FANCF*-HTS -rev | AGGTAGCGCGCCCACTGCAA |
| R-PE3-FANCF-Multipoint mutation | FANCF-HTS -9-few | ctacaagTCCAATCAGTACGCAGAGAG |
|  | FANCF-HTS -rev | AGGTAGCGCGCCCACTGCAA |
| Untreated-FANCF-Multipoint mutation | FANCF-HTS -10-few | ctacgtcTCCAATCAGTACGCAGAGAG |
|  | FANCF-HTS -rev | CAAGCTGCCATTTCATTACAGG |
| Bi-PE-2-FANCF-Multipoint mutation | FANCF-HTS -11-few | ctagcacTCCAATCAGTACGCAGAGAG |
|  | FANCF-HTS -rev | AGGTAGCGCGCCCACTGCAA |
| Bi-PE-3-FANCF-Multipoint mutation | FANCF-HTS -12-few | cttagctTCCAATCAGTACGCAGAGAG |
|  | FANCF-HTS -rev | AGGTAGCGCGCCCACTGCAA |
| L-PE3-FANCF-Multipoint mutation | FANCF-HTS -13-few | cttggagTCCAATCAGTACGCAGAGAG |
|  | FANCF-HTS -rev | AGGTAGCGCGCCCACTGCAA |
| R-PE3-FANCF-Multipoint mutation | FANCF-HTS -14-few | ctcaacgTCCAATCAGTACGCAGAGAG |
|  | FANCF-HTS -rev | AGGTAGCGCGCCCACTGCAA |
| Untreated-FANCF-Multipoint mutation | FANCF-HTS -15-few | ctcgtatTCCAATCAGTACGCAGAGAG |
|  | FANCF-HTS -rev | AGGTAGCGCGCCCACTGCAA |
| Untreated-*β-Actin*-Multipoint mutation | *β-Actin*-HTS -1-few | aagcgtgGGCTTCCTTTGTCCCCAATC |
|  | *β-Actin* -HTS -rev | ACGATGGAGGGGAAGACGGC |
| Untreated-*β-Actin*-Multipoint mutation | *β-Actin*-HTS -2-few | attctcgGGCTTCCTTTGTCCCCAATC |
|  | *β-Actin* -HTS -rev | ACGATGGAGGGGAAGACGGC |
| Untreated-*β-Actin*-Multipoint mutation | *β-Actin*-HTS -3-few | attgctcGGCTTCCTTTGTCCCCAATC |
|  | *β-Actin* -HTS -rev | ACGATGGAGGGGAAGACGGC |
| Bi-PE-3-*β-Actin*-Multipoint mutation | *β-Actin*-HTS -4-few | atcaagcAAGCATCCCTGGACACTTCC |
|  | *β-Actin* -HTS -rev | TTCCAGCGCCGAGTCGCCA |
| Bi-PE-2-*β-Actin*-Multipoint mutation | *β-Actin*-HTS -5-few | atccggaAAGCATCCCTGGACACTTCC |
|  | *β-Actin* -HTS -rev | TTCCAGCGCCGAGTCGCCA |
| L-PE3-*β-Actin* -Multipoint mutation | *β-Actin*-HTS -6-few | atcgaagTCCAATCAGTACGCAGAGAG |
|  | *β-Actin* -HTS -rev | AGGTAGCGCGCCCACTGCAA |
| R-PE3-*β-Actin* -Multipoint mutation | *β-Actin*-HTS -7-few | atcgtccTCCAATCAGTACGCAGAGAG |
|  | *β-Actin* -HTS -rev | AGGTAGCGCGCCCACTGCAA |
| Bi-PE-3-*β-Actin*-Multipoint mutation | *β-Actin*-HTS -8-few | atgcttcAAGCATCCCTGGACACTTCC |
|  | *β-Actin* -HTS -rev | TTCCAGCGCCGAGTCGCCA |
| Bi-PE-2-*β-Actin*-Multipoint mutation | *β-Actin*-HTS -9-few | atggcgaAAGCATCCCTGGACACTTCC |
|  | *β-Actin* -HTS -rev | AGGTAGCGCGCCCACTGCAA |
| L-PE3-*β-Actin* -Multipoint mutation | *β-Actin*-HTS -10-few | acttagcTCCAATCAGTACGCAGAGAG |
|  | *β-Actin* -HTS -rev | AGGTAGCGCGCCCACTGCAA |
| R-PE3-*β-Actin* -Multipoint mutation | *β-Actin*-HTS -12-few | ctgatgtTCCAATCAGTACGCAGAGAG |
|  | *β-Actin* -HTS -rev | AGGTAGCGCGCCCACTGCAA |
| Bi-PE-3-*β-Actin*-Multipoint mutation | *β-Actin*-HTS -13-few | ctgtagaAAGCATCCCTGGACACTTCC |
|  | *β-Actin* -HTS -rev | TTCCAGCGCCGAGTCGCCA |
| Bi-PE-2-*β-Actin*-Multipoint mutation | *β-Actin*-HTS -14-few | gaacactAAGCATCCCTGGACACTTCC |
|  | *β-Actin* -HTS -rev | AGGTAGCGCGCCCACTGCAA |
| L-PE3-*β-Actin* -Multipoint mutation | *β-Actin*-HTS -15-few | gatctcatccAATCAGTACGCAGAGAG |
|  | *β-Actin* -HTS -rev | AGGTAGCGCGCCCACTGCAA |
| R-PE3-*β-Actin* -Multipoint mutation | *β-Actin*-HTS -16-few | gacatctTCCAATCAGTACGCAGAGAG |
|  | *β-Actin* -HTS -rev | AGGTAGCGCGCCCACTGCAA |
| Bi-PE-*AAVS1*-Δ241bp | *AAVS1*-HTS-1-few | aactgtcCTTATATTCCCAGGGCCGGT |
|  | *AAVS1*-HTS -Δ241-rev | GGGAAGTGTAAGGAAGCTGC |
| Bi-PE-*AAVS1*-Δ241bp | *AAVS1*-HTS-2-few | aacgtgtCTTATATTCCCAGGGCCGGT |
|  | *AAVS1*-HTS -Δ241-rev | GGGAAGTGTAAGGAAGCTGC |
| Bi-PE-*AAVS1*-Δ241bp | *AAVS1*-HTS-3-few | aagagctCTTATATTCCCAGGGCCGGT |
|  | *AAVS1*-HTS -Δ241-rev | GGGAAGTGTAAGGAAGCTGC |
| L-PE3-*AAVS1*-Δ241bp | *AAVS1*-HTS-4-few | aagtcagCTTATATTCCCAGGGCCGGT |
|  | *AAVS1*-HTS -Δ241-rev | GGGAAGTGTAAGGAAGCTGC |
| L-PE3-*AAVS1*-Δ241bp | *AAVS1*-HTS-5-few | atcagtgCTTATATTCCCAGGGCCGGT |
|  | *AAVS1*-HTS -Δ241-rev | GGGAAGTGTAAGGAAGCTGC |
| L-PE3-*AAVS1*-Δ241bp | *AAVS1*-HTS-6-few | atctgctCTTATATTCCCAGGGCCGGT |
|  | *AAVS1*-HTS -Δ241-rev | GGGAAGTGTAAGGAAGCTGC |
| R-PE3-*AAVS1*-Δ241bp | *AAVS1*-HTS-7-few | atgtgacCTTATATTCCCAGGGCCGGT |
|  | *AAVS1*-HTS -Δ241-rev | GGGAAGTGTAAGGAAGCTGC |
| R-PE3-*AAVS1*-Δ241bp | *AAVS1*-HTS-8-few | acacgtaCTTATATTCCCAGGGCCGGT |
|  | *AAVS1*-HTS -Δ241-rev | GGGAAGTGTAAGGAAGCTGC |
| R-PE3-*AAVS1*-Δ241bp | *AAVS1*-HTS-9-few | acagatcCTTATATTCCCAGGGCCGGT |
|  | *AAVS1*-HTS -Δ241-rev | GGGAAGTGTAAGGAAGCTGC |
| Bi-PE-*AAVS1*-Δ926bp | *AAVS1*-HTS-10-few | agatcacCTTATATTCCCAGGGCCGGT |
|  | *AAVS1*-HTS -Δ926-rev | AGGCATACAACAAAACGAATGT |
| Bi-PE-*AAVS1*-Δ926bp | *AAVS1*-HTS-11-few | agacatgCTTATATTCCCAGGGCCGGT |
|  | *AAVS1*-HTS -Δ926-rev | AGGCATACAACAAAACGAATGT |
| Bi-PE-*AAVS1*-Δ926bp | *AAVS1*-HTS-12-few | agagtgcCTTATATTCCCAGGGCCGGT |
|  | *AAVS1*-HTS -Δ926-rev | AGGCATACAACAAAACGAATGT |
| L-PE3-*AAVS1*-Δ926bp | *AAVS1*-HTS-13-few | agagctaCTTATATTCCCAGGGCCGGT |
|  | *AAVS1*-HTS -Δ926-rev | AGGCATACAACAAAACGAATGT |
| L-PE3-*AAVS1*-Δ926bp | *AAVS1*-HTS-14-few | agtacagCTTATATTCCCAGGGCCGGT |
|  | *AAVS1*-HTS -Δ926-rev | AGGCATACAACAAAACGAATGT |
| L-PE3-*AAVS1*-Δ926bp | *AAVS1*-HTS-15-few | agttcctCTTATATTCCCAGGGCCGGT |
|  | *AAVS1*-HTS -Δ926-rev | AGGCATACAACAAAACGAATGT |
| R-PE3-*AAVS1*-Δ926bp | *AAVS1*-HTS-16-few | agtctacCTTATATTCCCAGGGCCGGT |
|  | *AAVS1*-HTS -Δ926-rev | AGGCATACAACAAAACGAATGT |
| R-PE3-*AAVS1*-Δ926bp | *AAVS1*-HTS-17-few | agtcgttCTTATATTCCCAGGGCCGGT |
|  | *AAVS1*-HTS -Δ926-rev | AGGCATACAACAAAACGAATGT |
| R-PE3-*AAVS1*-Δ926bp | *AAVS1*-HTS-18-few | agcaactCTTATATTCCCAGGGCCGGT |
|  | *AAVS1*-HTS -Δ926-rev | AGGCATACAACAAAACGAATGT |
| Bi-PE-*DMD*-Δ302bp | *DMD*-HTS-1-few | catgtctCGATTCTTATTTGGTGGTGGAGA |
|  | *DMD* -HTS -Δ302-rev | GGGAATTCGCATGTCTGGAG |
| Bi-PE-*DMD*-Δ302bp | *DMD*-HTS-2-few | cacaagtCGATTCTTATTTGGTGGTGGAGA |
|  | *DMD* -HTS -Δ302-rev | GGGAATTCGCATGTCTGGAG |
| Bi-PE-*DMD*-Δ302bp | *DMD*-HTS-3-few | cacactgCGATTCTTATTTGGTGGTGGAGA |
|  | *DMD* -HTS -Δ302-rev | GGGAATTCGCATGTCTGGAG |
| L-PE3-*DMD*-Δ302bp | *DMD*-HTS-4-few | cacttgaCGATTCTTATTTGGTGGTGGAGA |
|  | *DMD* -HTS -Δ302-rev | GGGAATTCGCATGTCTGGAG |
| L-PE3-*DMD*-Δ302bp | *DMD*-HTS-5-few | cactgatCGATTCTTATTTGGTGGTGGAGA |
|  | *DMD* -HTS -Δ302-rev | GGGAATTCGCATGTCTGGAG |
| L-PE3-*DMD*-Δ302bp | *DMD*-HTS-6-few | cagatcaCGATTCTTATTTGGTGGTGGAGA |
|  | *DMD* -HTS -Δ302-rev | GGGAATTCGCATGTCTGGAG |
| R-PE3-*DMD*-Δ302bp | *DMD*-HTS-7-few | cagacatCGATTCTTATTTGGTGGTGGAGA |
|  | *DMD* -HTS -Δ302-rev | GGGAATTCGCATGTCTGGAG |
| R-PE3-*DMD*-Δ302bp | *DMD*-HTS-8-few | cagagtcCGATTCTTATTTGGTGGTGGAGA |
|  | *DMD* -HTS -Δ302-rev | GGGAATTCGCATGTCTGGAG |
| R-PE3-*DMD*-Δ302bp | *DMD*-HTS-9-few | cagtactCGATTCTTATTTGGTGGTGGAGA |
|  | *DMD* -HTS -Δ302-rev | GGGAATTCGCATGTCTGGAG |
| Bi-PE-*DMD*-Δ482bp | *DMD*-HTS-10-few | gaacactCGATTCTTATTTGGTGGTGGAGA |
|  | *DMD* -HTS -Δ482-rev | AGGCATACAACAAAACGAATGT |
| Bi-PE-*DMD*-Δ482bp | *DMD*-HTS-11-few | gatctcaCGATTCTTATTTGGTGGTGGAGA |
|  | *DMD* -HTS -Δ482-rev | AGGCATACAACAAAACGAATGT |
| Bi-PE-*DMD*-Δ482bp | *DMD*-HTS-12-few | gacatctCGATTCTTATTTGGTGGTGGAGA |
|  | *DMD* -HTS -Δ482-rev | AGGCATACAACAAAACGAATGT |
| L-PE3-*DMD*-Δ482bp | *DMD*-HTS-13-few | gactacaCGATTCTTATTTGGTGGTGGAGA |
|  | *DMD* -HTS -Δ482-rev | AGGCATACAACAAAACGAATGT |
| L-PE3-*DMD*-Δ482bp | *DMD*-HTS-14-few | gactcacCGATTCTTATTTGGTGGTGGAGA |
|  | *DMD* -HTS -Δ482-rev | AGGCATACAACAAAACGAATGT |
| L-PE3-*DMD*-Δ482bp | *DMD*-HTS-15-few | gagtcgtCGATTCTTATTTGGTGGTGGAGA |
|  | *DMD* -HTS -Δ482-rev | AGGCATACAACAAAACGAATGT |
| R-PE3-*DMD*-Δ482bp | *DMD*-HTS-16-few | gtacagaCGATTCTTATTTGGTGGTGGAGA |
|  | *DMD* -HTS -Δ482-rev | AGGCATACAACAAAACGAATGT |
| R-PE3-*DMD*-Δ482bp | *DMD*-HTS-17-few | gtagtctCGATTCTTATTTGGTGGTGGAGA |
|  | *DMD* -HTS -Δ482-rev | AGGCATACAACAAAACGAATGT |
| R-PE3-*DMD*-Δ482bp | *DMD*-HTS-18-few | gtctagtCGATTCTTATTTGGTGGTGGAGA |
|  | *DMD* -HTS -Δ482-rev | AGGCATACAACAAAACGAATGT |
| Bi-PE-*DMD*-Δ906bp | *DMD*-HTS-19-few | gtcgtgaCGATTCTTATTTGGTGGTGGAGA |
|  | *DMD* -HTS -Δ906-rev | GACCCAAGATCATCAGACACT |
| Bi-PE-*DMD*-Δ906bp | *DMD*-HTS-20-few | gtgaacaCGATTCTTATTTGGTGGTGGAGA |
|  | *DMD* -HTS -Δ906-rev | GACCCAAGATCATCAGACACT |
| Bi-PE-*DMD*-Δ906bp | *DMD*-HTS-21-few | gtgactcCGATTCTTATTTGGTGGTGGAGA |
|  | *DMD* -HTS -Δ906-rev | GACCCAAGATCATCAGACACT |
| L-PE3-*DMD*-Δ906bp | *DMD*-HTS-22-few | gtgtcaaCGATTCTTATTTGGTGGTGGAGA |
|  | *DMD* -HTS -Δ906-rev | GACCCAAGATCATCAGACACT |
| L-PE3-*DMD*-Δ906bp | *DMD*-HTS-1-few | catgtctCGATTCTTATTTGGTGGTGGAGA |
|  | *DMD* -HTS -Δ906-rev | GACCCAAGATCATCAGACACT |
| L-PE3-*DMD*-Δ906bp | *DMD*-HTS-2-few | cacaagtCGATTCTTATTTGGTGGTGGAGA |
|  | *DMD* -HTS -Δ906-rev | GACCCAAGATCATCAGACACT |
| R-PE3-*DMD*-Δ906bp | *DMD*-HTS-3-few | cacactgCGATTCTTATTTGGTGGTGGAGA |
|  | *DMD* -HTS -Δ906-rev | GACCCAAGATCATCAGACACT |
| R-PE3-*DMD*-Δ906bp | *DMD*-HTS-4-few | cacttgaCGATTCTTATTTGGTGGTGGAGA |
|  | *DMD* -HTS -Δ906-rev | GACCCAAGATCATCAGACACT |
| R-PE3-*DMD*-Δ906bp | *DMD*-HTS-5-few | cactgatCGATTCTTATTTGGTGGTGGAGA |
|  | *DMD* -HTS -Δ906-rev | GACCCAAGATCATCAGACACT |

**Supplementary Table 5. Prime editing efficiency of each replicate in Figures 1-5.**

|  | Efficiency of prime editing (%) | | | Figure |
| --- | --- | --- | --- | --- |
|  | Rep1 | Rep2 | Rep3 |  |
| TypeII-PE3-*HEK3* Δ654-bp | 13.16785872 | 6.895431836 | 10.05247482 | Fig. 1d |
| TypeII-PE3*β-Actin* Δ600-bp | 11.93880034 | 13.23316649 | 12.27122673 | Fig. 1d |
| TypeII-PE3*VEGFA* Δ400-bp | 8.064768828 | 8.493961759 | 10.41903785 | Fig. 1d |
| TypeII-PE3-*AAVS1* Δ481-bp | 25.12494899 | 10.37242584 | 9.635579654 | Fig. 1d |
| TypeII-PE3-*DMD* Δ482-bp | 5.21362908 | 2.573409869 | 1.74021573 | Fig. 1d |
| Bi-PE-*HEK3* Δ372-bp | 5.778425686 | 9.234833054 | 7.680997533 | Fig. 2c |
| L-PE3-*HEK3* Δ372-bp | 5.404753573 | 6.904518524 | 4.446695655 | Fig. 2c |
| R-PE3-*HEK3* Δ372-bp | 3.497553663 | 5.32161026 | 6.267682463 | Fig. 2c |
| Bi-PE-*HEK3* Δ530-bp | 2.342069732 | 2.732464776 | 4.771830397 | Fig. 2c |
| L-PE3-*HEK3* Δ530-bp | 1.812734534 | 0.718761057 | 1.457859221 | Fig. 2c |
| R-PE3-*HEK3* Δ530-bp | 1.590840317 | 0.774346153 | 1.969958639 | Fig. 2c |
| Bi-PE-*HEK3* Δ654-bp | 25.57732658 | 26.04086568 | 18.62485288 | Fig. 2c |
| L-PE3-*HEK3* Δ654-bp | 1.746803068 | 1.654850299 | 3.780486164 | Fig. 2c |
| R-PE3-*HEK3* Δ654-bp | 1.815084052 | 0.517937297 | 3.181493696 | Fig. 2c |
| Bi-PE-*HEK3* Δ861-bp | 37.0133455 | 36.97669457 | 20.60049631 | Fig. 2c |
| L-PE3-*HEK3* Δ861-bp | 5.046847236 | 6.224842803 | 4.584196856 | Fig. 2c |
| R-PE3-*HEK3* Δ861-bp | 4.472829362 | 12.79457448 | 4.676773458 | Fig. 2c |
| Bi-PE-*β-Actin* Δ600-bp | 11.72647208 | 12.56601484 | 9.462986936 | Fig. 2c |
| L-PE3-*β-Actin* Δ600-bp | 8.980074708 | 6.944078312 | 6.404055176 | Fig. 2c |
| R-PE3-*β-Actin* Δ600-bp | 1.107650274 | 4.670841494 | 4.540596771 | Fig. 2c |
| Bi-PE-*β-Actin* Δ1025-bp | 32.29750049 | 18.56497985 | 18.8243587 | Fig. 2c |
| L-PE3-*β-Actin* Δ1025-bp | 12.07536154 | 7.705543497 | 7.847647978 | Fig. 2c |
| R-PE3-*β-Actin* Δ1025-bp | 25.70489679 | 11.23690452 | 22.06877972 | Fig. 2c |
| Bi-PE-*VEGFA* Δ400-bp | 40.52048841 | 43.07281924 | 26.5430845 | Fig. 2c |
| L-PE3-*VEGFA* Δ400-bp | 1.099767413 | 7.867099872 | 5.319709112 | Fig. 2c |
| R-PE3-*VEGFA* Δ400-bp | 1.117003816 | 3.290340553 | 2.299651665 | Fig. 2c |
| Bi-PE-*VEGFA* Δ1522-bp | 41.92717711 | 84.6321511 | 66.62099951 | Fig. 2c |
| L-PE3-*VEGFA* Δ1522-bp | 35.68631125 | 75.05232382 | 65.40826996 | Fig. 2c |
| R-PE3-*VEGFA* Δ1522-bp | 27.98795387 | 36.08774998 | 15.79552341 | Fig. 2c |
| Bi-PE-*AAVS1* Δ241-bp | 15.1024238 | 18.5827848 | 33.04962353 | Fig. 2c |
| L-PE3-*AAVS1* Δ241-bp | 20.32463853 | 23.60161737 | 26.57000563 | Fig. 2c |
| R-PE3-*AAVS1* Δ241-bp | 20.694664 | 31.97739139 | 30.36790075 | Fig. 2c |
| Bi-PE-*AAVS1* Δ926-bp | 87.38552694 | 82.15063435 | 66.23215636 | Fig. 2c |
| L-PE3-*AAVS1* Δ926-bp | 84.83561737 | 78.10378656 | 59.27308515 | Fig. 2c |
| R-PE3-*AAVS1* Δ926-bp | 38.86184971 | 67.50750806 | 34.7461487 | Fig. 2c |
| Bi-PE-*DMD* Δ302-bp | 11.46034532 | 18.73592589 | 16.53136984 | Fig. 2c |
| L-PE3-*DMD* Δ302-bp | 10.73201863 | 18.38647097 | 17.22751316 | Fig. 2c |
| R-PE3-*DMD* Δ302-bp | 12.57799335 | 15.37930807 | 14.99332765 | Fig. 2c |
| Bi-PE-*DMD* Δ482-bp | 16.53677351 | 36.46389102 | 21.98396413 | Fig. 2c |
| L-PE3-*DMD* Δ482-bp | 18.2224767 | 25.33935828 | 23.57300221 | Fig. 2c |
| R-PE3-*DMD* Δ482-bp | 16.02040649 | 14.58347899 | 14.80222169 | Fig. 2c |
| Bi-PE-*DMD* Δ906-bp | 62.9292855 | 40.58198873 | 40.70443164 | Fig. 2c |
| L-PE3-*DMD* Δ906-bp | 52.39847205 | 29.06953914 | 27.40221285 | Fig. 2c |
| R-PE3-*DMD* Δ906-bp | 59.22882207 | 37.86653509 | 34.06925543 | Fig. 2c |
| Bi-PE-2 *HEK3* Δ530+18-bp | 6.505061553 | 2.692803567 | 5.303474282 | Fig. 3c |
| Bi-PE-3 *HEK3* Δ530+18-bp | 28.08090095 | 10.36551881 | 13.48826993 | Fig. 3c |
| L-PE3-*HEK3* Δ530+18-bp | 0 | 0 | 0 | Fig. 3c |
| R-PE3-*HEK3* Δ530+18-bp | 11.05397819 | 1.400753487 | 5.022422486 | Fig. 3c |
| Bi-PE-2 *HEK3* Δ654+18-bp | 28.89137849 | 18.96494312 | 9.8985238 | Fig. 3c |
| Bi-PE-3 *HEK3* Δ654+18-bp | 50.71580273 | 54.09860805 | 32.45874574 | Fig. 3c |
| L-PE3-*HEK3* Δ654+18-bp | 21.860765 | 28.68853135 | 5.990294786 | Fig. 3c |
| R-PE3-*HEK3* Δ654+18-bp | 10.34748876 | 29.65738242 | 10.93013141 | Fig. 3c |
| Bi-PE-2 *HEK3* Δ861+18-bp | 19.99820444 | 15.1233592 | 9.043738505 | Fig. 3c |
| Bi-PE-3 *HEK3* Δ861+18-bp | 64.78002244 | 62.5489353 | 54.93619911 | Fig. 3c |
| L-PE3-*HEK3* Δ861+18-bp | 6.654139541 | 5.406035402 | 5.874944388 | Fig. 3c |
| R-PE3-*HEK3* Δ861+18-bp | 51.10736516 | 44.08490174 | 29.95693536 | Fig. 3c |
| Bi-PE-2  *β-Actin* Δ315+18-bp | 43.78612535 | 37.36881814 | 39.86072541 | Fig. 3c |
| Bi-PE-3  *β-Actin* Δ315+18-bp | 51.02506599 | 38.19432145 | 43.21411647 | Fig. 3c |
| L-PE3-*β-Actin* Δ315+18-bp | 27.10034116 | 27.32787288 | 27.77900528 | Fig. 3c |
| R-PE3-*β-Actin* Δ315+18-bp | 31.02993418 | 26.99109478 | 30.77518808 | Fig. 3c |
| Bi-PE-2  *β-Actin* Δ600+18-bp | 29.94992657 | 48.72326524 | 18.19591528 | Fig. 3c |
| Bi-PE-3  *β-Actin* Δ600+18-bp | 29.10136295 | 50.60681983 | 24.08120915 | Fig. 3c |
| L-PE3-*β-Actin* Δ600+18-bp | 18.65683561 | 35.5526061 | 18.21191527 | Fig. 3c |
| R-PE3-*β-Actin* Δ600+18-bp | 18.86285831 | 26.78927866 | 18.99788395 | Fig. 3c |
| Bi-PE-2  *β-Actin* Δ1025+18-bp | 52.6445776 | 24.92543377 | 32.76912737 | Fig. 3c |
| Bi-PE-3  *β-Actin* Δ1025+18-bp | 60.36411377 | 26.11239364 | 33.34708253 | Fig. 3c |
| L-PE3-*β-Actin* Δ1025+18-bp | 32.42752008 | 18.01237761 | 22.60878639 | Fig. 3c |
| R-PE3-*β-Actin* Δ1025+18-bp | 24.59422009 | 16.2958739 | 22.68496123 | Fig. 3c |
| Bi-PE-2  *VEGFA* Δ400+18-bp | 7.098925417 | 13.3478597 | 4.768409904 | Fig. 3c |
| Bi-PE-3  *VEGFA* Δ400+18-bp | 45.16458201 | 55.44120044 | 45.93295365 | Fig. 3c |
| L-PE3-*VEGFA* Δ400+18-bp | 0 | 0 | 0 | Fig. 3c |
| R-PE3-*VEGFA* Δ400+18-bp | 0 | 0 | 0 | Fig. 3c |
| Bi-PE-2  *VEGFA* Δ700+18-bp | 51.45610123 | 18.21746288 | 18.57084932 | Fig. 3c |
| Bi-PE-3  *VEGFA* Δ700+18-bp | 75.86668957 | 65.29092077 | 38.98222826 | Fig. 3c |
| L-PE3-*VEGFA* Δ700+18-bp | 51.84599021 | 34.75265476 | 24.25212081 | Fig. 3c |
| R-PE3-*VEGFA* Δ700+18-bp | 74.95341703 | 63.16945921 | 43.24454434 | Fig. 3c |
| Bi-PE-2-*FANCF* | 10.74783951 | 12.10766807 | 13.17988915 | Fig. 4b |
| Bi-PE-3-*FANCF* | 18.2106599 | 12.01819049 | 23.44539994 | Fig. 4b |
| L-PE3-*FANCF* | 17.17680817 | 11.20339724 | 17.75320463 | Fig. 4b |
| R-PE3-*FANCF* | 12.62327416 | 13.63270318 | 13.68923021 | Fig. 4b |
| Bi-PE-2-*β-Actin* | 13.11999076 | 9.405185792 | 10.28706907 | Fig. 4b |
| Bi-PE-3-*β-Actin* | 16.10963545 | 12.78144591 | 13.33362966 | Fig. 4b |
| L-PE3-*β-Actin* | 11.16425026 | 7.118700314 | 11.17085752 | Fig. 4b |
| R-PE3-*β-Actin* | 9.309921785 | 9.143267263 | 8.787067209 | Fig. 4b |
| Bi-PE-2-*RUNX1* | 0.706457882 | 0.870383206 | 0.853831286 | Fig. 4b |
| Bi-PE-3-*RUNX1* | 7.824782432 | 7.745119646 | 8.873322106 | Fig. 4b |
| L-PE3-*RUNX1* | 5.334547322 | 6.107759009 | 6.275570965 | Fig. 4b |
| R-PE3-*RUNX1* | 7.378727072 | 8.133678909 | 8.247085794 | Fig. 4b |
| Bi-PE-2-*RNF2* | 1.884450981 | 2.322557768 | 2.301114527 | Fig. 4b |
| Bi-PE-3-*RNF2* | 1.989243682 | 2.88345655 | 2.231185103 | Fig. 4b |
| L-PE3-*RNF2* | 2.578158743 | 1.852595354 | 2.126744115 | Fig. 4b |
| R-PE3-*RNF2* | 0 | 0.014365457 | 0.022735258 | Fig. 4b |
| Bi-PE-2-HEXA | 0.719756772 | 0.496835313 | 0.546882725 | Fig. 4b |
| Bi-PE-3-HEXA | 3.778307828 | 4.051366252 | 4.075424776 | Fig. 4b |
| L-PE3-HEXA | 1.945738559 | 2.098153837 | 1.992765374 | Fig. 4b |
| R-PE3-HEXA | 2.204033852 | 2.863643496 | 3.358051978 | Fig. 4b |
| N=90-bp single-LoxP-KI | 22.91666667 | 32.60869565 | 28.125 | Fig. 5c |
| N=90-bp double-LoxP-KI | 13.979 | 12.5 | 17.70833333 | Fig. 5c |
| N=198-bp single-LoxP-KI | 10.9375 | 6.756756757 | 8.108108108 | Fig. 5c |
| N=198-bp double-LoxP-KI | 3.125 | 2.702702703 | 1.351351351 | Fig. 5c |

**Supplementary Note 1. Custom python script for HTS data analysis.**

# -*- coding: utf-8 -*-

import os

import time

import gzip

import openpyxl as op

import xlrd

import pandas as pd

from Bio import SeqIO

from Bio import pairwise2

from Bio.SubsMat import MatrixInfo

from Bio.pairwise2 import format_alignment

from Bio.Seq import Seq

from collections import Counter

from concurrent.futures import ThreadPoolExecutor

matrix = MatrixInfo.blosum62

file_path = '/Volumes/TR'

seq_name = '/Volumes/TR/reference_sequence2.xlsx'

def get_files():

file_list = []

for filepath, dirnames, filenames in os.walk(file_path):

for filename in filenames:

file_list.append(os.path.join(filepath, filename))

print(os.path.join(filepath, filename))

return file_list

def get_reference_sequence(seq_name):

work_book = xlrd.open_workbook(seq_name)

rs_data = {}

for i, sheet_obj in enumerate(work_book.sheets()):

if i == 0:

for row in range(sheet_obj.nrows):

if row != 0:

rs_data[sheet_obj.row_values(row)[0][1:]] = sheet_obj.row_values(row)[1].upper()

return rs_data

def counter(arr):

return Counter(arr)

def get_tcc_seq(fast_file_name):

"""reads clustering"""

rs_data = get_reference_sequence(seq_name)

with gzip.open(fast_file_name, 'rt') as fasta_file:

all_seqs = [str(fa.seq) for fa in SeqIO.parse(fasta_file, 'fastq')]

product_name = fast_file_name.split("/")[-1].split('.')[0]

cat = rs_data.get(product_name)

print("current file name：{}，sequence：{}".format(fast_file_name, cat))

exec('category_{} = []'.format(cat))

for seq_record in all_seqs:

if seq_record.startswith(cat):

exec('category_{}.append("{}")'.format(cat, seq_record))

code = """result_dic_{} = sorted(counter(category_{}).items(), key=lambda d: d[1], reverse=True)\nif result_dic_{}:\n seq1 = result_dic_{}[0][0]\n t_len = len(category_{})\n for i in result_dic_{}[:500]:\n seq2 = Seq(i[0])\n n_len = int(i[1])\n percent_match = (n_len / t_len) * 100\n alignments = pairwise2.align.localds(seq1, seq2, matrix, -10, -1)\n for alignment in alignments:\n mylog = open(fast_file_name.split(\'.\')[0] +\'_\'+ "{}" +\'_handle_datas.txt\', mode = \'a\',encoding=\'utf-8\')\n mylog.write(format_alignment(*alignment)+\'percent_match : \'+ str(n_len) + \'/\' + str(t_len)+ \' = \' + str(percent_match)+"\\n")\n mylog.close()\n data = pd.DataFrame(result_dic_{}, columns=[\'seq\', \'count\'])\n writer = pd.ExcelWriter(fast_file_name.split(\'.\')[0] +\'_\'+ "{}" + \'_datas.xlsx\')\n data.to_excel(writer, index=False)\n writer.save()\n writer.close()"""

exec(code.format(cat, cat, cat, cat, cat, cat, cat, cat, cat))

if __name__ == '__main__':

file_list = get_files()

start1 = time.time()

try:

with ThreadPoolExecutor(10) as executor1:

executor1.map(get_tcc_seq, file_list)

except Exception as e:

print(e)

end1 = time.time()

print("executing time: " + str(end1 - start1))
